# The sensitivity and specificity of a diagnostic test of sequence-space synesthesia

**DOI:** 10.3758/s13428-015-0656-2

**Published:** 2015-11-11

**Authors:** Nicolas Rothen, Kristin Jünemann, Andy D. Mealor, Vera Burckhardt, Jamie Ward

**Affiliations:** School of Psychology and Sackler Centre for Consciousness Science, University of Sussex, Brighton, UK

**Keywords:** Synesthesia/synaesthesia, Space, Numbers, Time, Imagery

## Abstract

People with sequence-space synesthesia (SSS) report stable visuo-spatial forms corresponding to numbers, days, and months (amongst others). This type of synesthesia has intrigued scientists for over 130 years but the lack of an agreed upon tool for assessing it has held back research on this phenomenon. The present study builds on previous tests by measuring the consistency of spatial locations that is known to discriminate controls from synesthetes. We document, for the first time, the sensitivity and specificity of such a test and suggest a diagnostic cut-off point for discriminating between the groups based on the area bounded by different placement attempts with the same item.

**Electronic supplementary material:**

The online version of this article (doi:10.3758/s13428-015-0656-2) contains supplementary material, which is available to authorized users.

## Introduction

Sequence-space synesthesia (SSS) involves the habitual visualization of certain sequences (such as months, days, and numbers) as arranged in an ordered spatial configuration. For instance, as one of our participants describes it:

“There are various planes: horizontal, vertical, starting on the left, starting on the right, above waist level, below waist level, and stretching out in front of me. The alphabet is pretty much upright but [the letters are] variable sizes and veers off a bit upwards right, and away from me after QRST.... Months of the year twist a bit and move depending on the starting month I am thinking about… Weeks have several forms starting on a basic Sun-Mon in front of me left to right… All these subjects are in completely separate planes. Nothing shares the same space… I suddenly realize how fixed and definite it all is and am surprised that you don’t know where these things are.” (Ward, 2008)

For some people with SSS, the sequences appear to occupy the peripersonal space outside their body and for others it is on an inner screen (Smilek, Callejas, Merikle, & Dixon, 2006). For some the vantage point with which they view the sequence is fixed but for others they can change perspective or “zoom in” (Jarick, Dixon, Stewart, Maxwell, & Smilek, 2009). For some, the sequences have idiosyncratic visual features such as shading, texture, and font (Gould, Froese, Barrett, Ward, & Seth, 2014). It is generally considered a type of synesthesia and, indeed, these types of visualizations are far more common in people who have synesthetic experiences of color (Sagiv, Simner, Collins, Butterworth, & Ward, 2006; but see Novich, Cheng, & Eagleman, 2011). The first documented case was noted by Hudson (1873) and, soon afterwards, Galton (1880a, 1880b) investigated them extensively in the domain of number, for which he used the term “number form.” Galton’s interest in them stemmed from the wider question of the functional role of mental images in cognitive ability. This approach still resonates in contemporary research in this area (e.g., Price, 2009; Simner, Mayo, & Spiller, 2009). However, before one can determine the precise functionality of SSS, a more basic scientific problem arises in terms of how one can distinguish those who report such forms of synesthetic phenomenology from those who do not report such experiences (but might have intuitive spatial associations; Fias & Fischer, 2005).

For synesthetic color experiences, measures of consistency over time have become a convenient and reliable diagnostic tool. This involves presenting the same stimulus (e.g., a digit) on multiple occasions, measuring the associated color, and then calculating the difference between each attempt. Earlier studies used verbal color descriptions, long test-retest intervals (weeks or months), and measured item consistency in terms of a binary consistent/inconsistent measure (Baron-Cohen, Harrison, Goldstein, & Wyke, 1993). Most contemporary studies use computer-based color selections, involve retests within the same session, and measure distances within color space (Eagleman, Kagan, Nelson, Sagaram, & Sarma, 2007).

Many attempts to establish the authenticity of sequence-space synesthesia have also used measures of consistency in which locations in space are measured. In some studies, synesthetes are asked to imagine a 2D rendering of the spatial form on a computer screen and are then prompted with cues (e.g., “Tuesday”) to localize that stimulus on the screen (Brang, Teuscher, Ramachandran, & Coulson, 2010; Smilek et al., 2006). The cues are presented multiple times and in random orders. In other studies, synesthetes have been asked to point to the location in egocentric space, and angular displacements are used as measures of consistency (Smilek et al., 2006) or synesthetes have been asked to project the locations around a virtual 3D body on a computer display (Eagleman, 2009). Sequence-space synesthetes tend to be more consistent than controls on these measures. However, there has been no suggested diagnostic cut-off point for discriminating between the two samples.

Brang et al. (2010) tested 183 people and noted that as many as 44 % reported a possible spatial form for months of the year. However, when using a test of spatial consistency they classified only 2.2 % (4/183) as having a spatial form. To qualify as a synesthete in their study, a person had to not only report synesthesia but also to fall two standard deviations (SDs) away from the mean of the consistency scores of the normative sample. However, this diagnostic approach makes a strong assumption: namely, that synesthetes’ scores on this test should lie at the extreme tail end of the control distribution of scores. Whilst we would indeed expect synesthetes to be more consistent than controls, it is impossible to know, *a priori*, the magnitude of that difference.

The approach taken here is different insofar as we attempt to estimate the magnitude of the difference between self-reported synesthetes and controls in order to compute the optimal way of discriminating between them. This uses receiver operating characteristic (ROC) analyses of binary classifications to estimate the *sensitivity* (probability of classifying a self-reported synesthete as a synesthete) and *specificity* (probability of classifying a self-reported control as a control) of the measures. For instance, using this general approach we have shown that maximum sensitivity and specificity of tests used to diagnose grapheme-color synesthesia is 90 % and 94 %, respectively (Rothen, Seth, Witzel, & Ward, 2013).

## Methods

### Participants

Seventy participants were tested in total, among them 33 participants (24 female) who were classed as potential sequence-space synesthetes and 37 controls (27 female) who did not report this or any other kind synesthesia. All participants were between 18 and 65 years old with an average age of 23.1 years (SD = 7.1) for the controls and 28.2 years (SD = 12.0) for the potential synesthetes. The potential synesthetes were self-selected by filling in an online questionnaire that is linked to our research group (www.sussex.ac.uk/synaesthesia). A subset of the synesthetes also participated in an artificial grammar learning study (Rothen, Scott et al., 2013). The questionnaire was not advertised but can easily be found using online search engines by searching for “synaesthesia.”. Controls were recruited via notices at the University of Sussex and were mostly students of the University and were selected on the basis of being age and sex matched to the potential synesthetes. The controls were also given the synesthesia questionnaire, and any reporting SSS were excluded. The study was approved by the Sciences and Technology Cross-School Ethics Committee of the University of Sussex.

### Materials

Numbers (digits 0–9), days (N = 7), and month (N = 12) stimuli were presented on a screen (with resolution was set to 1024 × 768). The font was Courier New with a point size of 18 and in bold typeface. For days and months, the first letter was capitalized.

### Procedure

Our online screening questionnaire contains the question: “Some people always experience sequences in a particular spatial arrangement. Do you think this applies to you?” They were also shown a single example of a drawing of a three-dimensional (3D) spatial arrangement of days of the week as an example (Fig. 3b in Rothen, Meier, & Ward, 2012). Those giving an affirmative answer were then asked which kinds of stimuli they visualize in this way (with a choice from: Numbers, Days, Months, Years, Letters of the alphabet, Temperature, Height, Weight, Other – please specify). Those living locally were then invited to come to the University to participate in testing. Of those tested, 31 reported spatial forms for days, 33 for months, and 24 for numbers.

Participants were seated at a comfortable viewing distance to the screen. All participants were told to select a location for each of the presented stimuli by clicking with the mouse on a chosen position on the screen. Synesthetes were asked to use the screen as a reference frame for their spatial experiences and to arrange the stimuli as accurately as possible in the same way they are arranged in their synesthetic experience. If their synesthetic experience was arranged in a 3D, they were told to align their mental point of view so that they were able to transfer the arrangement into two dimensions. For stimuli that did not induce any synesthetic experiences, they were told to press the space bar, which led to the next stimulus without deciding a location. Controls were asked to find an intuitive location for the different stimuli. However, they were instructed to vary the location for different stimuli. Controls were not informed about the opportunity to press the space bar.

The words and digits were presented for 1 s, after which a central cross appeared and participants were required to make a mouse click in a location on the screen. All stimuli were presented three times in a random order making a total of 87 (=29 × 3) trials.

### Analysis

The placement of the three coordinates for each item can be conceptualized as a two-dimensional (2D) triangle. From this, three different measures of consistency were calculated: the perimeter of the triangle (i.e., the sum of Euclidean distances between each point); the maximum length of the sides of the triangle; and the area of the triangle. The first two measures are in pixels and the third in pixel-squared. Items for which one or more trial was omitted (because the synethete pressed the space bar) were excluded from the analysis. It is suggested that other researchers should either adopt the same monitor resolution or apply a linear transformation of the data when using other resolutions. A final measure used a “nearest neighbor” method in which each trial in a given set (e.g., days of the week) is compared to all the other trials to determine what the nearest other value is based on Euclidean distances (this approach is based on Brang et al., 2010). If a trial relating to, say, “Tuesday” has a nearest neighbor that is another “Tuesday” trial then this counts as a hit otherwise it counts as a miss. The final nearest neighbor measure is the percentage of hits across all trials and sets.

We applied ROC curve analysis to binary classification of self-declared synesthetes and non-synesthete controls to determine, for each measure of consistency, the cut-off value maximizing sensitivity and specificity for the given samples. Sensitivity and specificity rates were calculated for all unique consistency values, allowing identification of an optimal cut-off value: the point with the highest true positive rate and lowest false positive rate. The method enables a quantitative comparison of the discriminatory performance of different ways of calculating consistency. Discriminatory performance in each condition can be expressed as a single value, referred to as Discriminant Power (DP; also known as test effectiveness) associated with the corresponding optimal cut-off for that condition, and which can be interpreted as the standardized distance between the means of two populations. DP values around 1 are regarded as not effective in discriminating between two samples. As an additional measure, the more commonly used area under the curve (AUC) is also provided. AUC is the probability of a given consistency measure, and its corresponding cut-off, correctly classifying a randomly drawn pair of a synesthete and a control.

## Results

The results are summarized in Table 1. The potential synesthete group performed significantly more consistently than the controls irrespective of whether consistency was measured as the sum of Euclidean distances (i.e., perimeter, t(43.5) = 4.28, p < .001), the maximum length (t(44.4) = 4.27, p < .001), the area (t(37.2) = 3.18, p = .003), or the nearest neighbor method (t(68) = 4.88, p < .001). For the first three t-tests, there was inhomogeneity of variance (Levene’s test was significant) and the degrees of freedom was adjusted accordingly. In terms of the discriminant power (DP) of the various measures, the area-based measurement of consistency performs the best. This gives a sensitivity of 88 % and a specificity of 70 %.

**Table 1:** Summary statistics for the three different measures of consistency: either including all participants (top table) or excluding participants who place all their responses in the central horizontal band (300<y<500) or click on the same region of space for a given inducer (e.g., all days clicked on top right) generating high consistency but no sequence (bottom table)

Descriptive	DP	AUC	Mean (syn)	Mean (con)	SD (syn)	SD (con)	Sensitivity	Specificity	Cut-off	N syn / con
Optimal binary classification of all participants										
Area	1.57	0.76	1079	7031	1365	11149	88	70	1,596	33 / 37
Max. length	1.20	0.77	96	194	42	130	79	70	110	33 / 37
Perimeter (Euclidean sum)	1.18	0.77	202	415	87	284	76	73	221	33 / 37
Nearest neighbor	0.93	0.76	66	42	21	22	67	73	56	33 / 37
Optimal binary classification after removal by visual inspection										
Area	1.84	0.85	1164	8085	1403	11641	87	81	1,596	30 / 32
Perimeter (Euclidean sum)	1.46	0.82	207	453	90	287	77	81	236	30 / 32
Max. length	1.46	0.82	98	211	44	132	77	81	110	30 / 32
Nearest neighbor	1.08	0.79	66	40	21	22	67	78	55	30 / 32

*DP* discriminant power, *AUC* area under the curve, *SD* standard deviation, *Max.* maximum

**Fig. 1:**
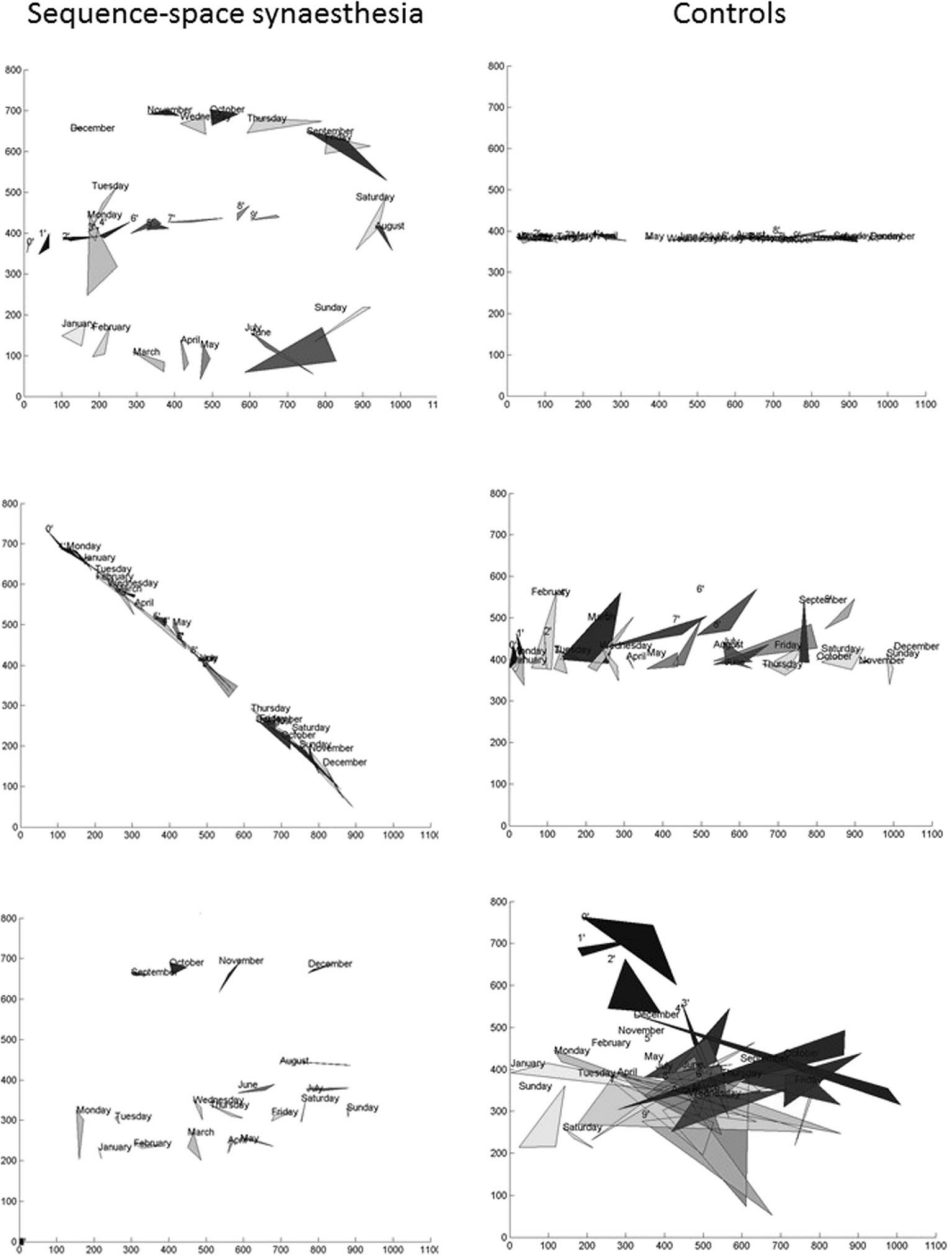
Visual depictions of spatial forms generated by potential synesthetes (left) and controls (right). The axis scales represent pixels but were not visible to participants during the study. The triangles represent the area bounded by the three different x, y coordinates chosen for each item (the shading is irrelevant). The consistency scores, calculated as area, for the three synesthetes (top to bottom) were 1,553, 501, and 322; for the three controls the scores were 168, 1,807, and 9,719

Examples of some datasets from synesthetes and controls are shown in Fig. 1 and the full responses are included as Supplementary Material (together with a MatLab script for generating visualizations of the spatial forms). There was a greater tendency for synesthetes to use non-linear sequential arrangements than controls. For instance, 42 % of synesthetes showed an elliptical calendar form compared to 5 % of controls (χ^2^(1) = 13.56, p < .001). Some participants (N = 3 potential synesthetes, N = 4 controls) always placed their responses around the central horizontal band of the screen (between 300 and 500 pixels), bearing in mind that the centre (y = 384 pixels) had been marked at the start of each trial by the fixation cross and stimulus. This strategy, if indeed that is what it was, tended to yield a high consistency score. One further control participant achieved high consistency (on most of our measures) by placing inducers of a particular kind in the same spatial position (e.g., all days placed at top right) thus not resembling a sequence. The nearest neighbour method is, however, able to correctly categorize this participant. If one excludes these N = 8 participants, then the discriminant power across all three measures of consistency goes up. The best performing consistency measure remains the area of the triangle giving a sensitivity and specificity of 87 % and 81 %, respectively.

## Discussion

Despite sequence-space synesthesia being widely believed to be the most prevalent form of synesthesia, there have been no agreed upon standards in the literature for its measurement. The aim of this study was to build on previous methodology (e.g., Brang et al., 2010; Eagleman, 2009; Smilek et al., 2006) in order to develop a test that is easy to administer and to find the optimal method for discriminating between the groups. To this end, we collected data from a group of potential sequence space synesthetes and a group of controls who claim not to have synesthesia. A spatial location on the computer screen had to be chosen on three occasions for each stimulus (e.g., “Monday”) and the average area of space bounded by these coordinates (i.e., a triangle) offered the most efficient way of discriminating between the groups. Discrimination was further enhanced by excluding a small number of participants by visual inspection of certain strategies that afford high consistency (placing all inducers horizontally on the centre of the screen). It is only after the development of this method and cut-off that future research can approach the question of prevalence with real confidence.

The results suggest that this is a useful diagnostic tool: around nine out of ten sequence space synesthetes can be diagnosed with this method. This performance is impressive given that synesthetes have to remap their spatial form either from an “inner screen” or from peri-personal space on to the space defined by the computer monitor. Many of the synesthetes would also have to remap from 3D to 2D. Although further studies are needed with larger samples to consider these spatial sub-types, our results suggest that the present test is relatively robust to these variations in phenomenology. However, the *specificity* of this method is considerably lower than the equivalent best color-based consistency measure (Rothen, Seth, et al., 2013). Controls find the task of placing stimuli representing sequences in space to be far more intuitive, or amenable to successful strategies, than choosing colors for graphemes. This is consistent with the wider literature on implicit number-space (Fias & Fischer, 2005) and time-space (Santiago, Lupáñez, Pérez, & Funes, 2007) associations. The method that we have proposed is regarded as a good measure for naïve participants but we do not regard it to be resilient to deliberate attempts to learn or practice sequence-space associations prior to taking the test (as applies to most other behavioral markers of synesthesia). One possibility for improving specificity is to measure consistency across sessions (e.g., Brang et al. 2010).

Of course, the measures of sensitivity and specificity are predicated on us having a reasonable initial classification of the two groups to begin with. It may well be that some of our “controls” have SSS, and that some of our “synesthetes” misreported this. If that is the case then the figures reported here represent lower-bound estimates. The first-person report of SSS is likely to remain an essential feature in the literature and the development of tools to assess this aspect (e.g., via questionnaire) is important.

**Fig. 2:**
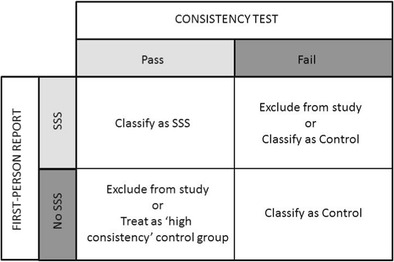
The suggested method for classifying participants based on their scores on our test of consistency and first-person report of experiencing sequence-space synesthesia (SSS)

Figure 2 shows the suggested classification scheme and analysis strategy based on the current diagnostic test plus the presence/absence of self-reported experiences of sequence-space synesthesia. The suggestion here is that future researchers should diagnose the presence of SSS by the combination of self-report plus an average area-based consistency score of less than 1,596 squared pixels (or 0.2029 % of the total area on monitors with other resolutions). Assessing the *absence* of SSS, where it is important to do so, could be done in several ways. A conservative and simple criteria would be to classify all participants with a consistency score greater than 1,596 pixel-squared as controls. A small number of SSS may be misclassified as controls but this is unlikely to skew the data given that SSS is rarer than the neurotypical profile (Sagiv et al., 2006). The most problematic group are people who behave like the SSS group on the consistency measure but claim to not experience this. Two possibilities for this group are either outright exclusion (given that they are a minority of participants) or to treat them as a separate group in the analysis (as “highly consistent controls”).

In summary, this type of synesthesia has intrigued scientists for over 130 years but the lack of an agreed-upon tool for assessing it has held back research in this phenomenon (compared to other types of synesthesia where such tests exist). This study presents a simple methodology with good sensitivity and reasonable specificity, together with normative values that can be used by other researchers in the field.

## Electronic supplementary material

Below is the link to the electronic supplementary material.

ESM 1 (PDF 1768 kb)

ESM 2 (PDF 2641 kb)
