# Supplementary material for: The sensitivity and specificity of a diagnostic test of sequence-space synesthesia
Source: Behav Res Methods. 2015 Nov 11;48(4):1476–81. doi: 10.3758/s13428-015-0656-2 (PMC5101286; doi:10.3758/s13428-015-0656-2)
Supplement: Supplementary file 1 — (PDF 1768 kb) [file 13428_2015_656_MOESM1_ESM.pdf]

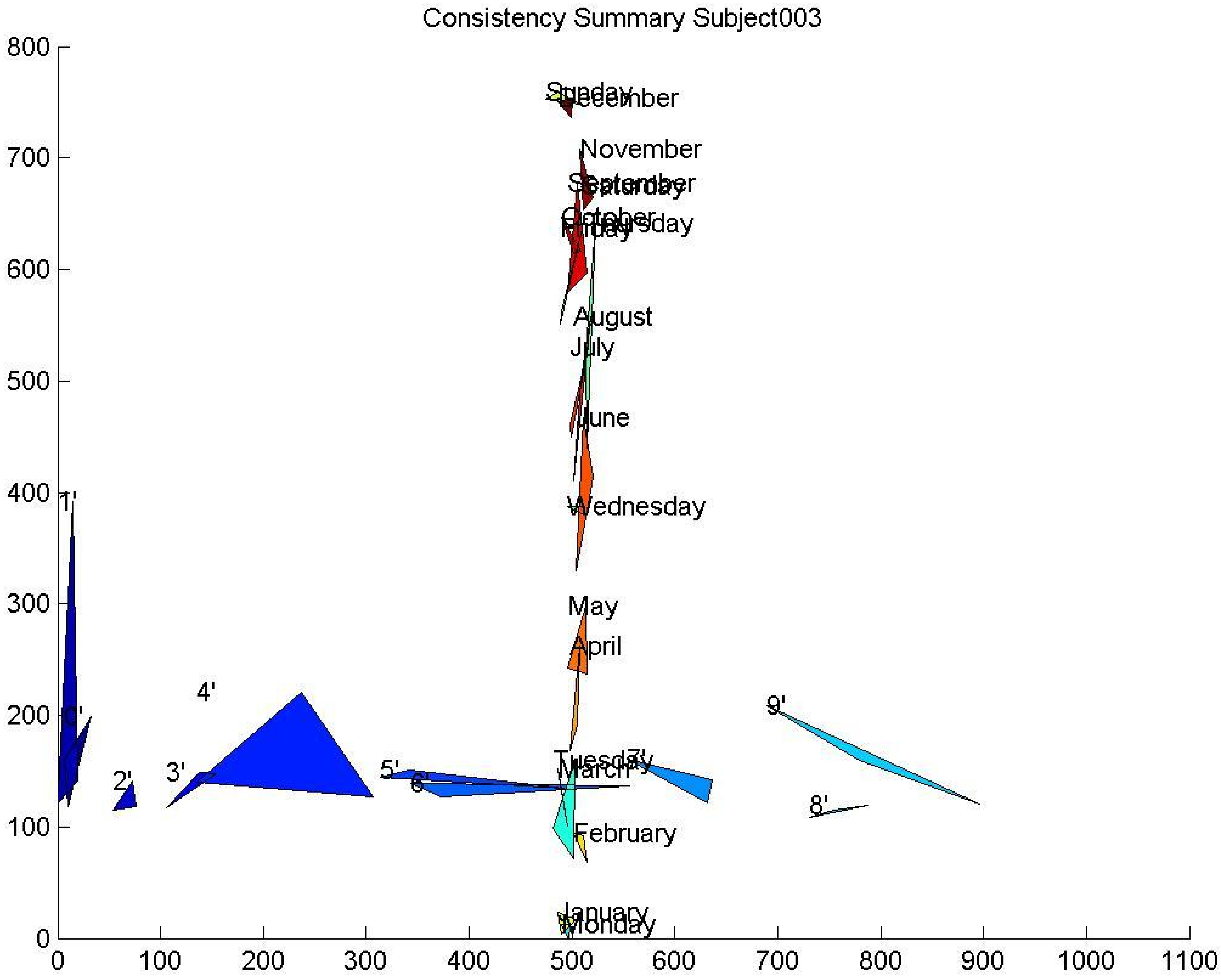
Consistency Summary Subject003
800
700
600
500
400
300
200
100
0
0
100
200
300
400
500
600
700
800
900
1000
1100
November
August
July
June
Wednesday
May
April
February
1'
2'
3'
4'
5'
8'
9'

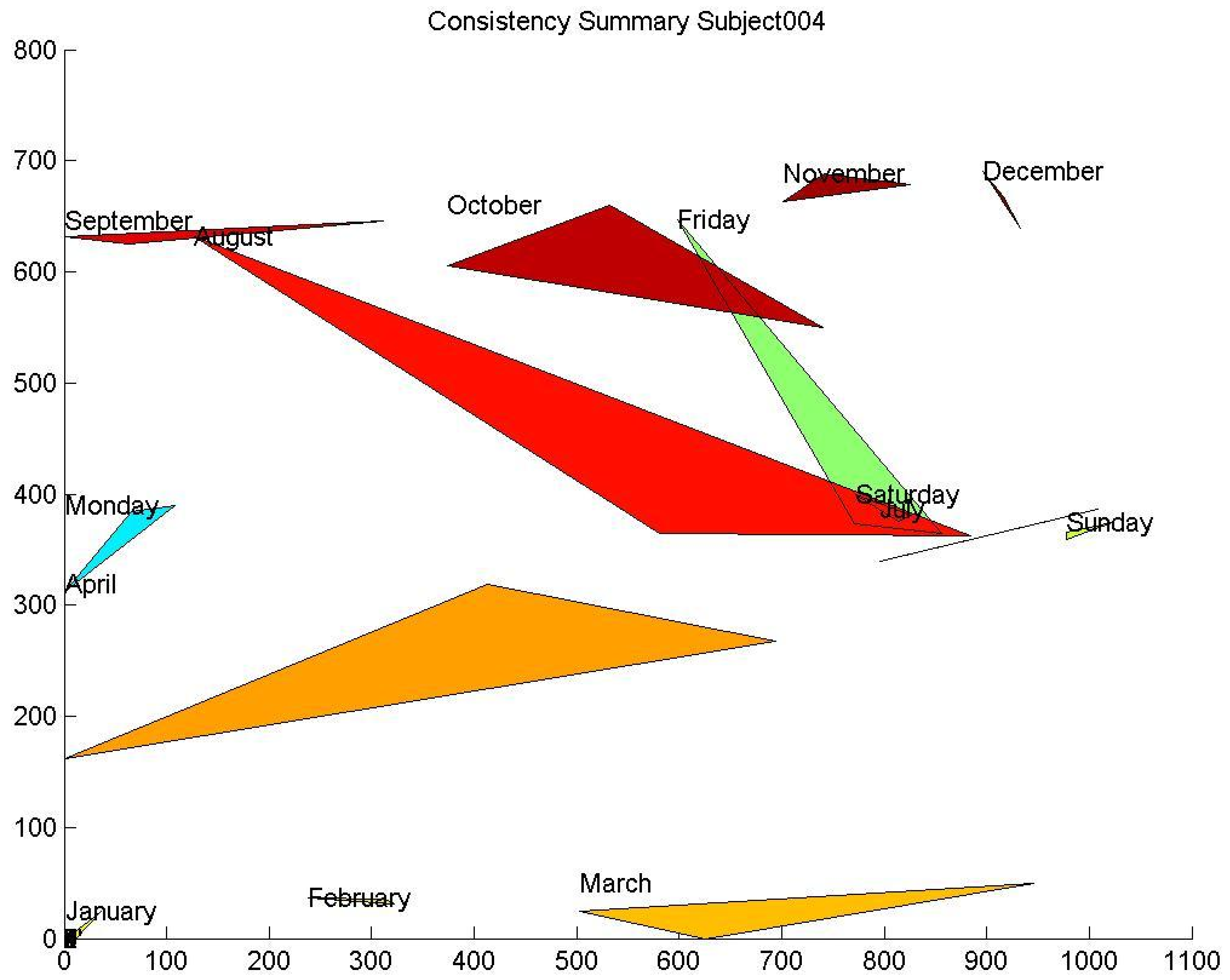

Consistency Summary Subject004
September
August
October
November
December
Friday
Monday
April
Saturday
July
Sunday
March
February
January
800
700
600
500
400
300
200
100
0
0
100
200
300
400
500
600
700
800
900
1000
1100

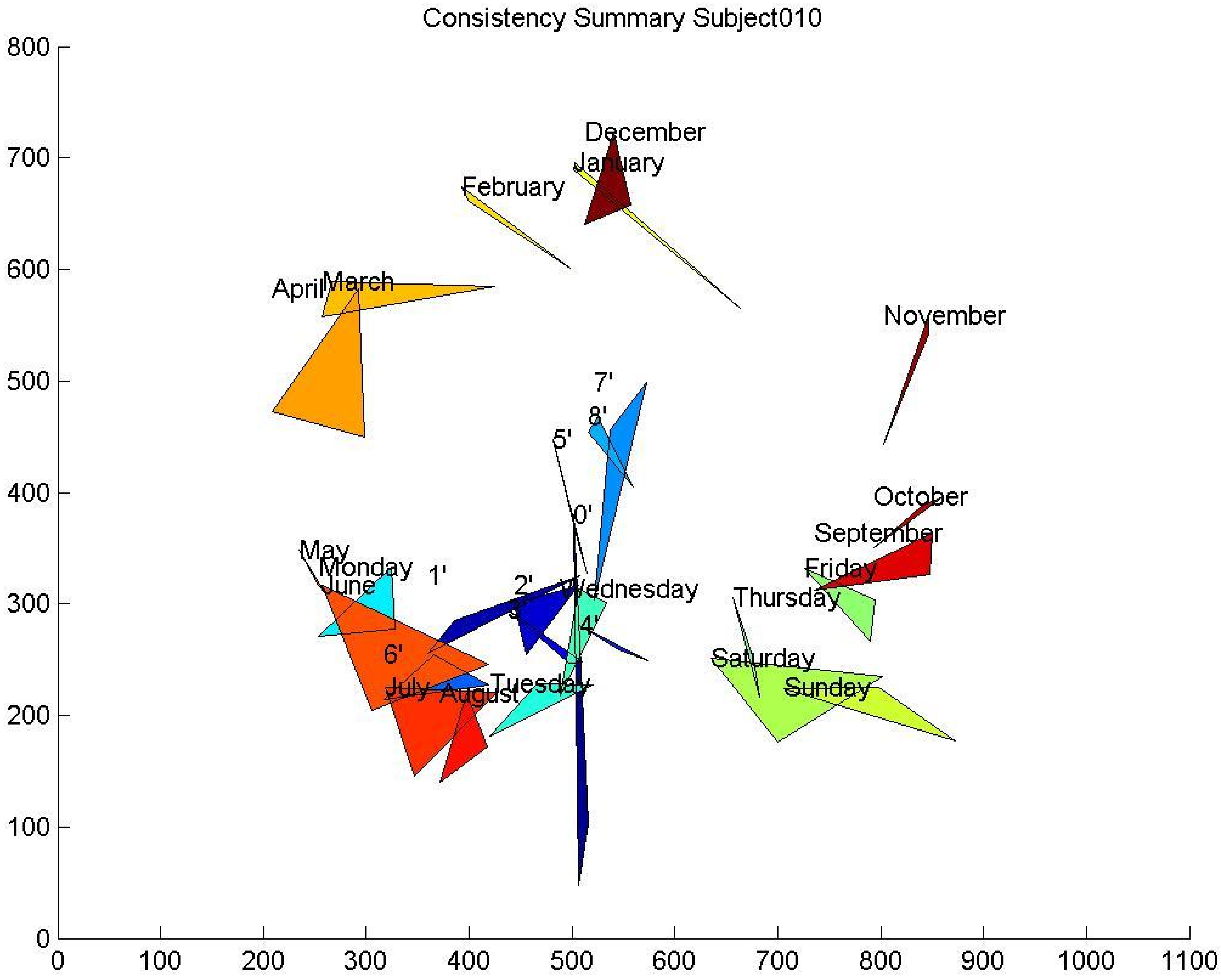
Consistency Summary Subject010
December
January
February
March
April
November
7'
8'
5'
0'
October
September
May
Monday
June
1'
2'
3'
Wednesday
Friday
Thursday
4'
6'
Saturday
July
August
Tuesday
Sunday
0
100
200
300
400
500
600
700
800
900
1000
1100

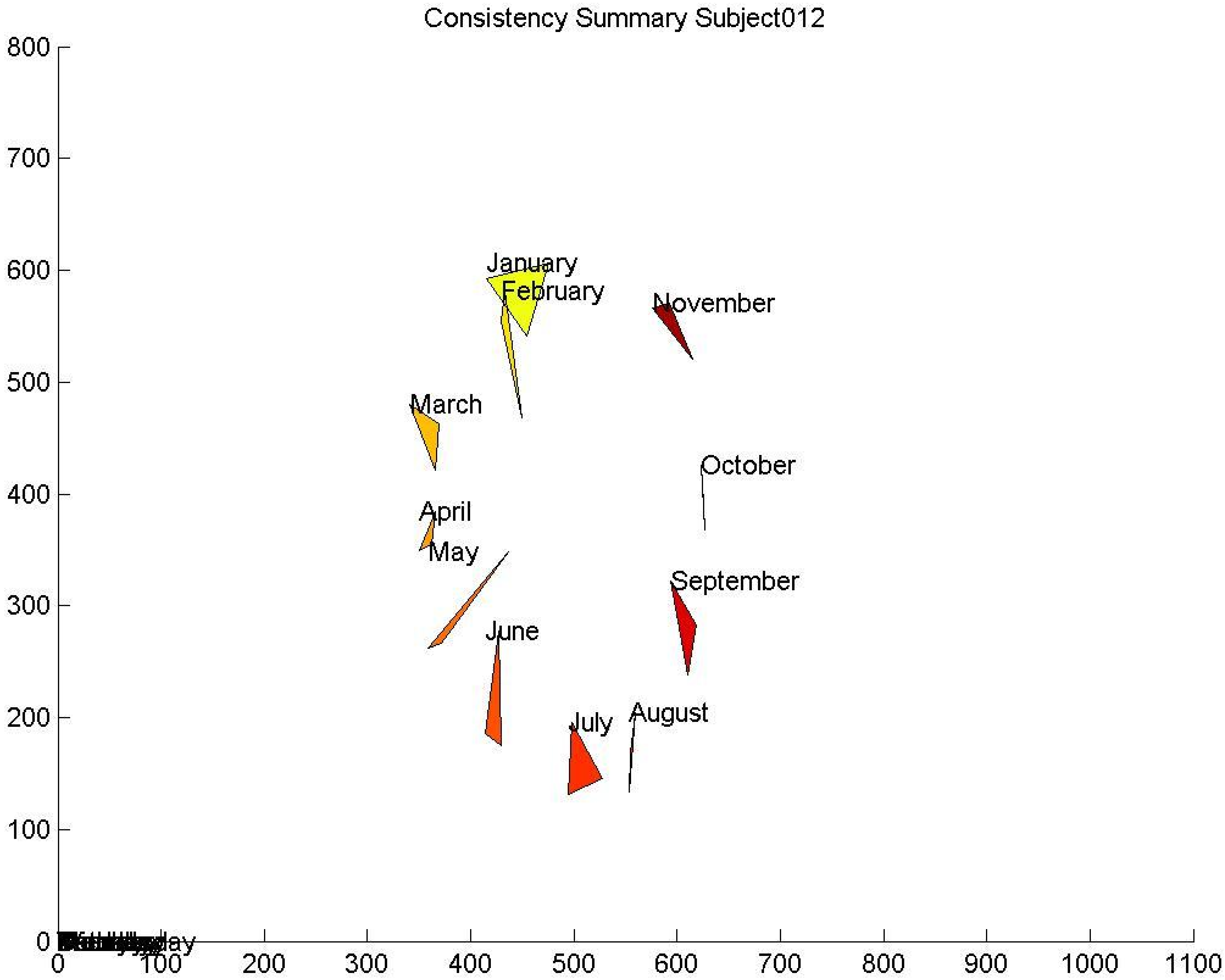
Consistency Summary Subject012
January
February
November
March
October
April
May
September
June
July
August
800
700
600
500
400
300
200
100
0
100
200
300
400
500
600
700
800
900
1000
1100

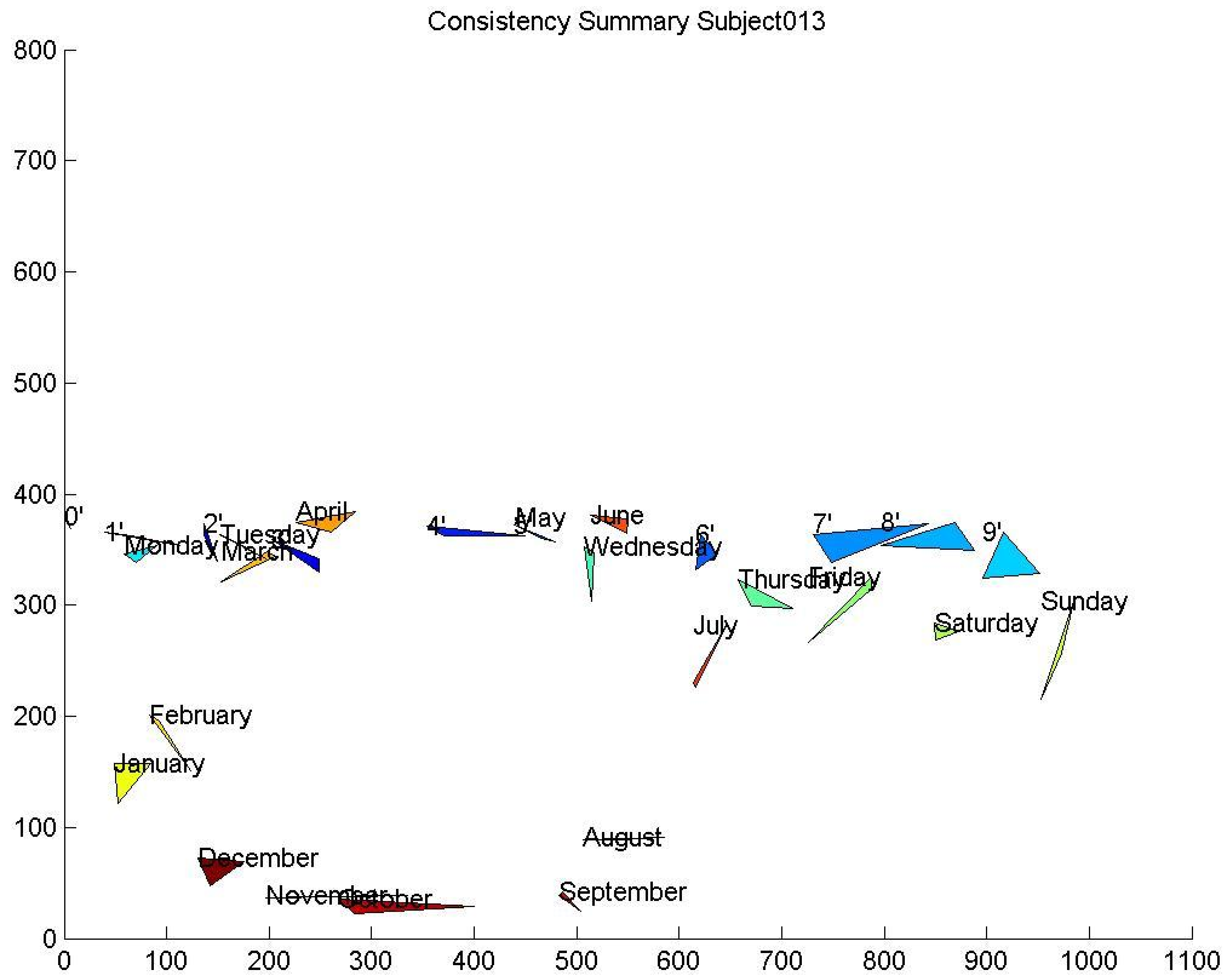

Consistency Summary Subject013
800
700
600
500
400
300
200
100
0
0
100
200
300
400
500
600
700
800
900
1000
1100
0'
1'
2'
4'
5'
6'
7'
8'
9'
Monday
Tuesday
Wednesday
Thursday
Friday
Saturday
Sunday
January
February
March
April
May
June
July
August
September
October
November
December

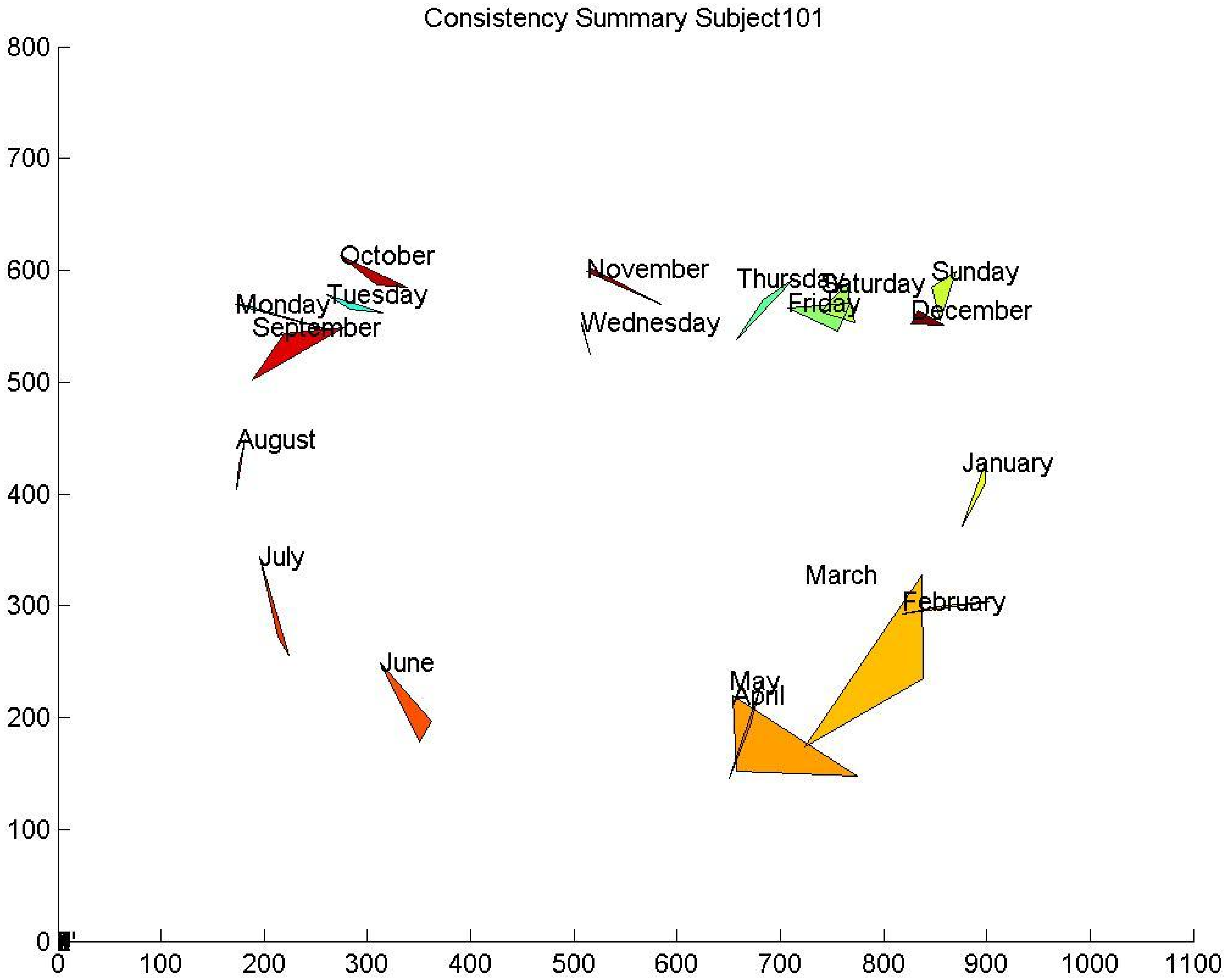

Consistency Summary Subject101
October
Tuesday
Monday
September
November
Wednesday
Thursday
Saturday
Friday
Sunday
December
August
January
July
March
February
June
May
April
0
100
200
300
400
500
600
700
800
0
100
200
300
400
500
600
700
800
900
1000
1100

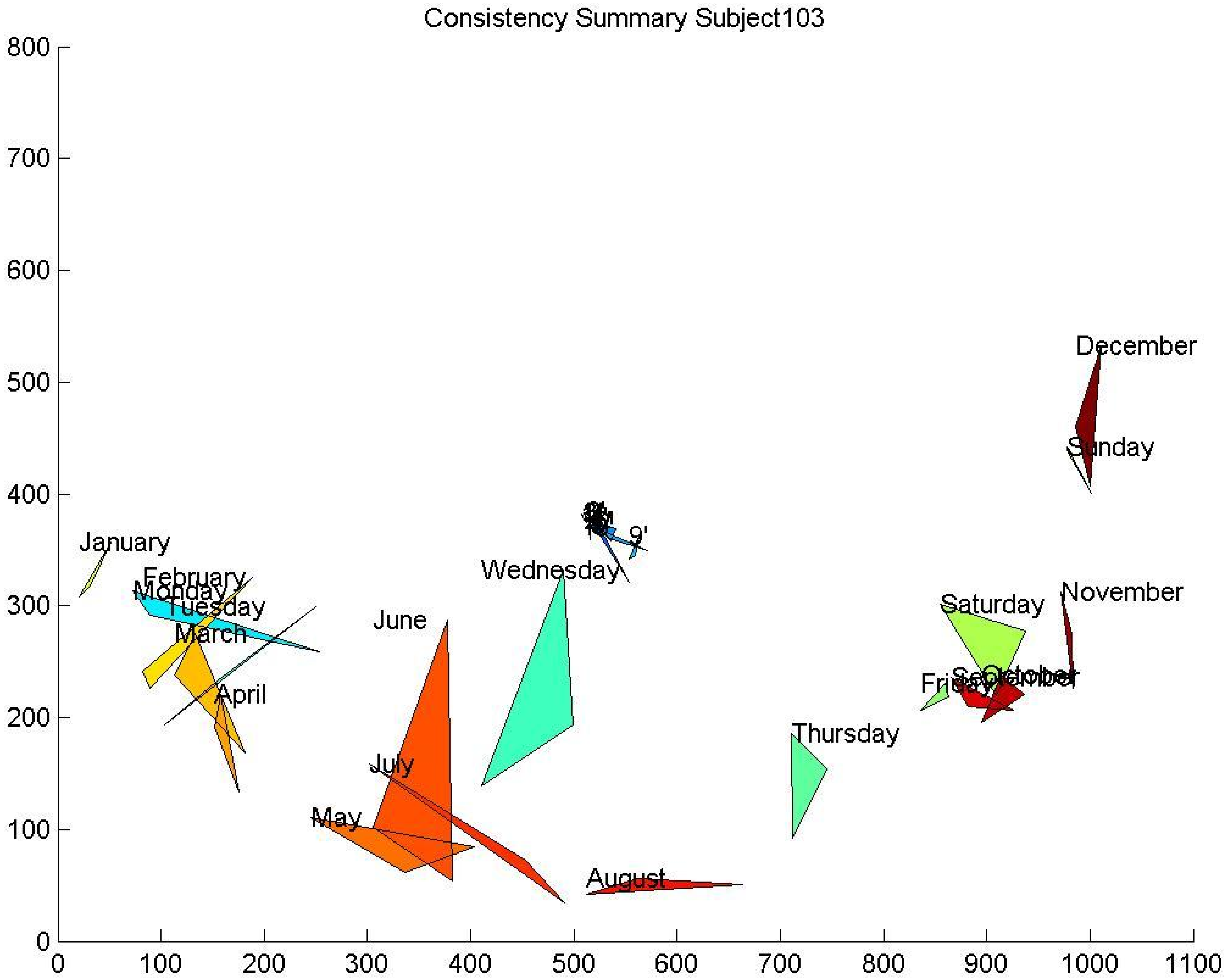
Consistency Summary Subject103
800
700
600
500
400
300
200
100
0
0
100
200
300
400
500
600
700
800
900
1000
1100
December
Sunday
January
February
Monday
Tuesday
March
April
June
July
May
August
Wednesday
Thursday
Saturday
November
Friday
October

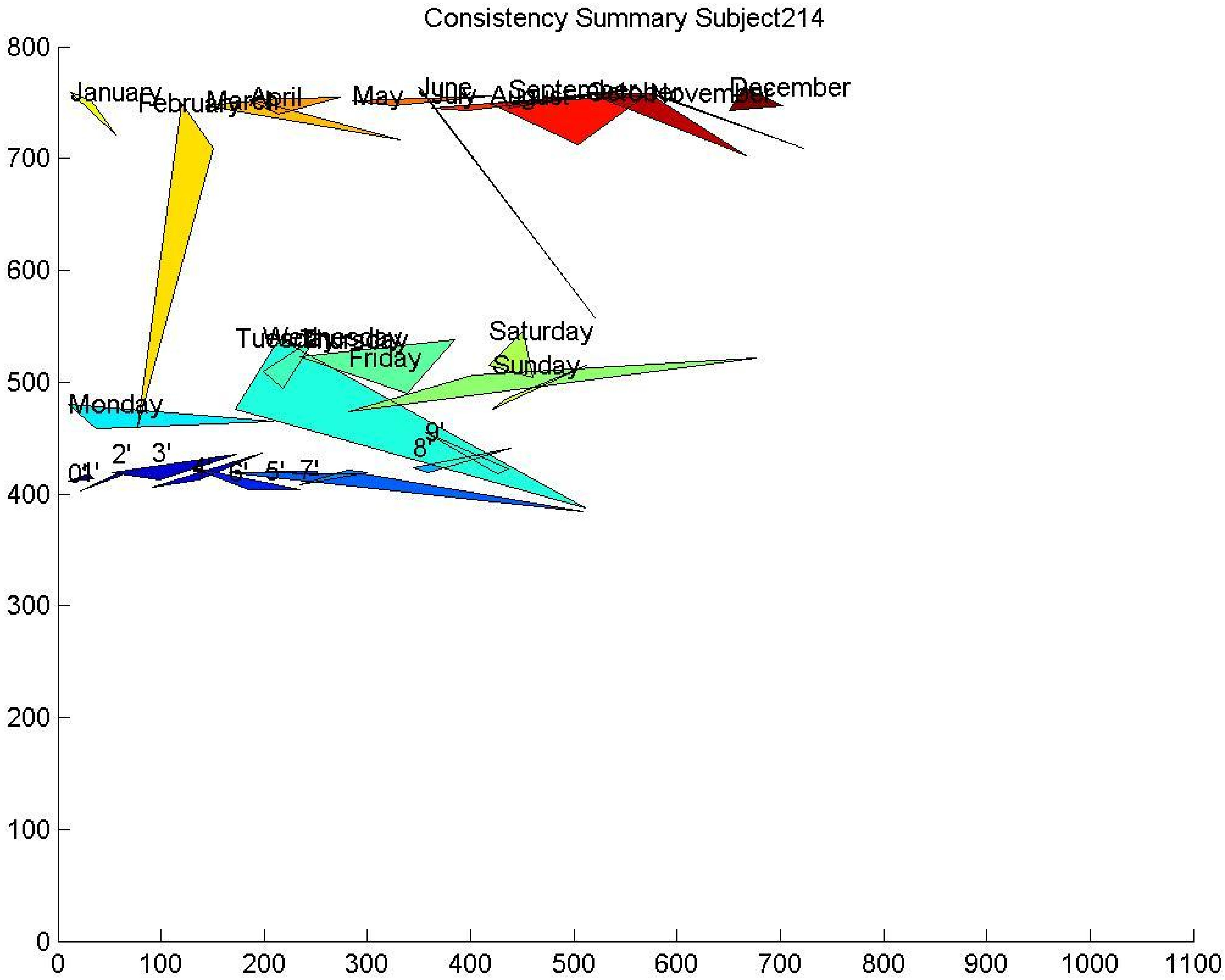
Consistency Summary Subject214
January
February
April
May
June
December
Saturday
Sunday
Friday
Monday
2'
3'
8'
9'
0
100
200
300
400
500
600
700
800
900
1000
1100

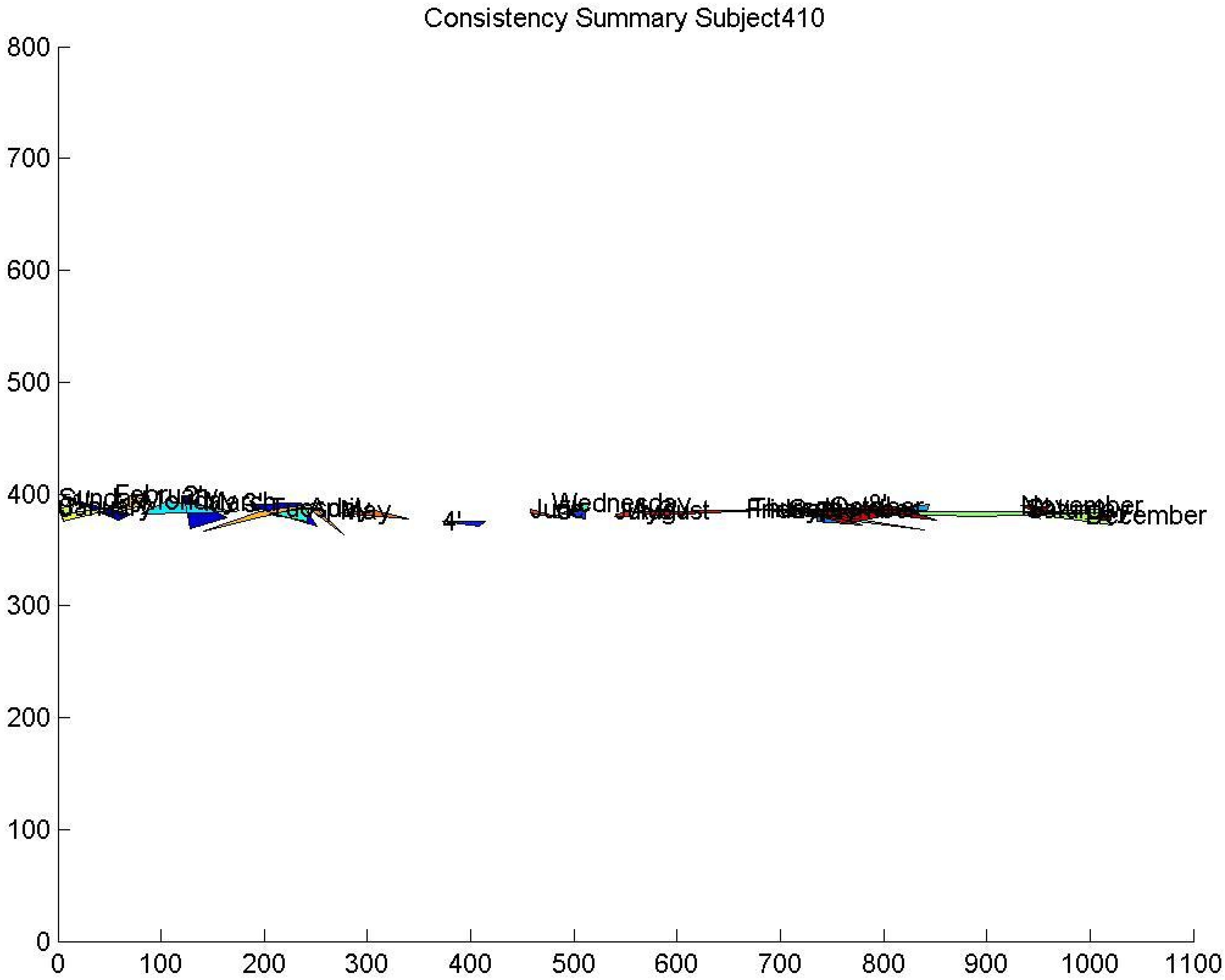
Consistency Summary Subject410
800
700
600
500
400
300
200
100
0
0
100
200
300
400
500
600
700
800
900
1000
1100
Wednesday
August
December
May

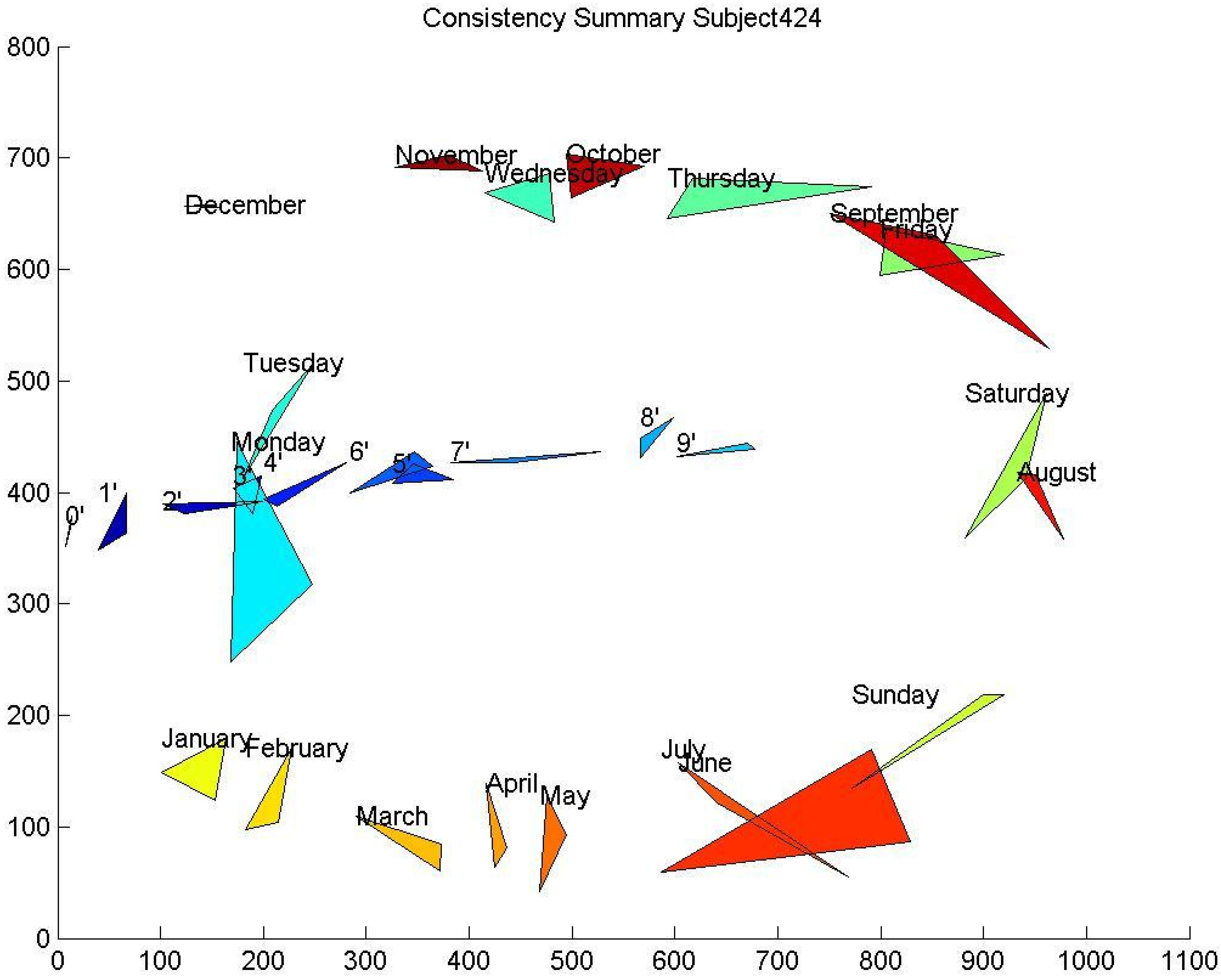

Consistency Summary Subject424
November
October
Wednesday
Thursday
December
September
Friday
Tuesday
Saturday
8'
Monday
9'
6'
7'
4'
5'
3'
August
1'
2'
0'
Sunday
January
February
July
June
April
May
March
0
100
200
300
400
500
600
700
800
900
1000
1100

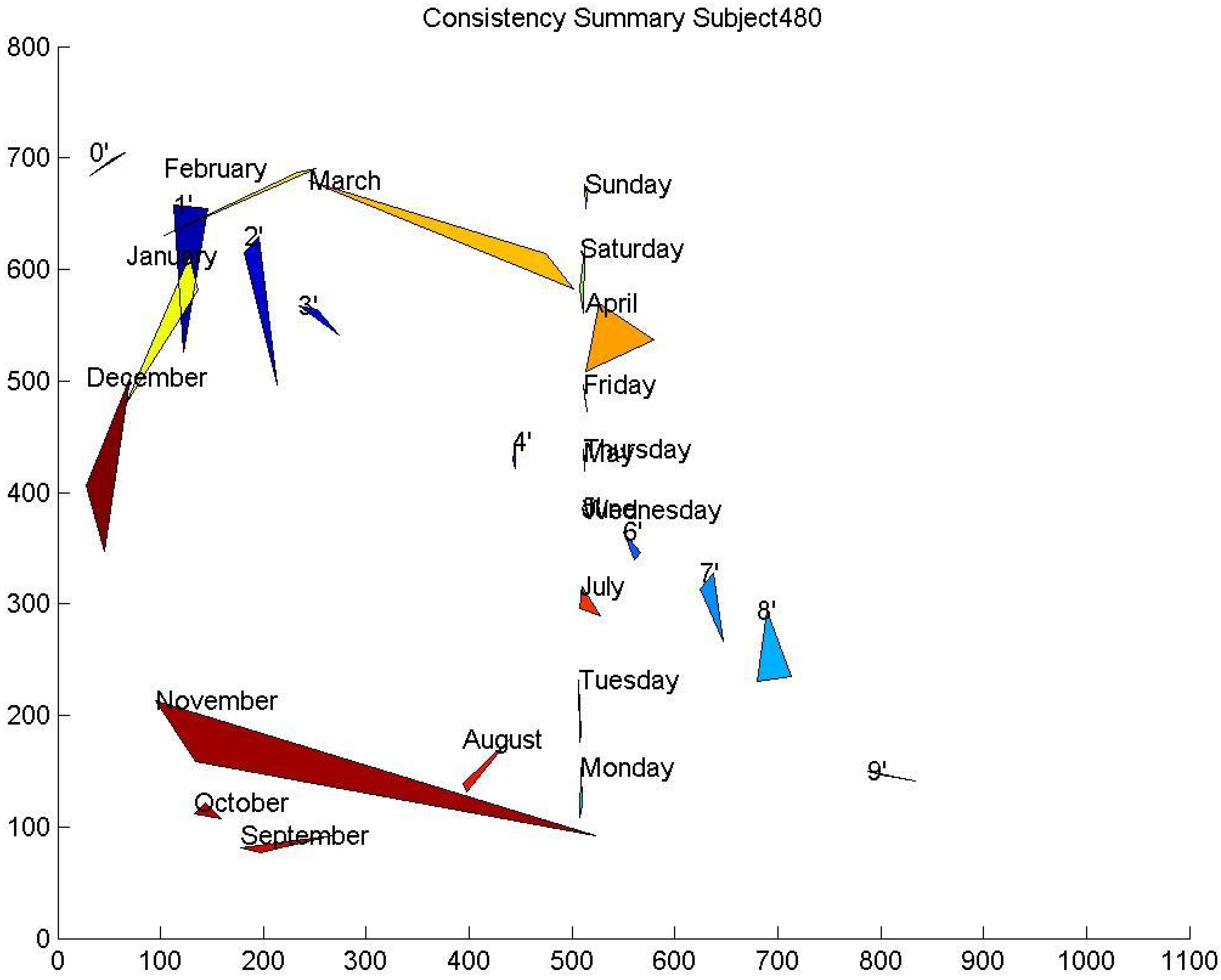
Consistency Summary Subject480
0'
1'
2'
3'
4'
6'
7'
8'
9'
January
February
March
April
May
June
July
August
September
October
November
December
Sunday
Monday
Tuesday
Wednesday
Thursday
Friday
Saturday
0
100
200
300
400
500
600
700
800
900
1000
1100

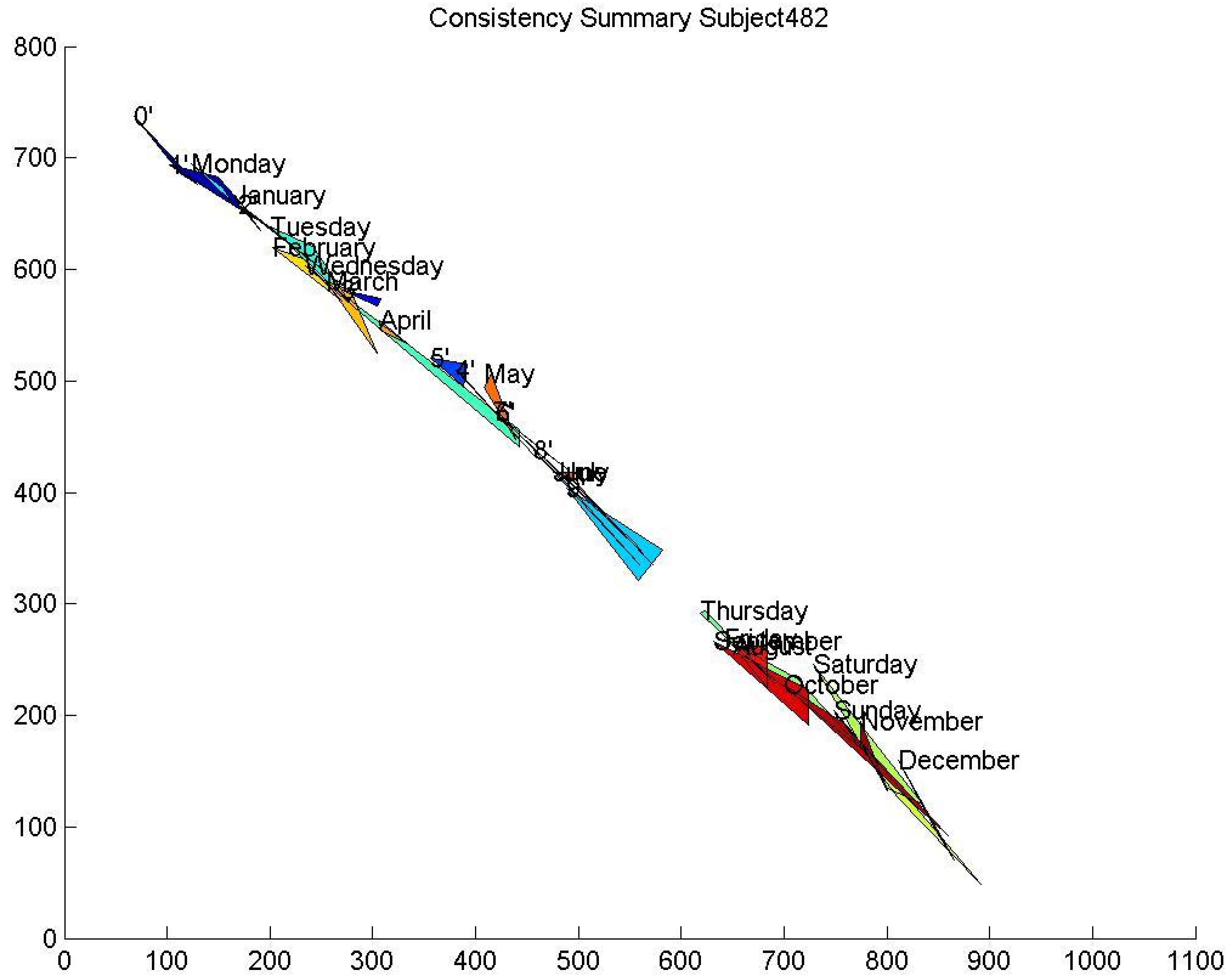
Consistency Summary Subject482
0'
Monday
January
Tuesday
February
Wednesday
March
April
5'
4'
May
8'
Thursday
Saturday
October
Sunday
November
December
0
100
200
300
400
500
600
700
800
900
1000
1100

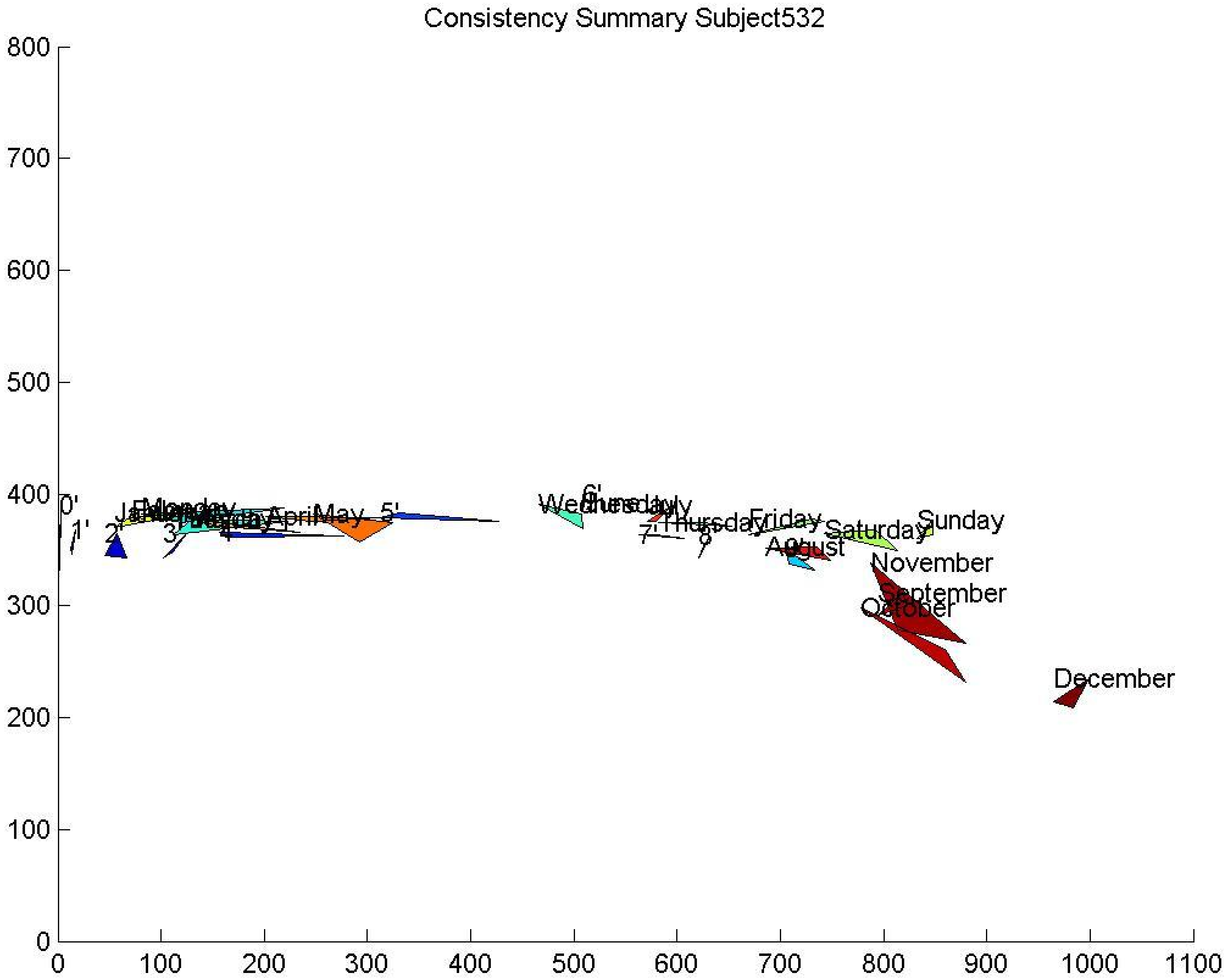
Consistency Summary Subject532
800
700
600
500
400
300
200
100
0
0
100
200
300
400
500
600
700
800
900
1000
1100
0'
1'
2'
3
5'
May
Wednesday
Thursday
Friday
Saturday
Sunday
August
November
September
October
December

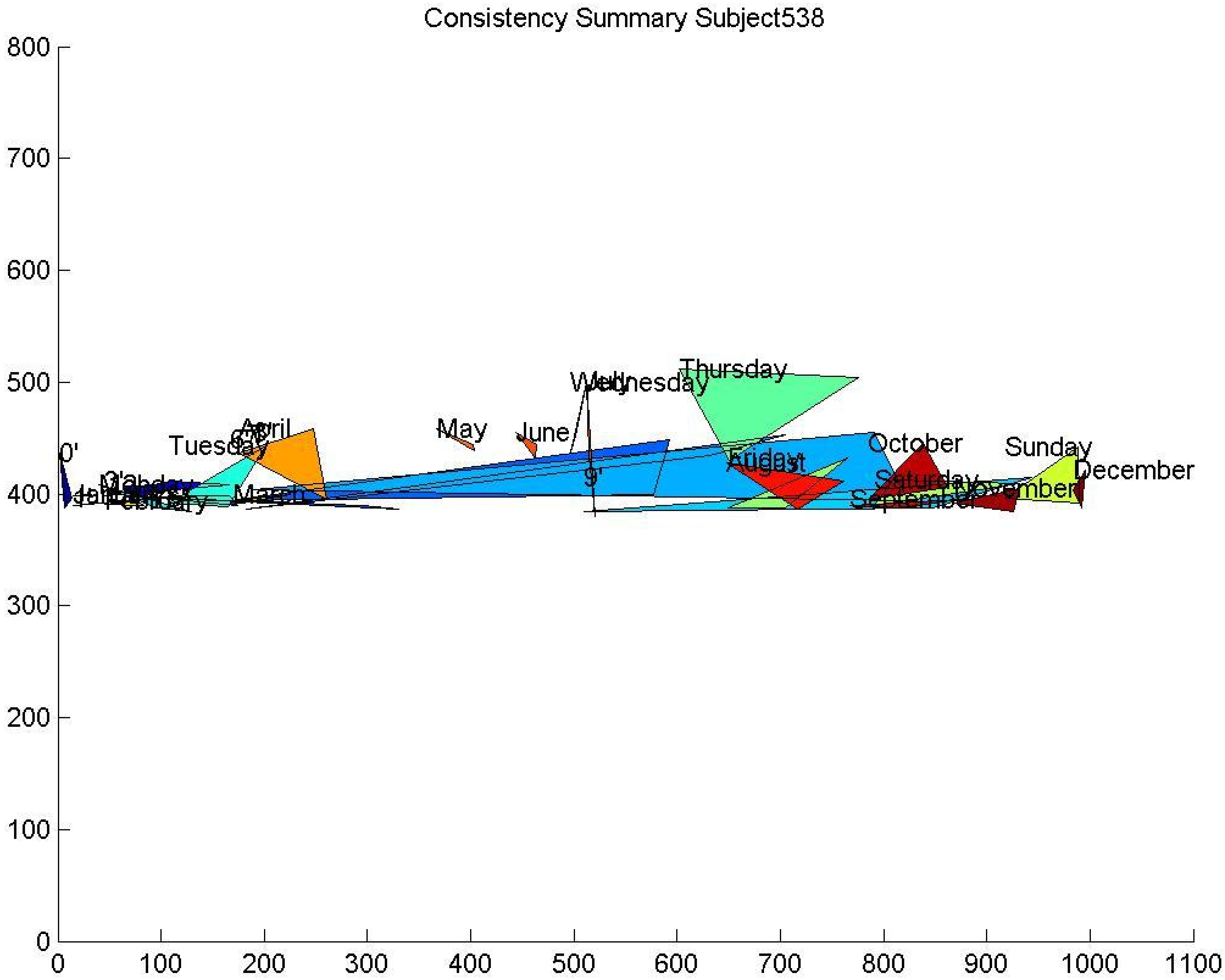
Consistency Summary Subject538
800
700
600
500
400
300
200
100
0
100
200
300
400
500
600
700
800
900
1000
1100
Thursday
Wednesday
April
Tuesday
May
June
October
Sunday
December
Friday
August
Saturday
November
September
March
0'
9'

Consistency Summary Subject593

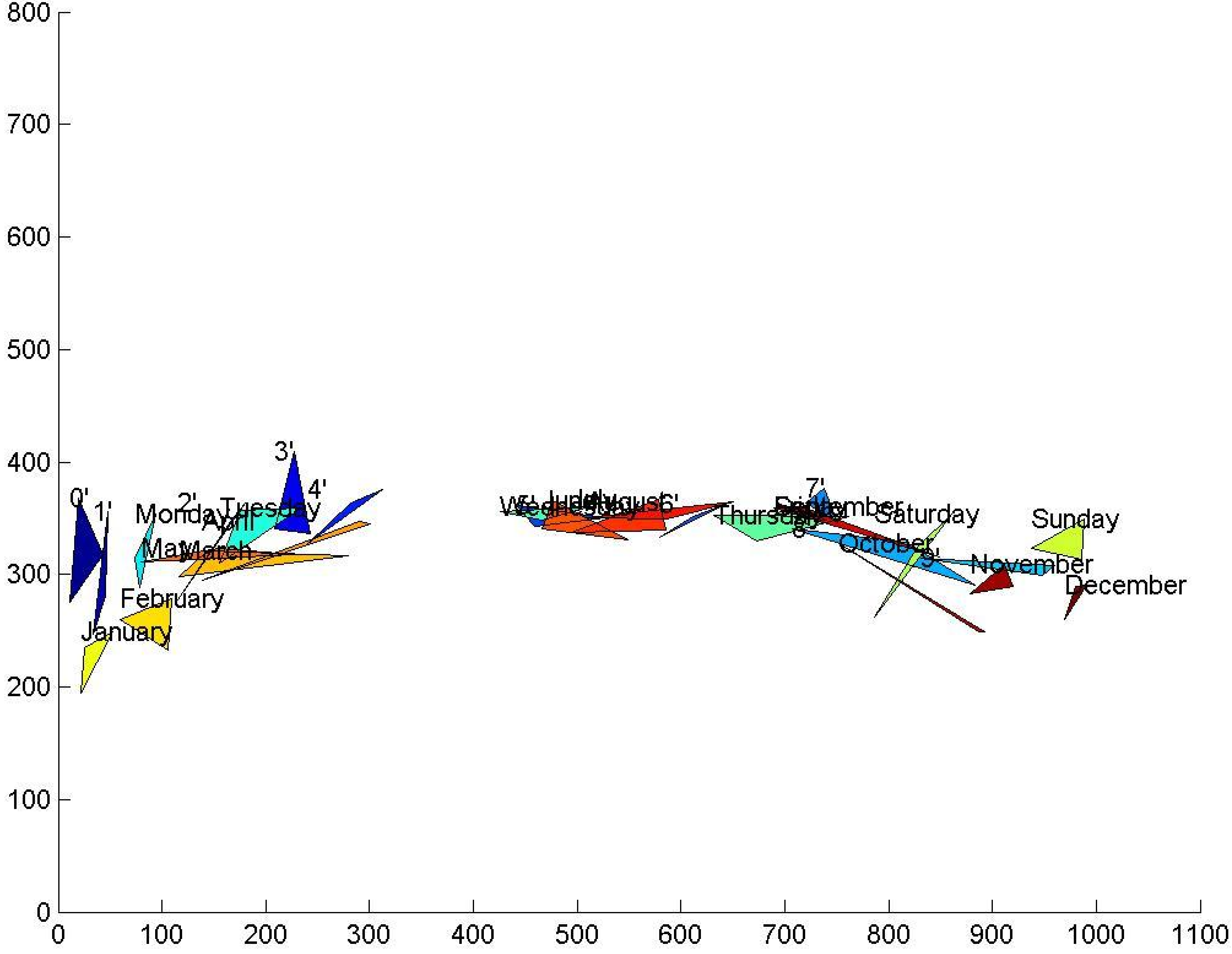

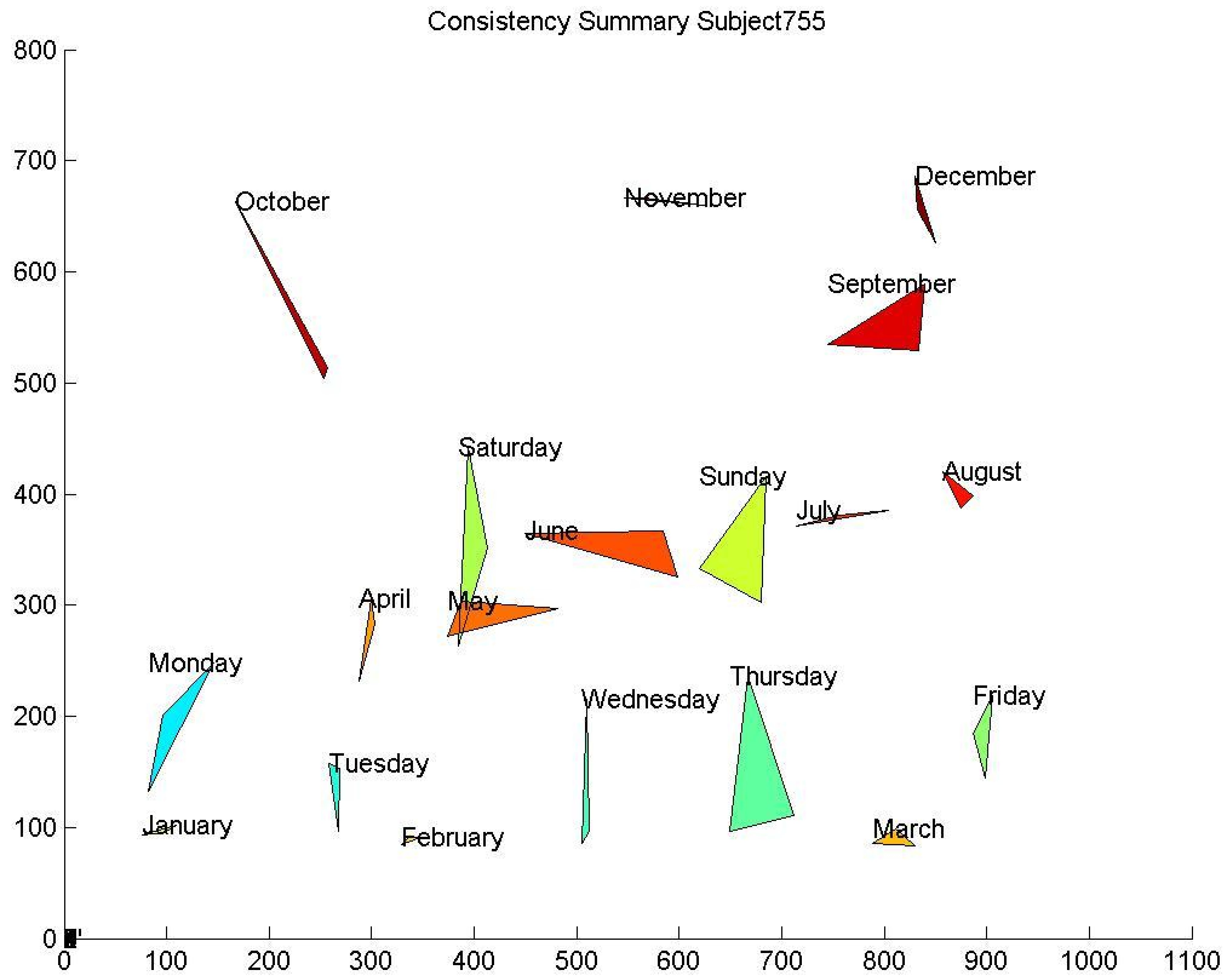
Consistency Summary Subject755
October
November
December
September
Saturday
Sunday
July
August
June
April
May
Monday
Thursday
Wednesday
Friday
Tuesday
January
February
March
800
700
600
500
400
300
200
100
0
0
100
200
300
400
500
600
700
800
900
1000
1100

Consistency Summary Subject792

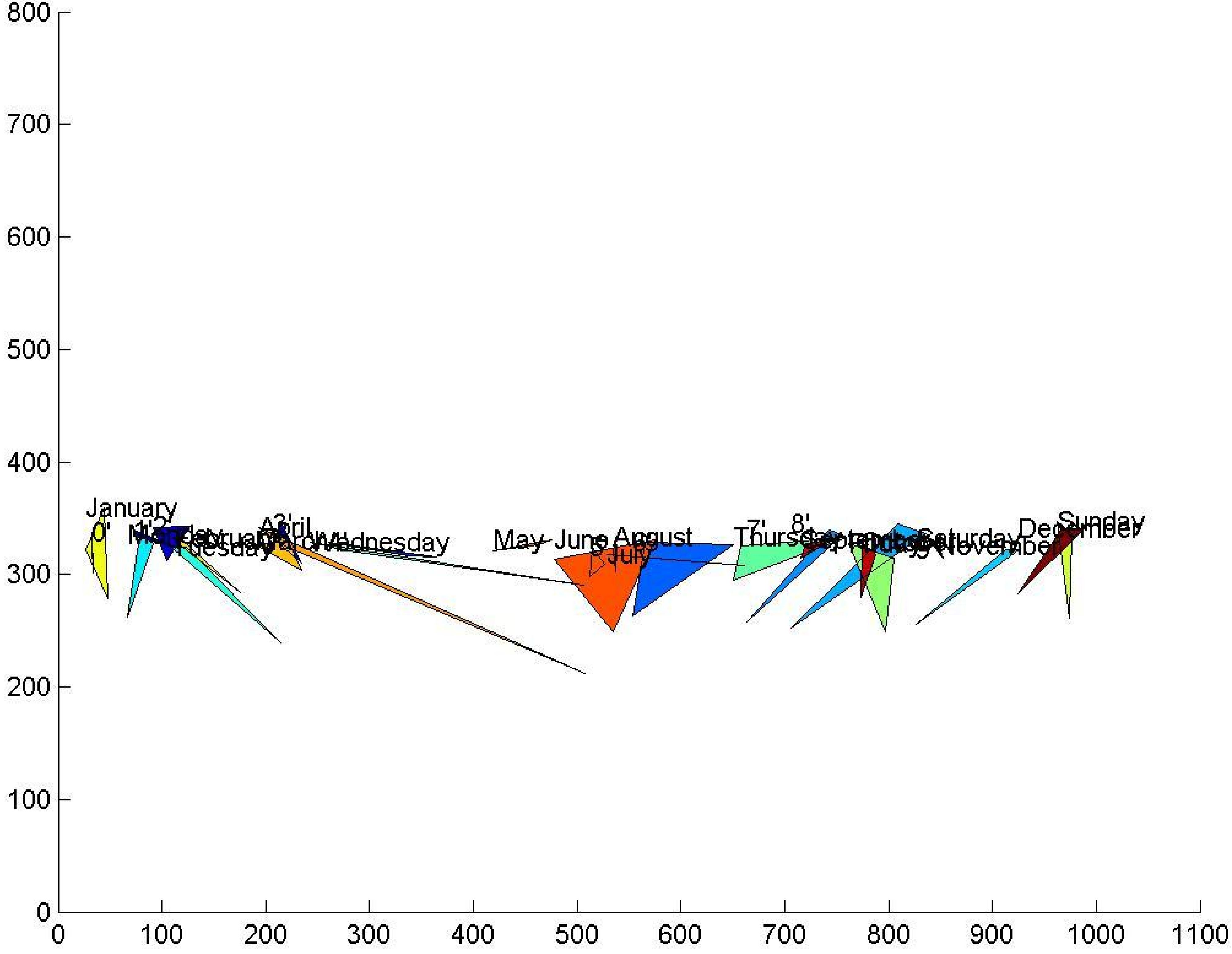

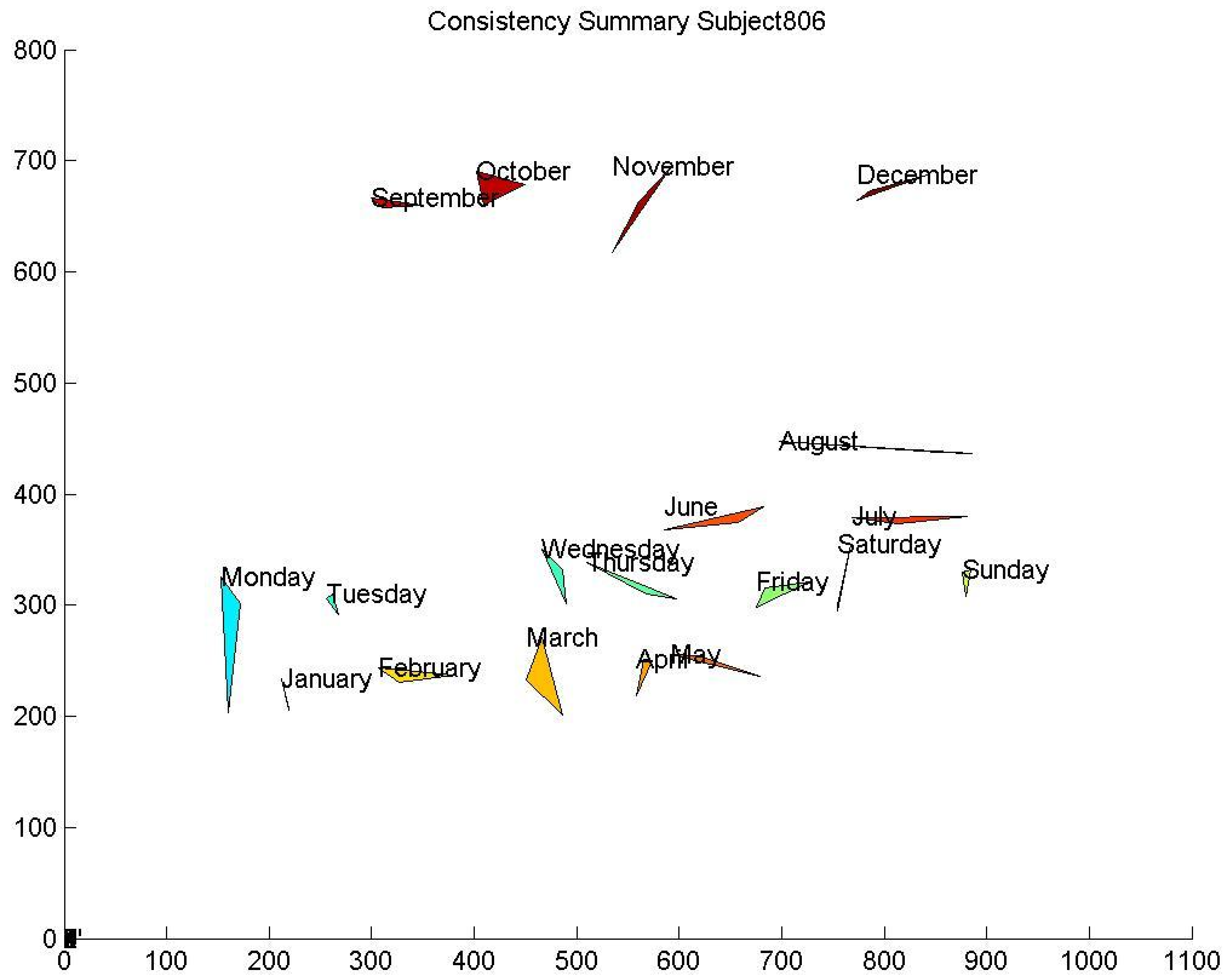

Consistency Summary Subject806
800
700
600
500
400
300
200
100
0
0
100
200
300
400
500
600
700
800
900
1000
1100
September
October
November
December
August
June
July
Saturday
Wednesday
Thursday
Friday
Sunday
Monday
Tuesday
March
April
May
January
February

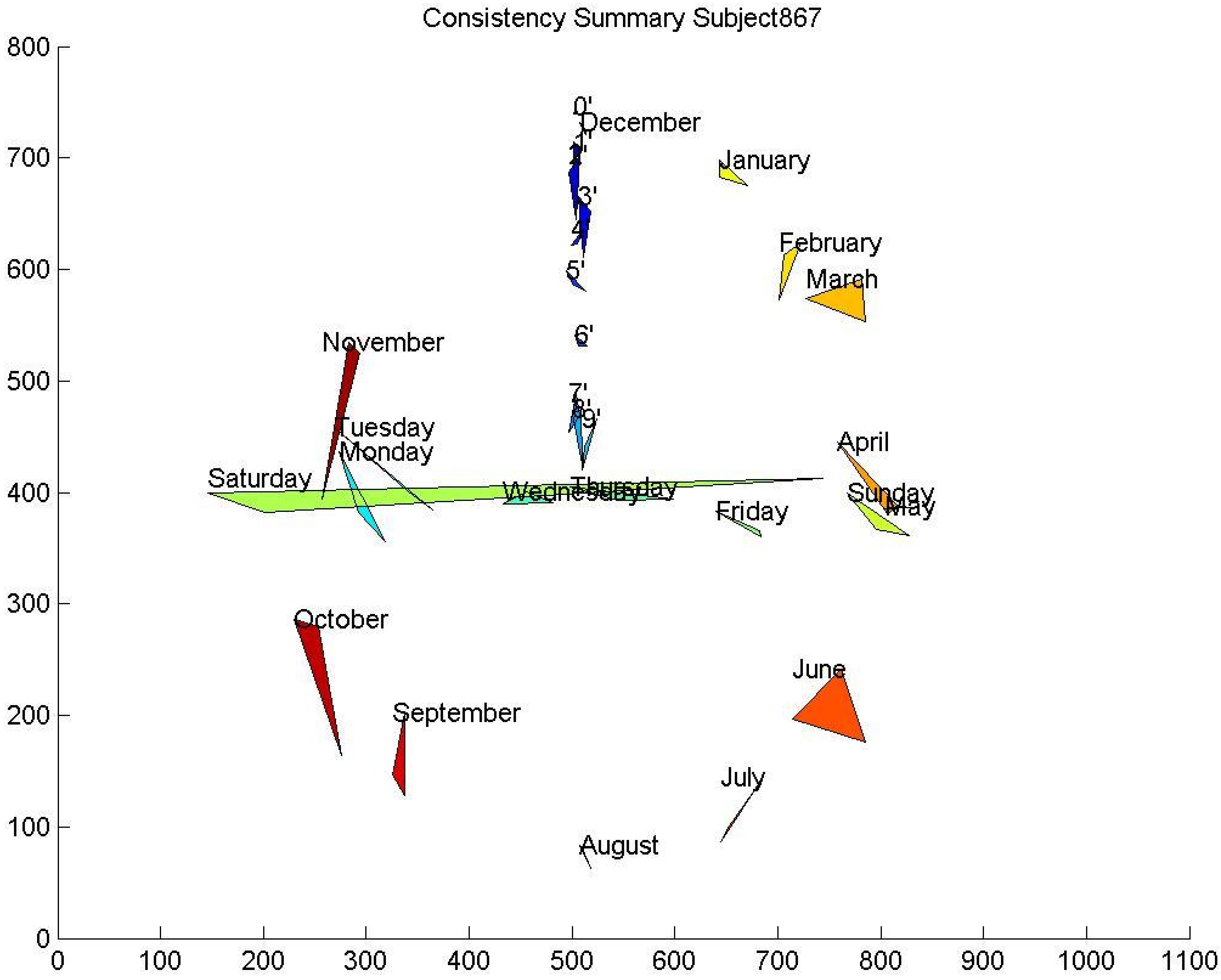

Consistency Summary Subject867
0'
December
1'
2'
January
3'
4'
February
5'
March
6'
November
7'
8'
9'
Tuesday
Monday
April
Saturday
Thursday
Wednesday
Sunday
May
Friday
October
June
September
July
August
800
700
600
500
400
300
200
100
0
0
100
200
300
400
500
600
700
800
900
1000
1100

Consistency Summary Subject887

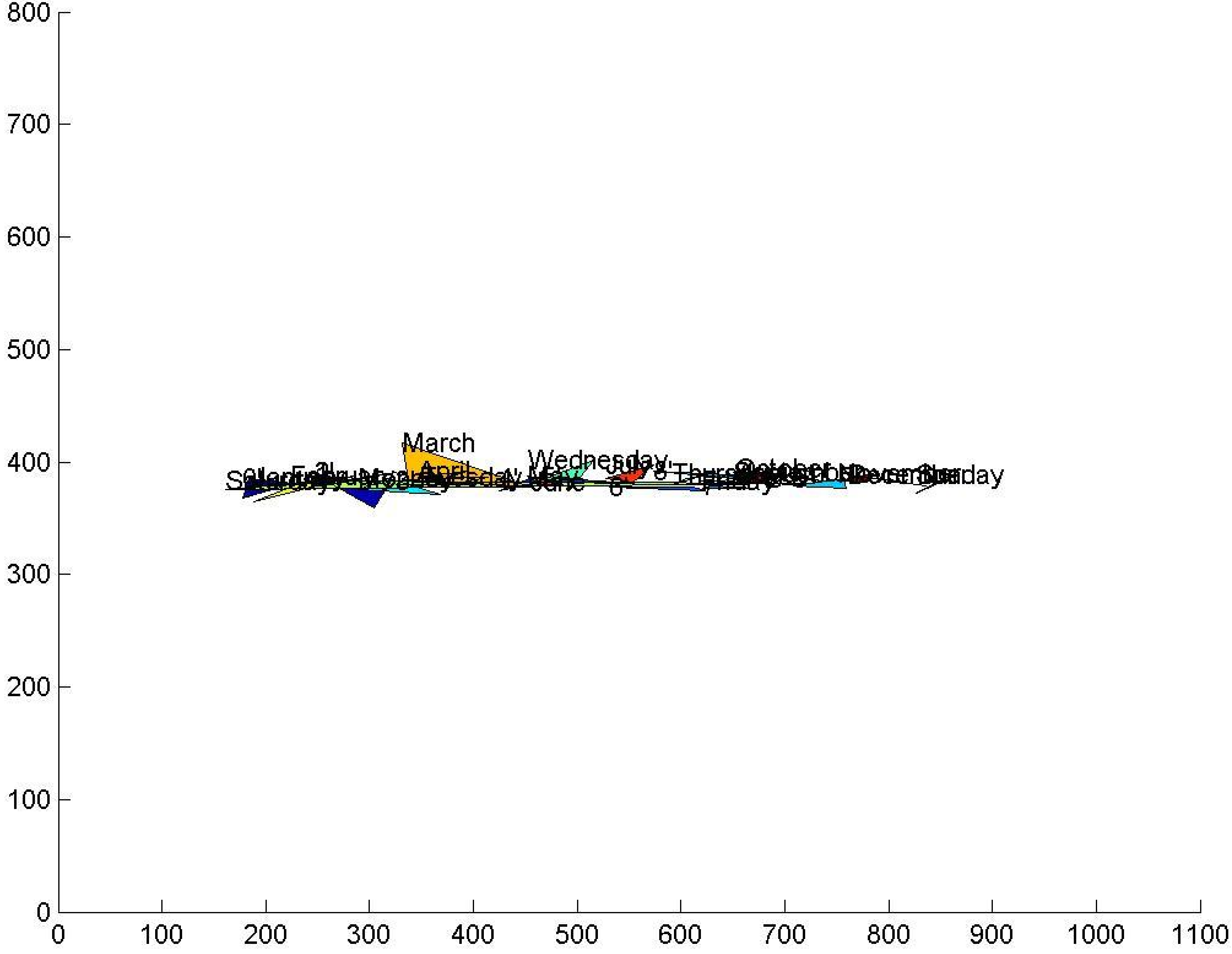

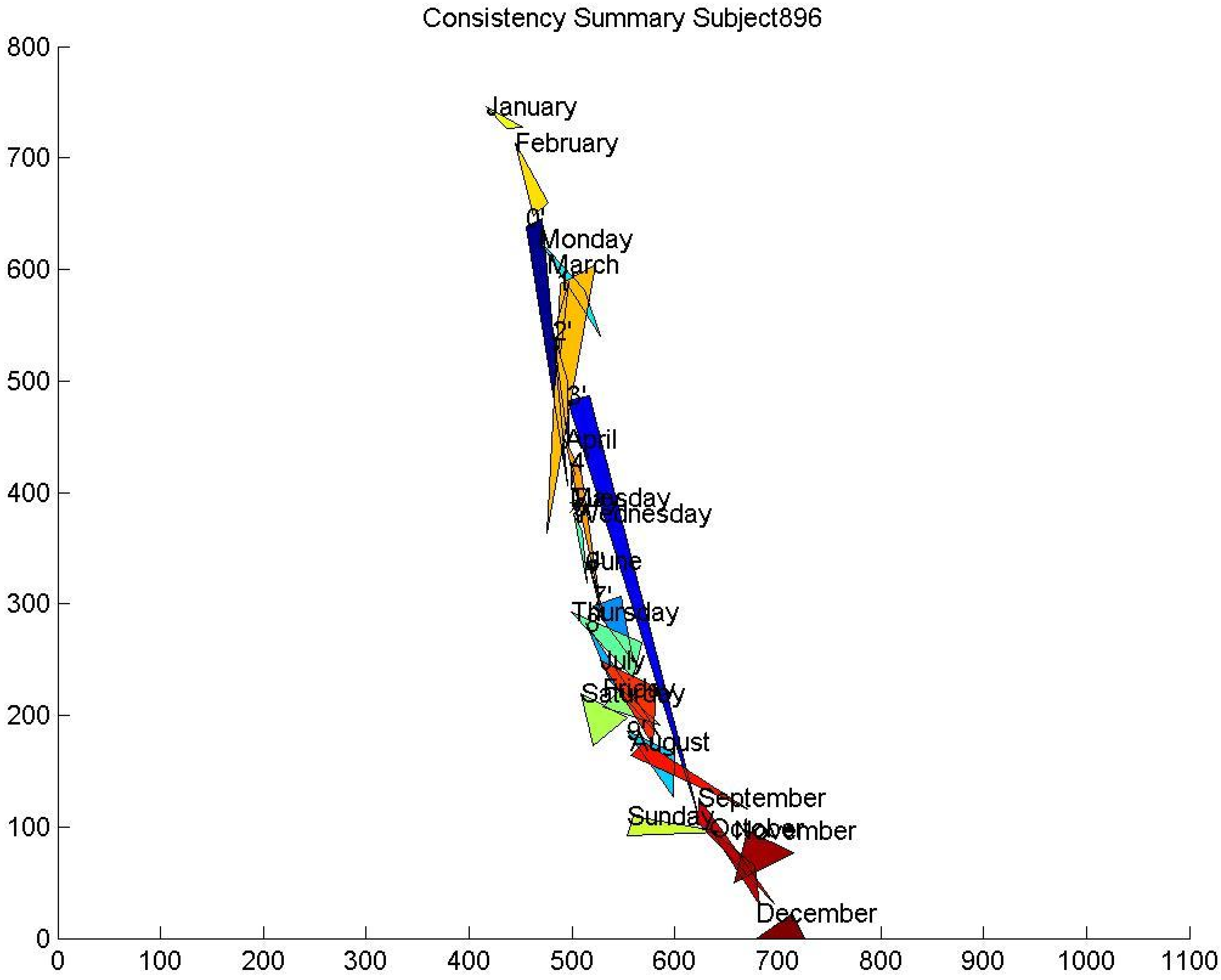

Consistency Summary Subject896
January
February
Monday
March
April
Wednesday
June
Thursday
July
August
September
Sunday
November
December
0
100
200
300
400
500
600
700
800
900
1000
1100

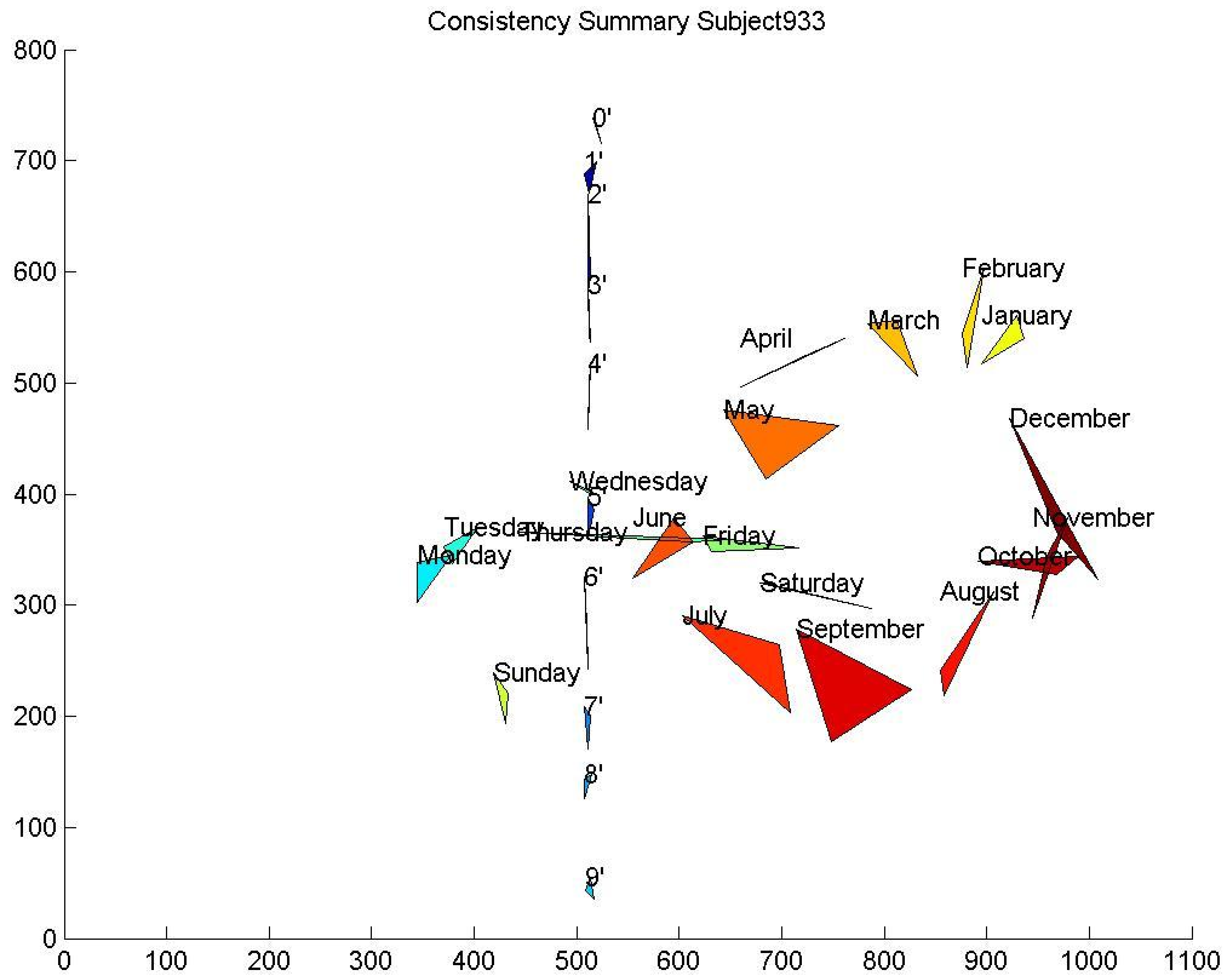

Consistency Summary Subject933
0'
1'
2'
3'
4'
5'
6'
7'
8'
9'
February
January
March
April
May
December
Wednesday
November
June
Tuesday
Thursday
Friday
Monday
October
Saturday
August
July
September
Sunday
800
700
600
500
400
300
200
100
0
0
100
200
300
400
500
600
700
800
900
1000
1100

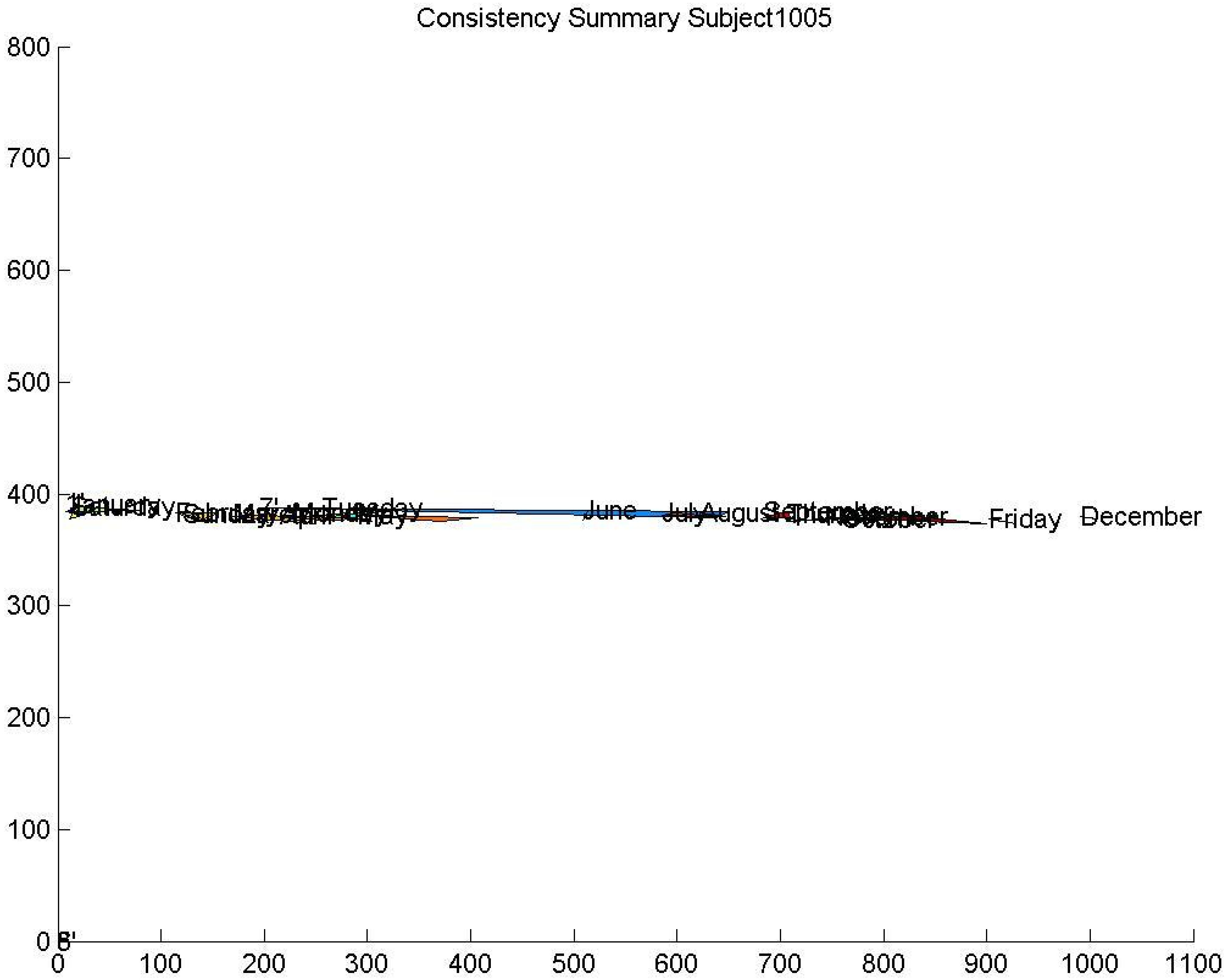

Consistency Summary Subject1005
800
700
600
500
400
300
200
100
0
0
100
200
300
400
500
600
700
800
900
1000
1100
June
Friday
December

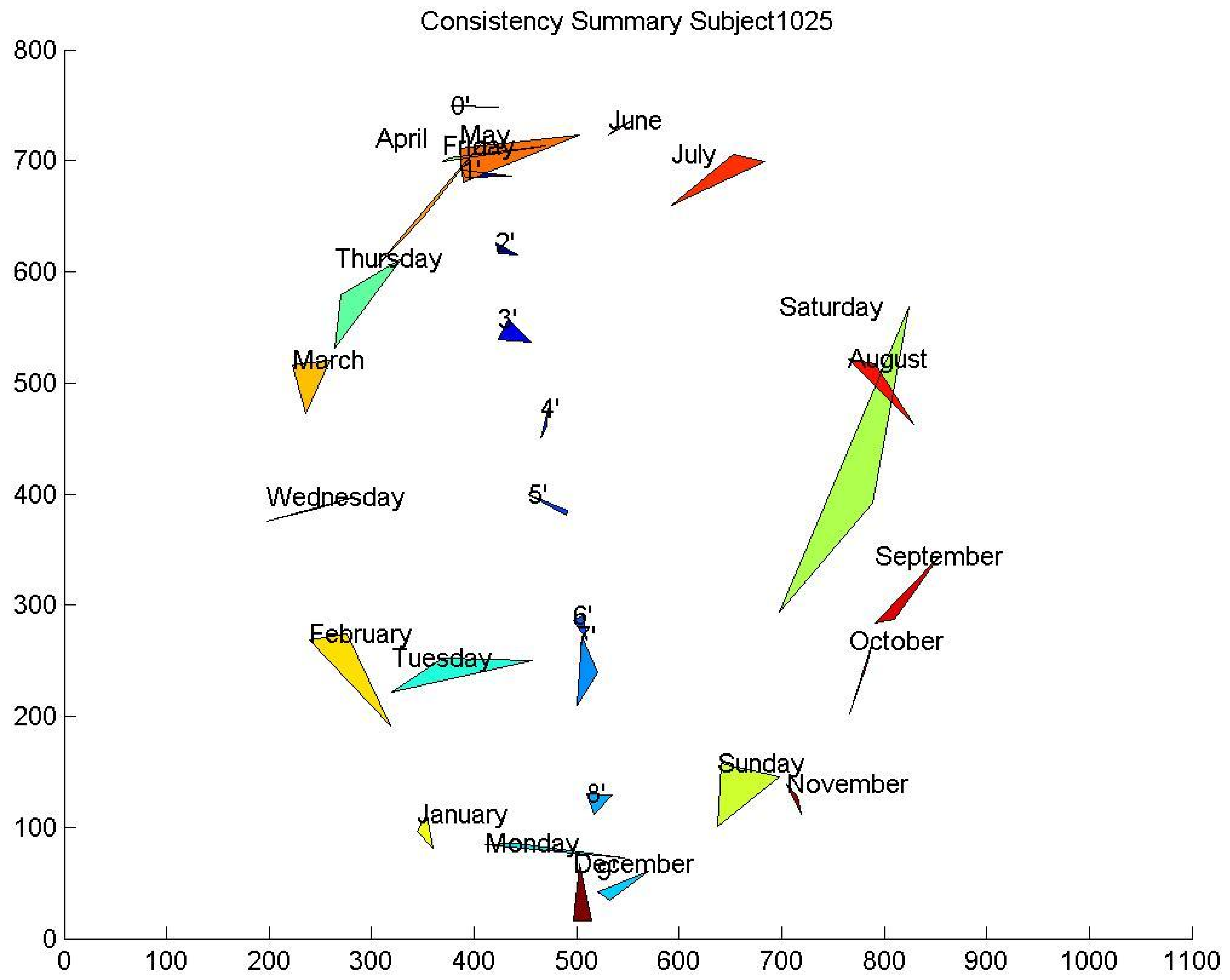
Consistency Summary Subject1025
0'
June
April
May
Friday
July
1'
2'
Thursday
Saturday
3'
March
August
4'
Wednesday
5'
September
6'
7'
February
October
Tuesday
Sunday
November
8'
January
Monday
December
9'
0
100
200
300
400
500
600
700
800
900
1000
1100

Consistency Summary Subject1027

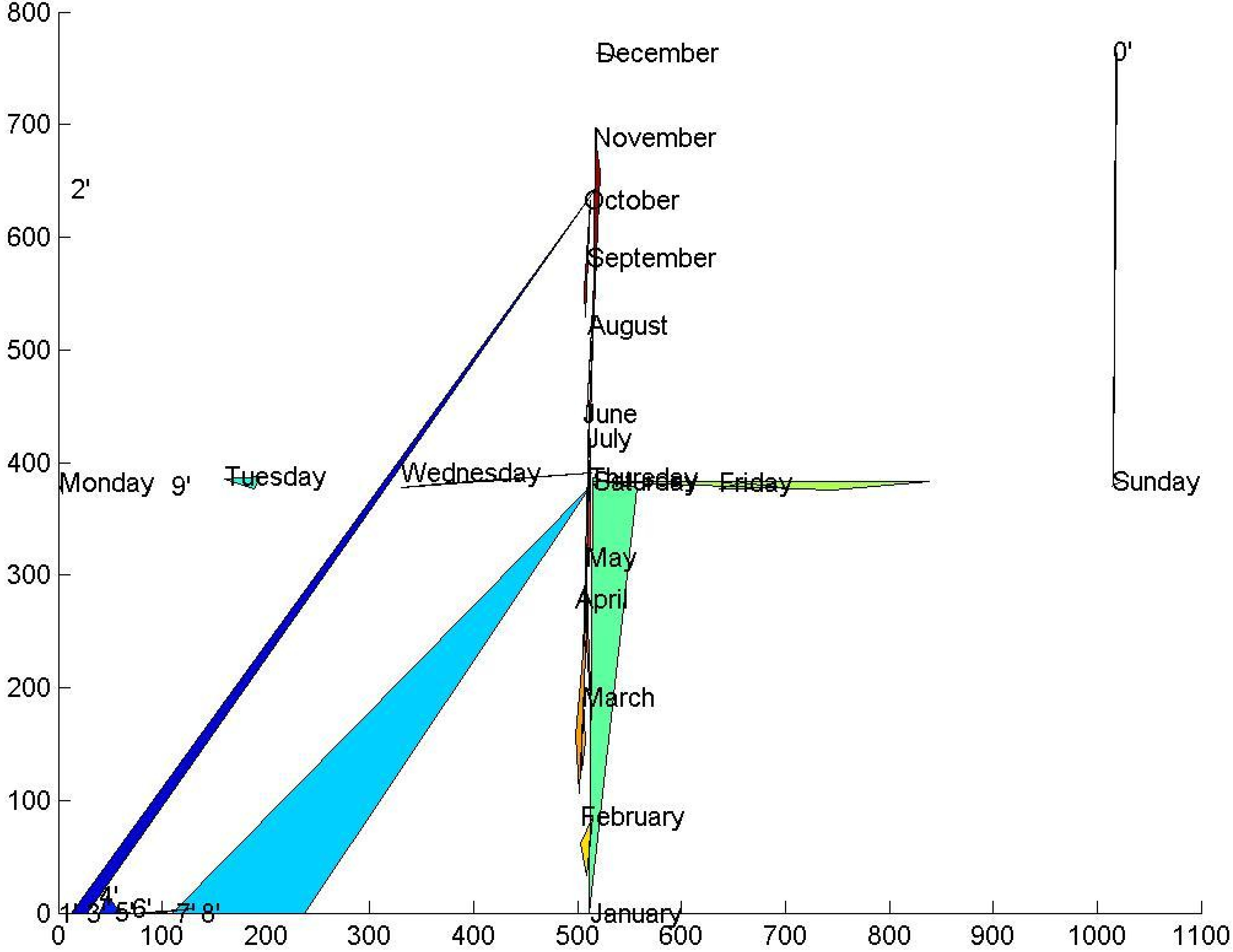

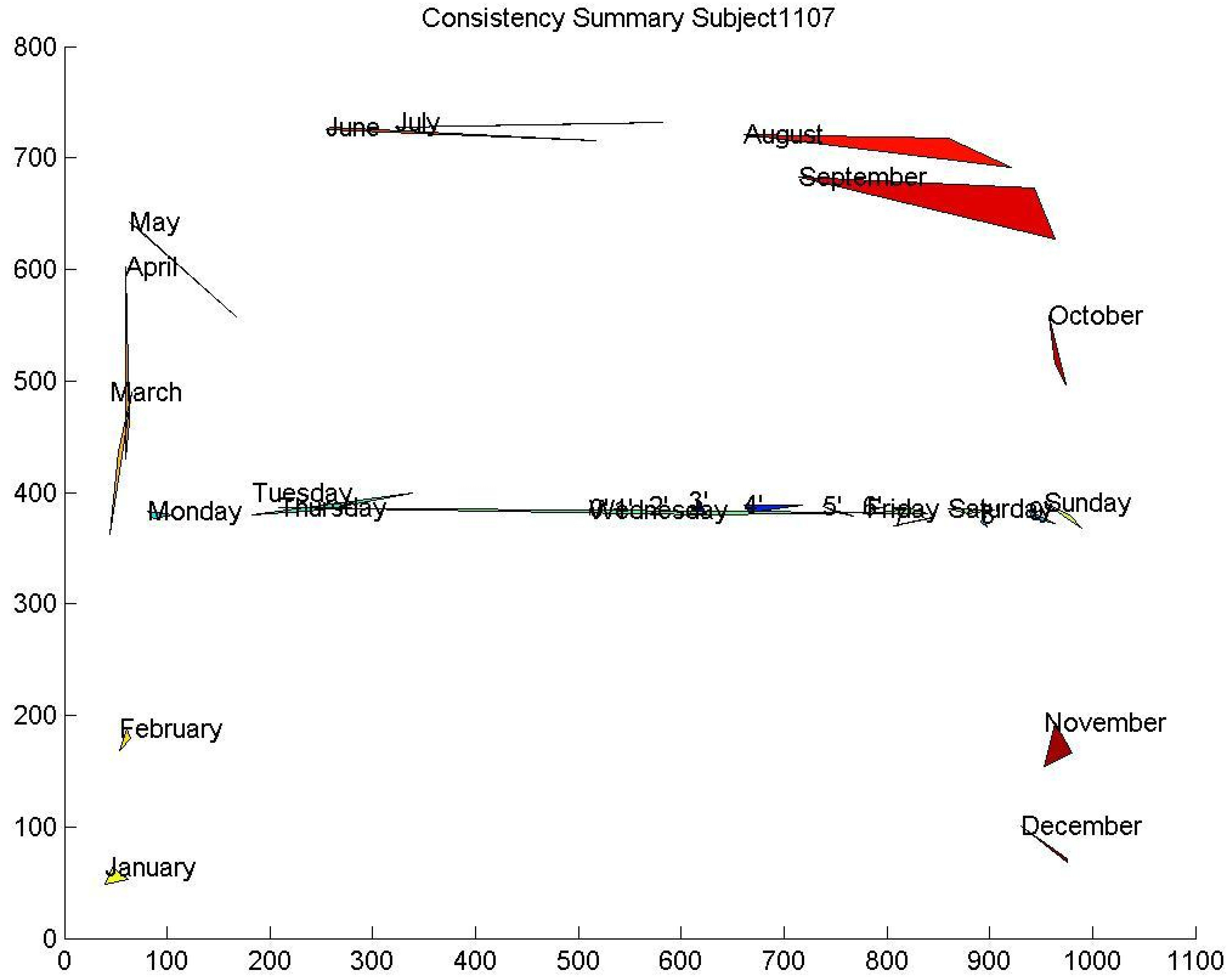
Consistency Summary Subject1107
January
February
March
April
May
June
July
August
September
October
November
December
Monday
Tuesday
Thursday
Wednesday
Friday
Saturday
Sunday
3'
4'
5'
0
100
200
300
400
500
600
700
800
900
1000
1100

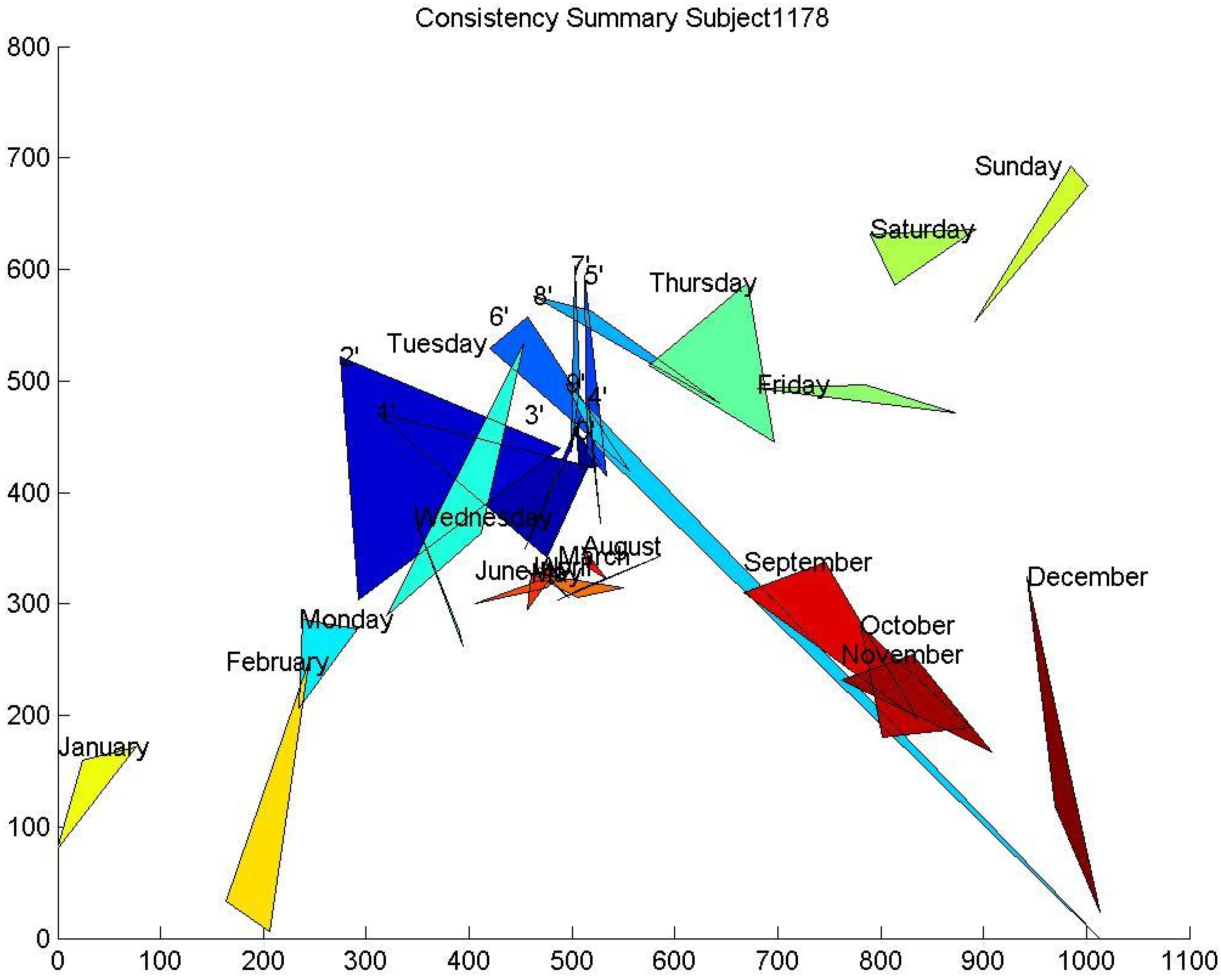
Consistency Summary Subject1178
800
700
600
500
400
300
200
100
0
0
100
200
300
400
500
600
700
800
900
1000
1100
Sunday
Saturday
Thursday
Friday
Tuesday
Wednesday
Monday
February
January
June
March
August
September
October
November
December
2'
3'
4'
5'
6'
7'
8'
9'

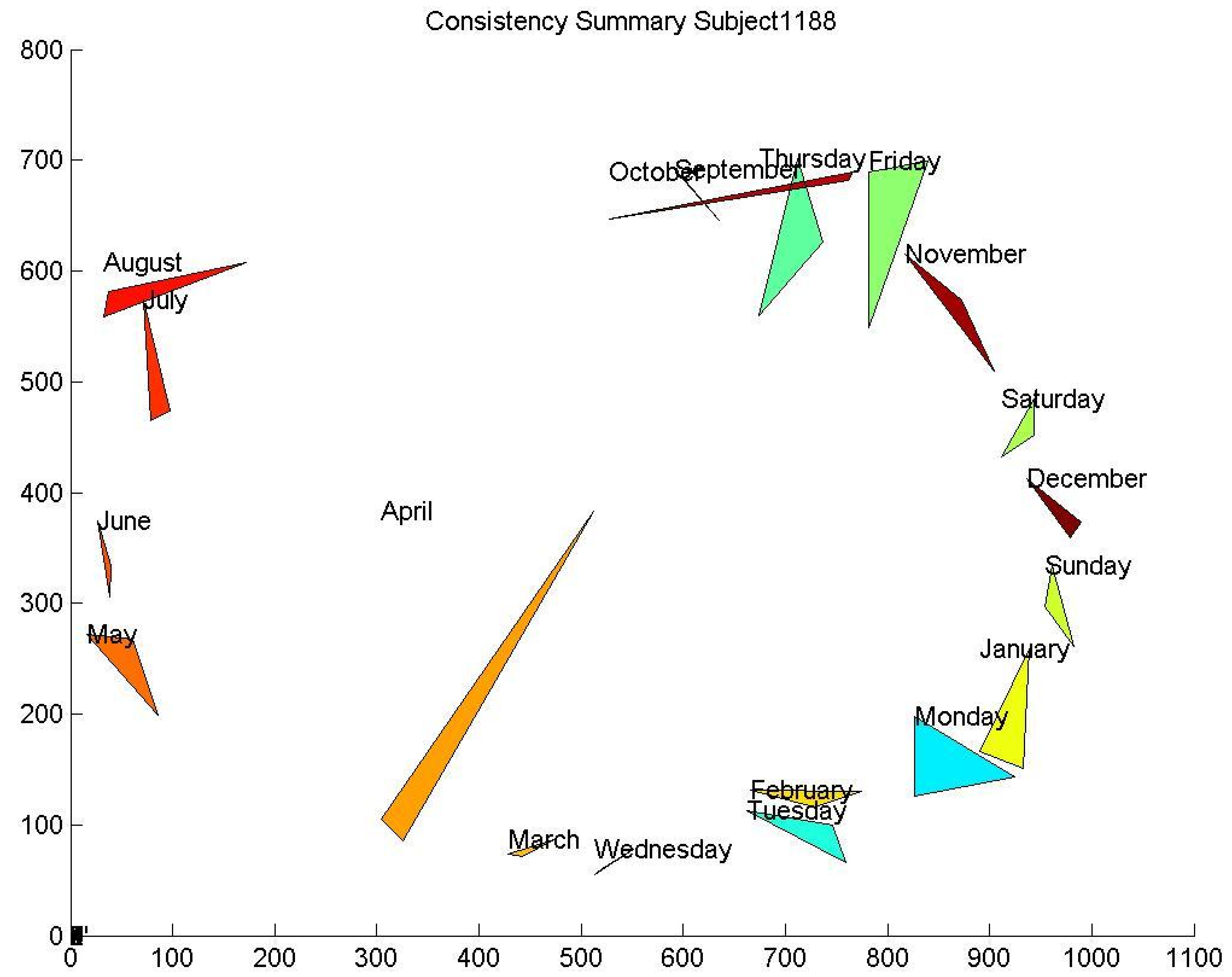
Consistency Summary Subject1188
800
700
600
500
400
300
200
100
0
0
100
200
300
400
500
600
700
800
900
1000
1100
October
September
Thursday
Friday
August
July
November
Saturday
December
June
April
Sunday
May
January
Monday
February
Tuesday
March
Wednesday

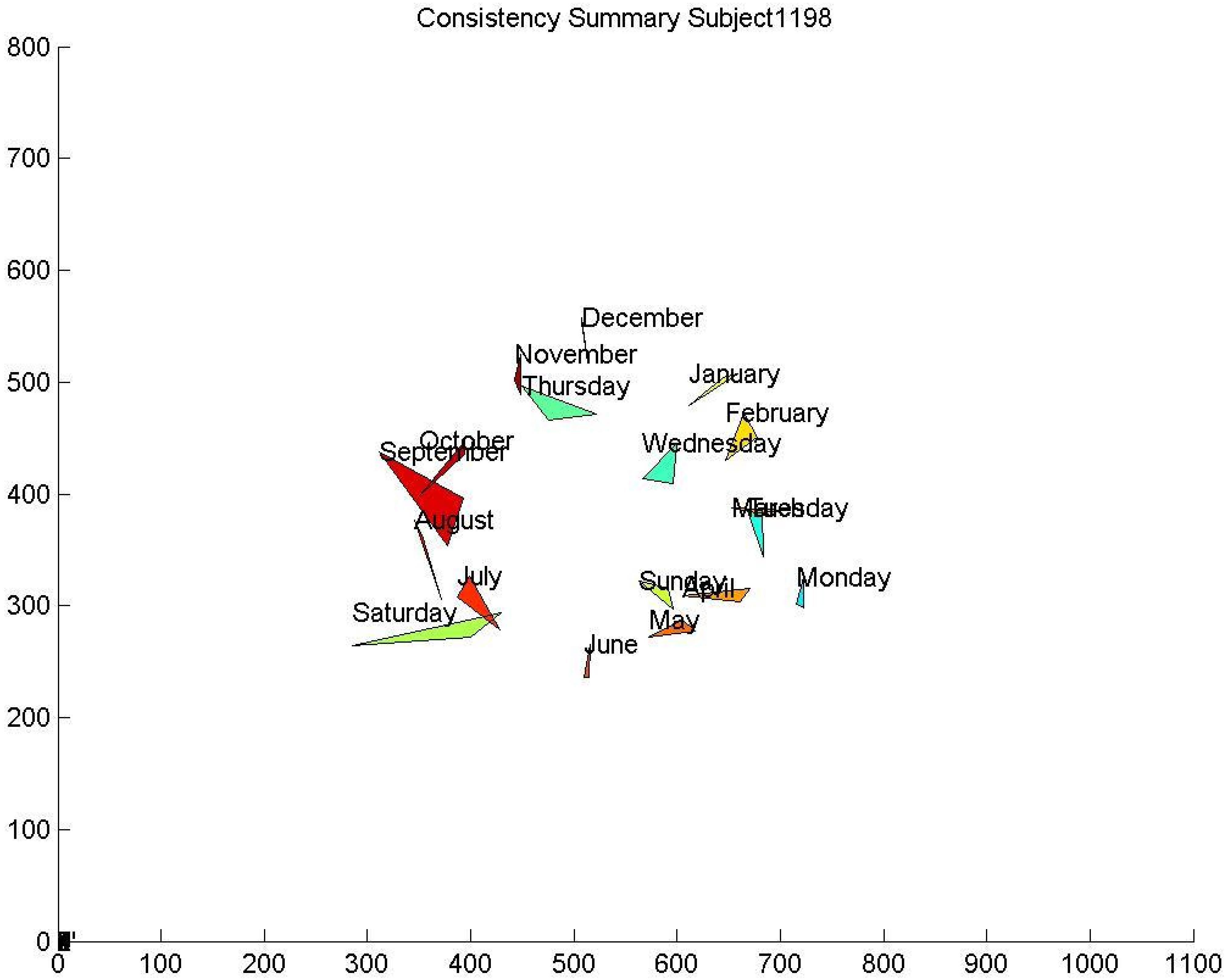

Consistency Summary Subject1198
800
700
600
500
400
300
200
100
0
0
100
200
300
400
500
600
700
800
900
1000
1100
December
November
Thursday
January
February
October
September
Wednesday
March
Tuesday
August
July
Monday
Sunday
April
Saturday
May
June

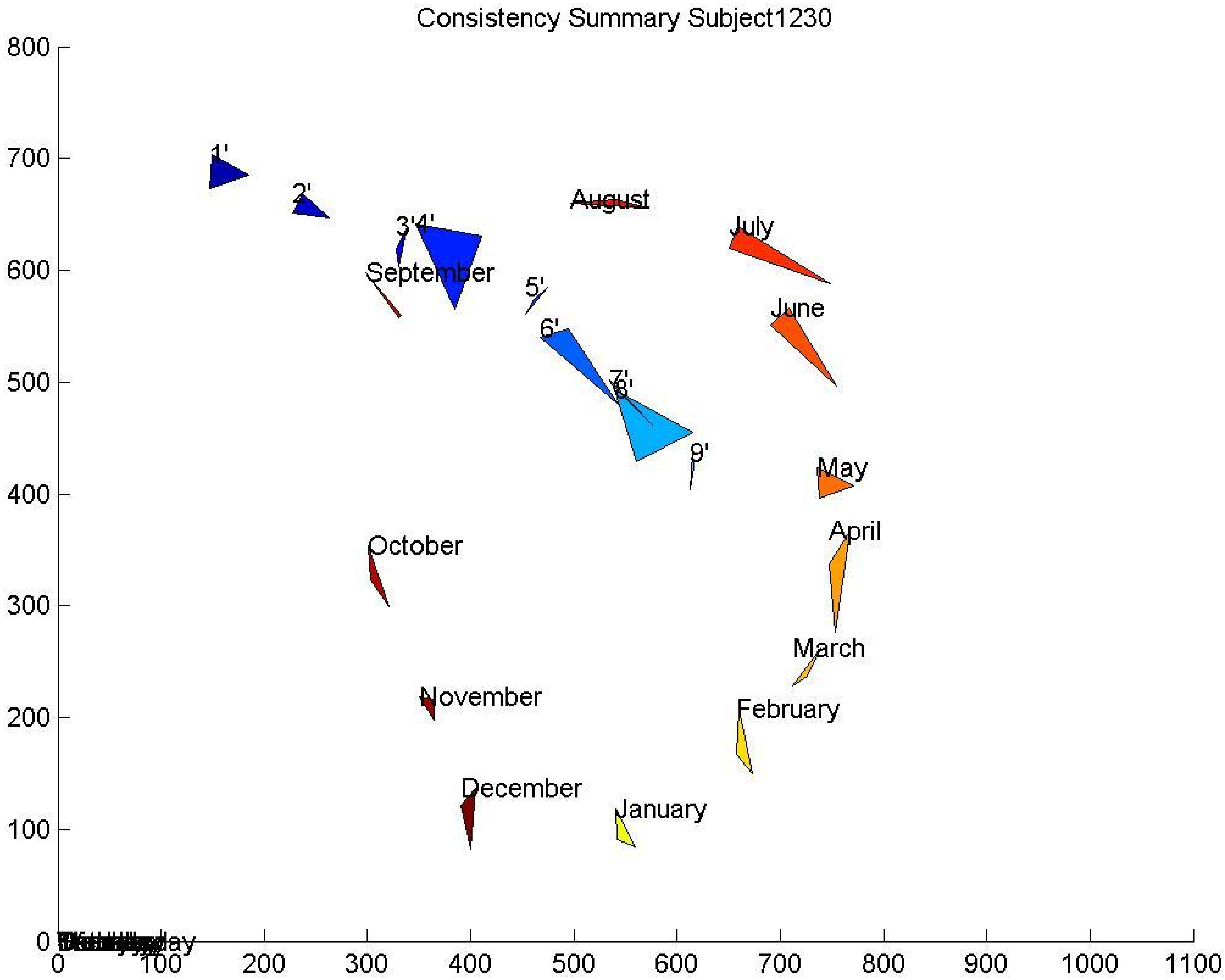

Consistency Summary Subject1230
800
700
600
500
400
300
200
100
0
0
100
200
300
400
500
600
700
800
900
1000
1100
1'
2'
3'4'
5'
6'
7'
8'
9'
August
July
June
September
May
April
March
February
January
December
November
October

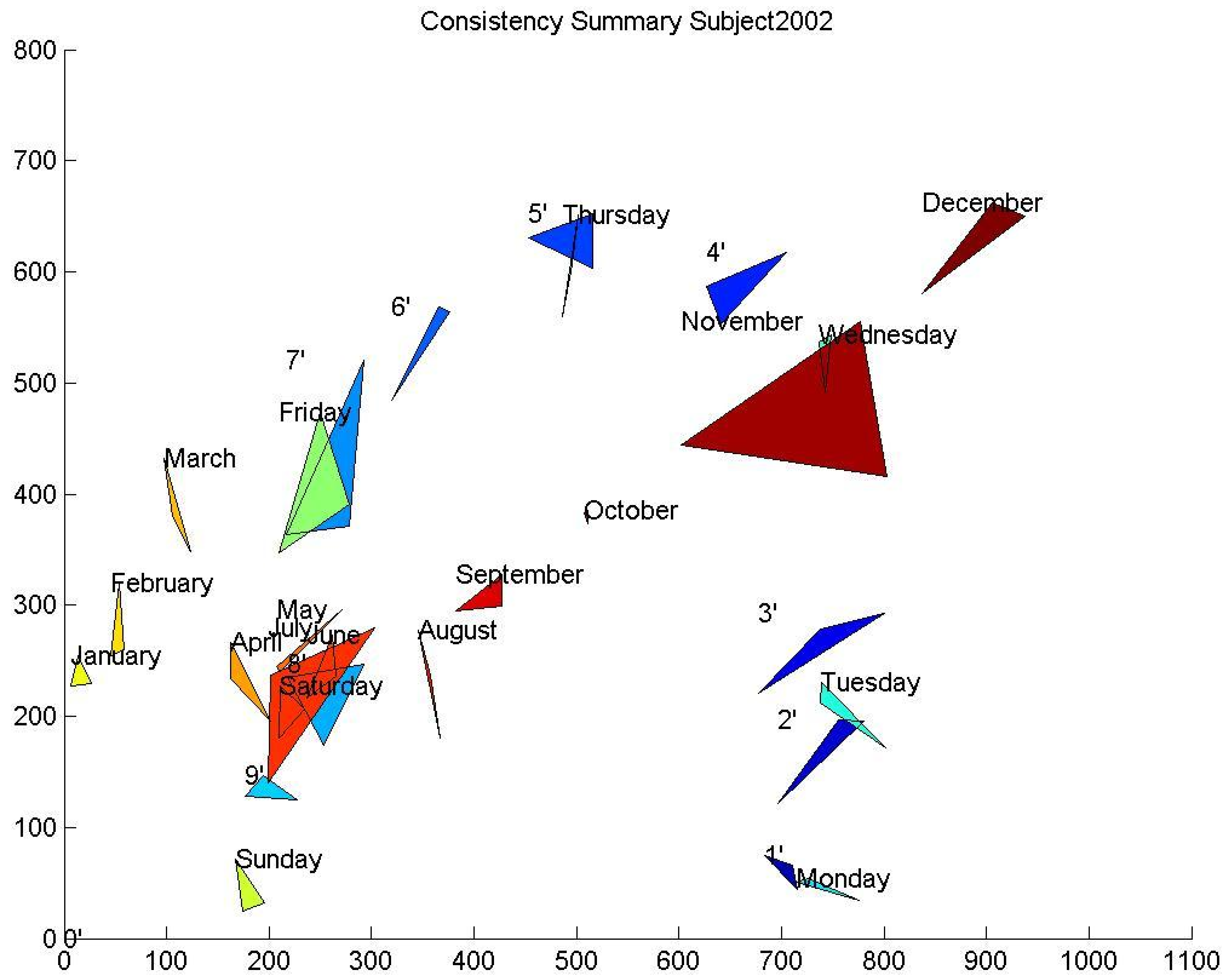

Consistency Summary Subject2002
800
700
600
500
400
300
200
100
0
0
100
200
300
400
500
600
700
800
900
1000
1100
5'
Thursday
December
4'
6'
November
Wednesday
7'
Friday
March
October
September
February
May
July
June
April
August
January
8'
Saturday
3'
Tuesday
2'
9'
Sunday
1'
Monday
0'

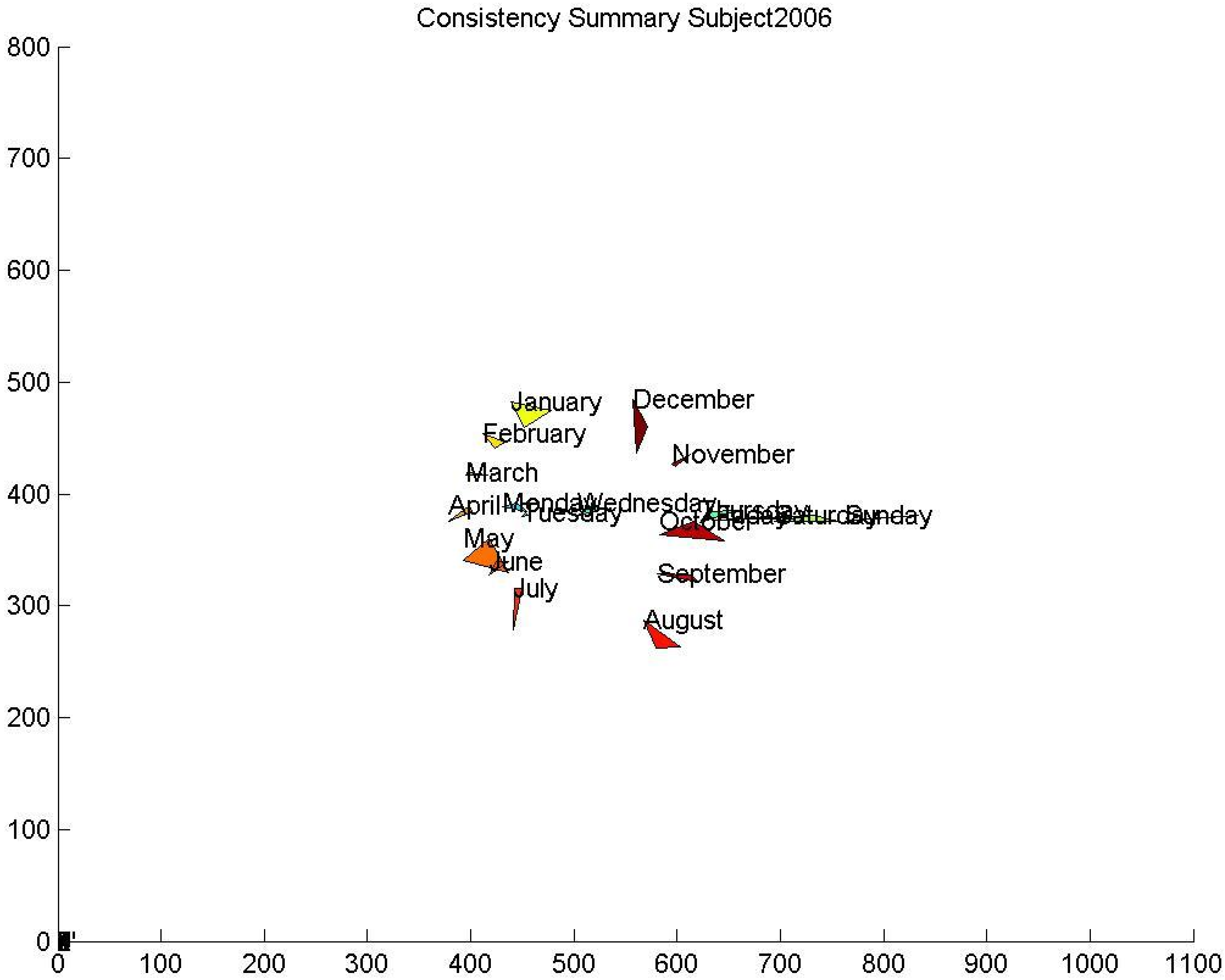
Consistency Summary Subject2006
800
700
600
500
400
300
200
100
0
100
200
300
400
500
600
700
800
900
1000
1100
January
December
February
November
March
April
Wednesday
Tuesday
October
May
June
July
September
August
Sunday

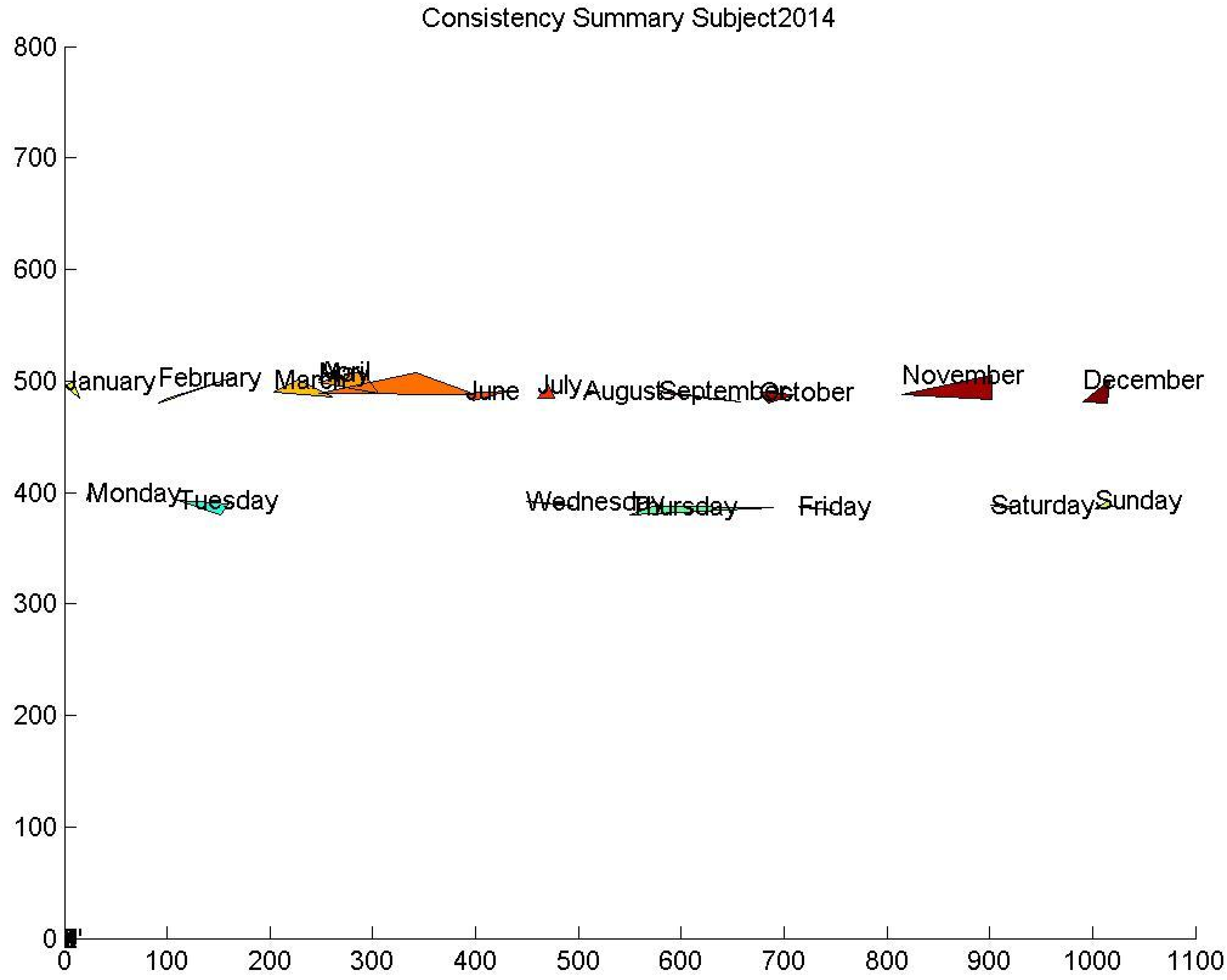
Consistency Summary Subject2014
800
700
600
500
400
300
200
100
0
0
100
200
300
400
500
600
700
800
900
1000
1100
January
February
March
June
July
August
September
October
November
December
Monday
Tuesday
Wednesday
Thursday
Friday
Saturday
Sunday
