# Supplementary material for: The sensitivity and specificity of a diagnostic test of sequence-space synesthesia
Source: Behav Res Methods. 2015 Nov 11;48(4):1476–81. doi: 10.3758/s13428-015-0656-2 (PMC5101286; doi:10.3758/s13428-015-0656-2)
Supplement: Supplementary file 2 — (PDF 2641 kb) [file 13428_2015_656_MOESM2_ESM.pdf]

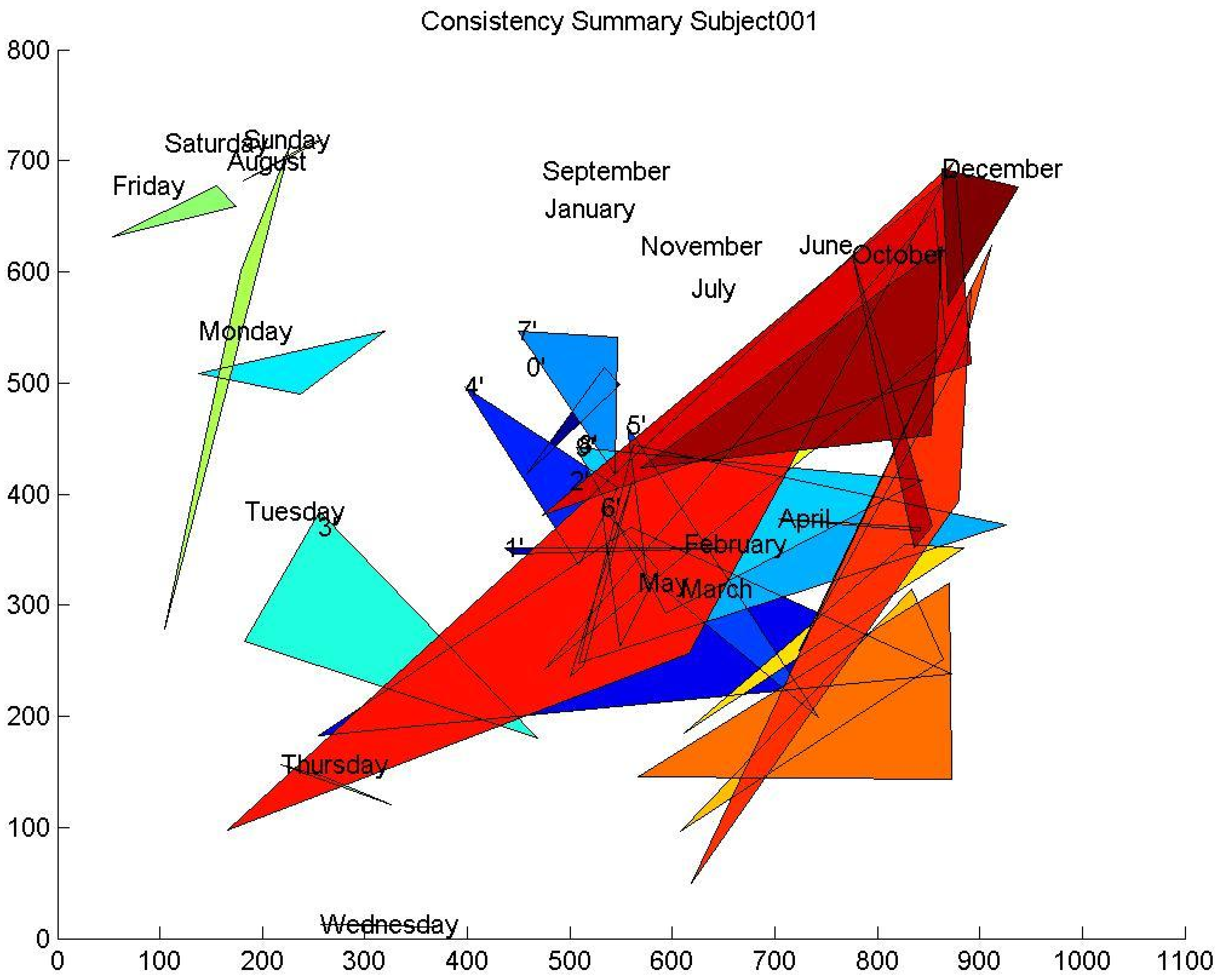

Consistency Summary Subject001
Saturday
Sunday
August
Friday
September
January
November
June
October
December
July
Monday
7'
0'
4'
5'
8'
2'
6'
Tuesday
3'
1'
April
February
May
March
Thursday
Wednesday
0
100
200
300
400
500
600
700
800
900
1000
1100

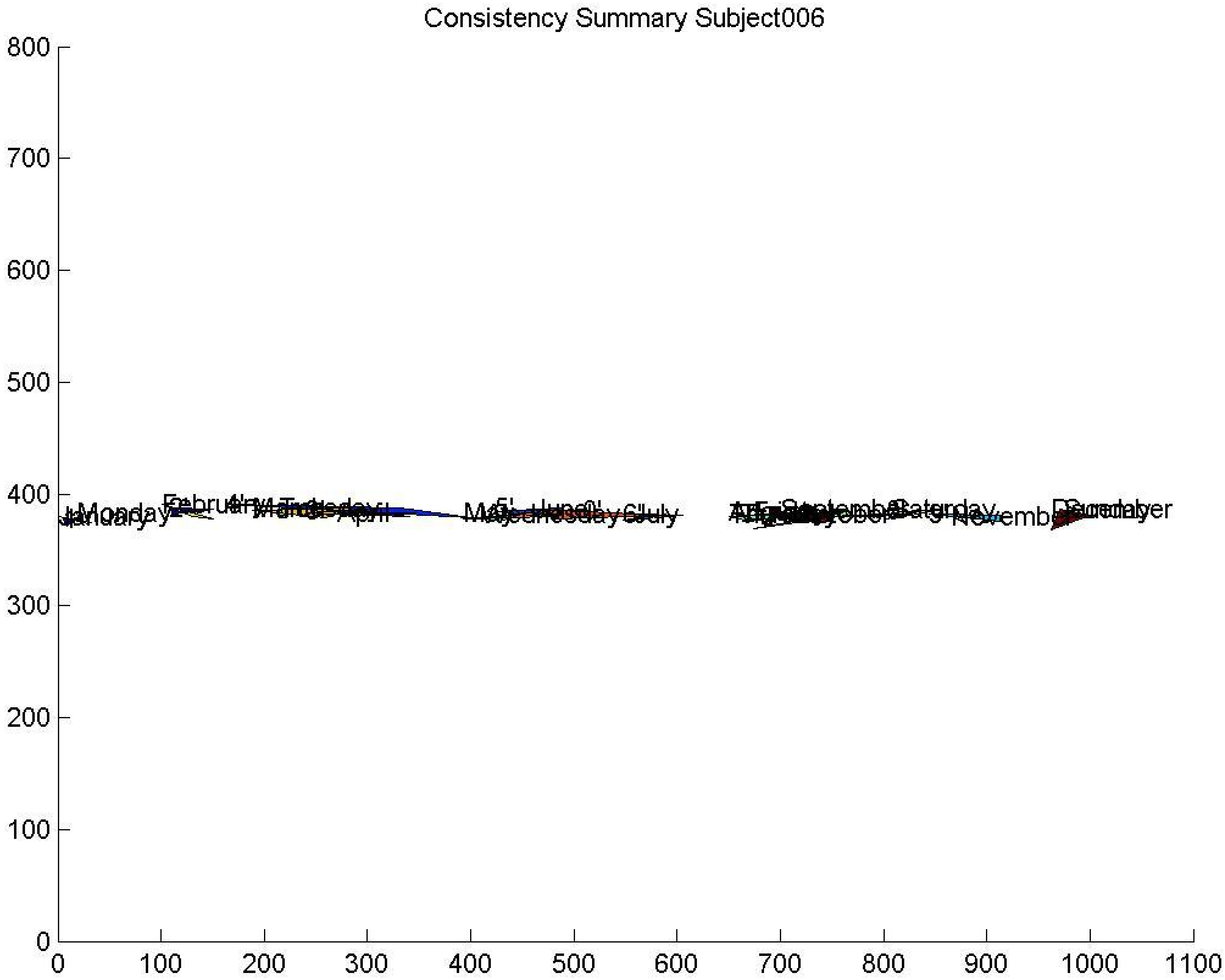
Consistency Summary Subject006
800
700
600
500
400
300
200
100
0
0
100
200
300
400
500
600
700
800
900
1000
1100
Monday
January
February
April
July
November
Saturday

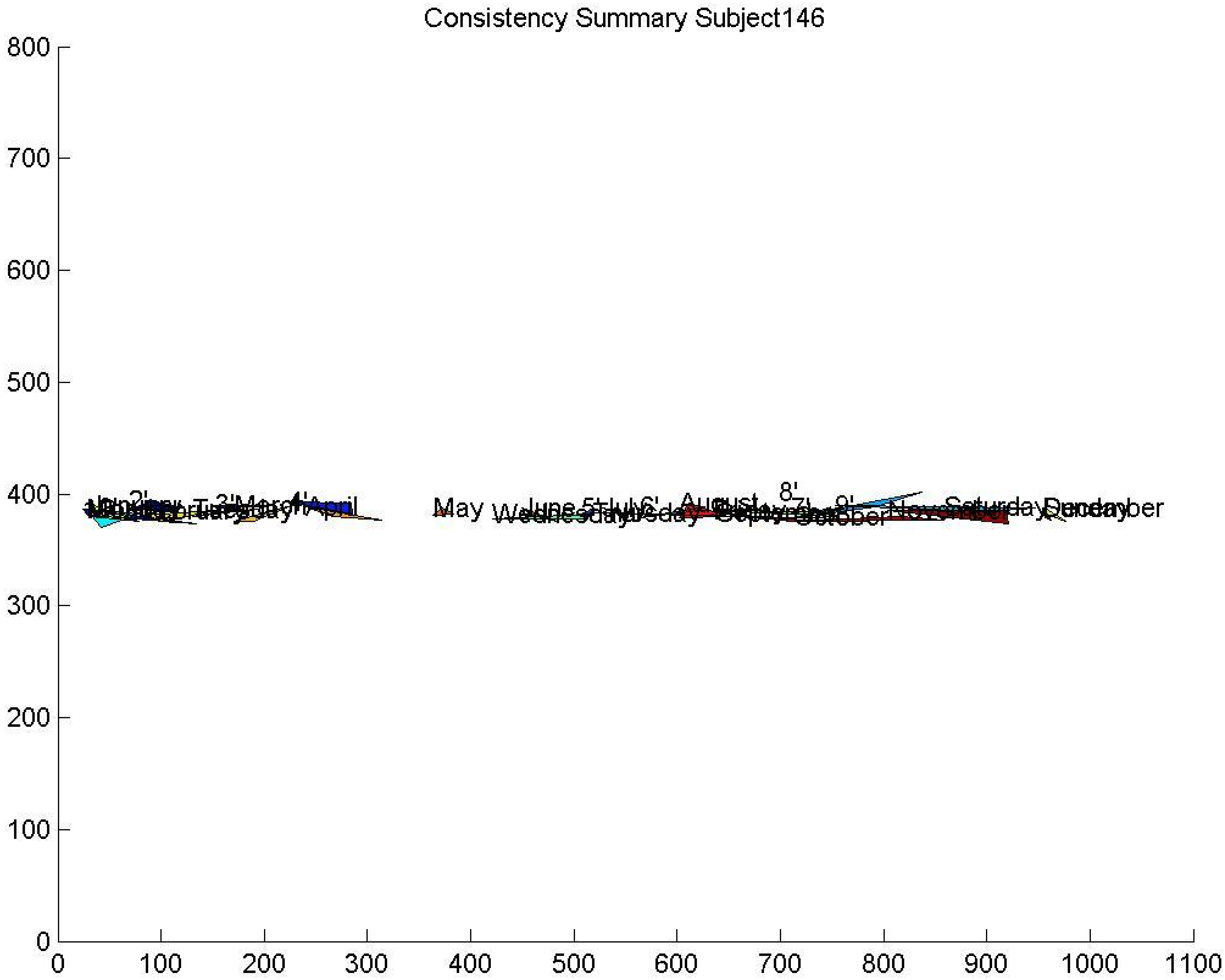

Consistency Summary Subject146
May
8'

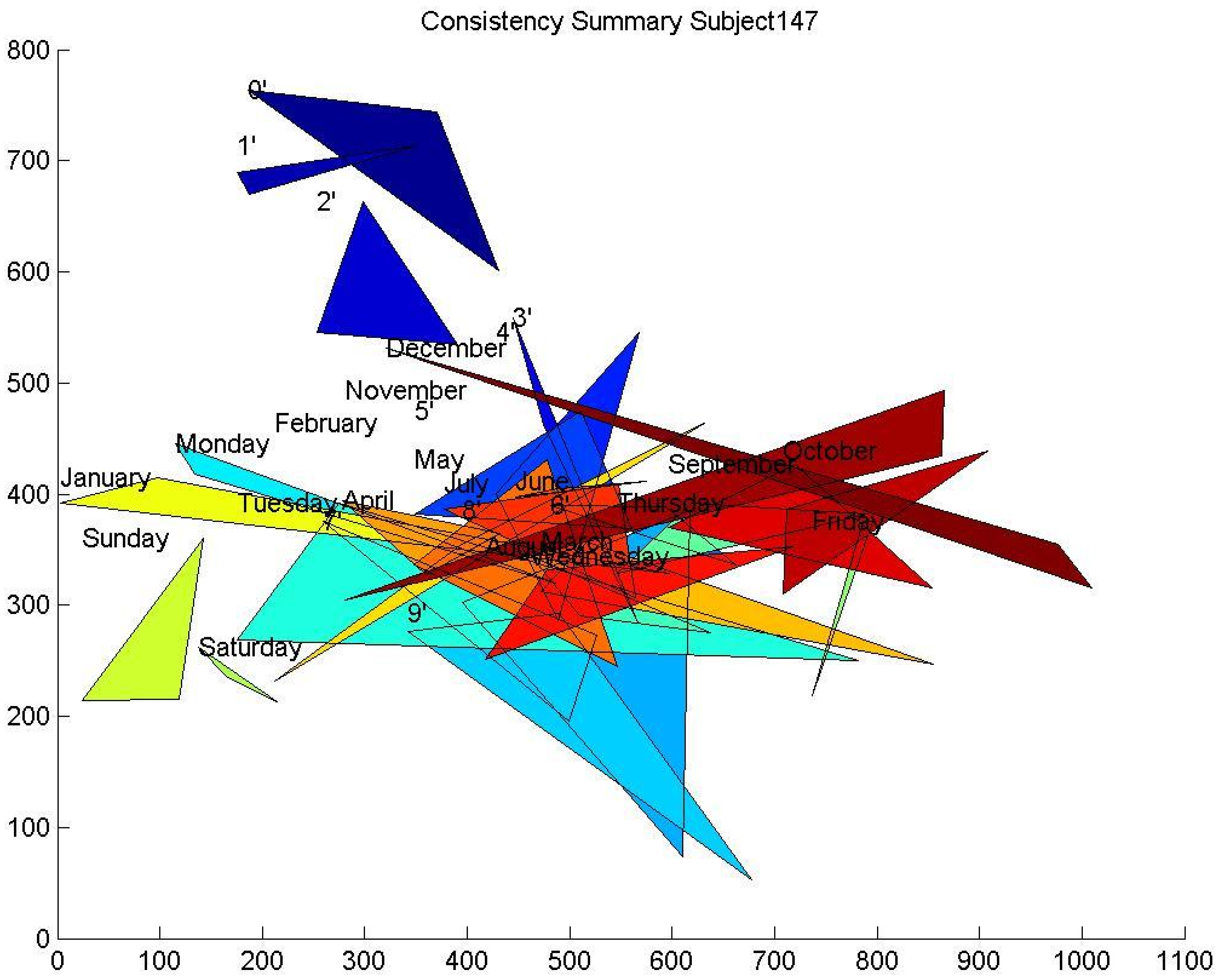
Consistency Summary Subject147
0'
1'
2'
3'
4'
5'
6'
7'
8'
9'
December
November
February
Monday
January
May
July
June
September
October
Tuesday
April
Thursday
Friday
Sunday
March
Wednesday
Saturday
0
100
200
300
400
500
600
700
800
0
100
200
300
400
500
600
700
800
900
1000
1100

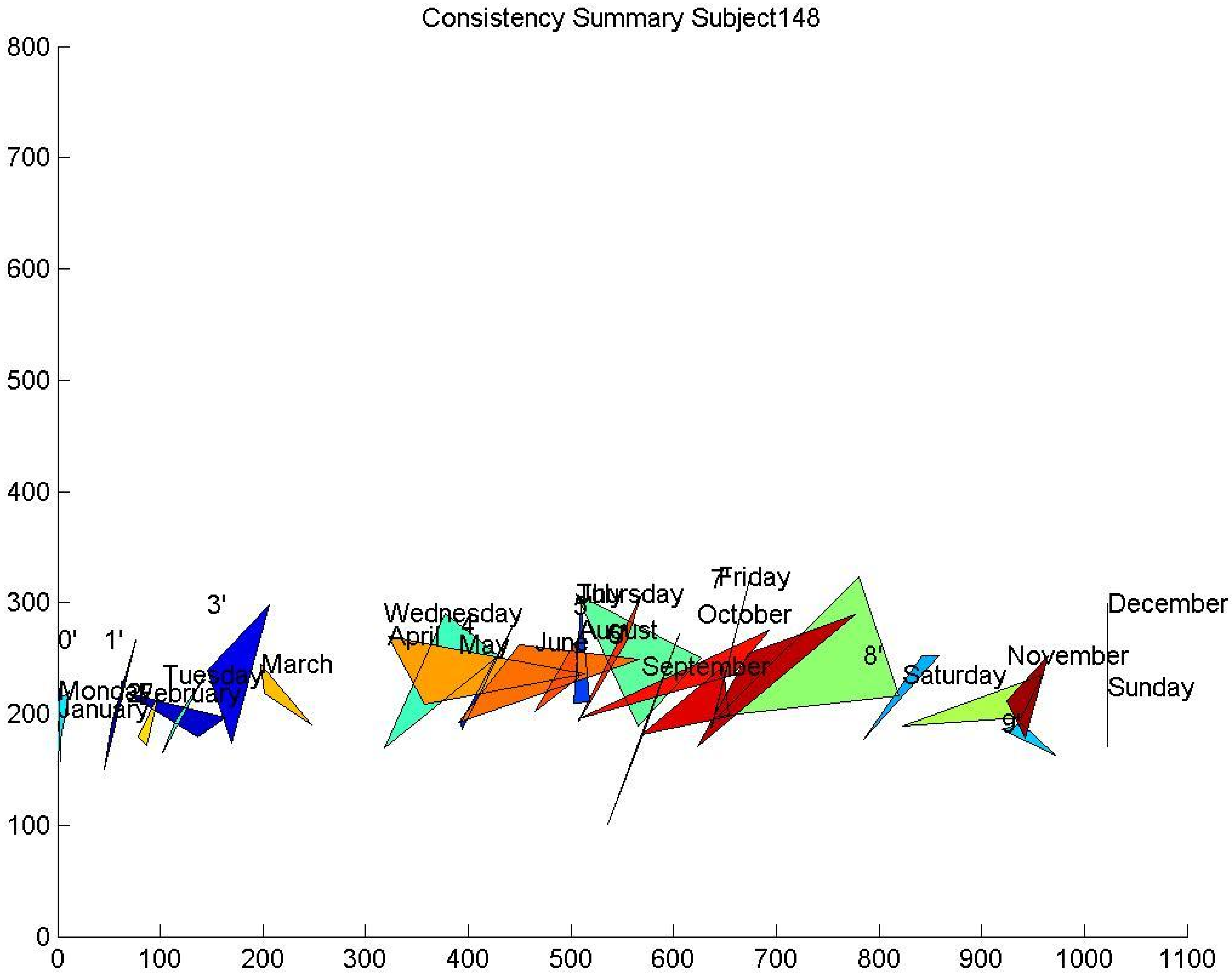

Consistency Summary Subject148
0'
1'
3'
Monday
January
February
Tuesday
March
Wednesday
April
4'
May
June
July
Thursday
August
September
Friday
October
8'
Saturday
November
9'
December
Sunday
0
100
200
300
400
500
600
700
800
900
1000
1100

Consistency Summary Subject149

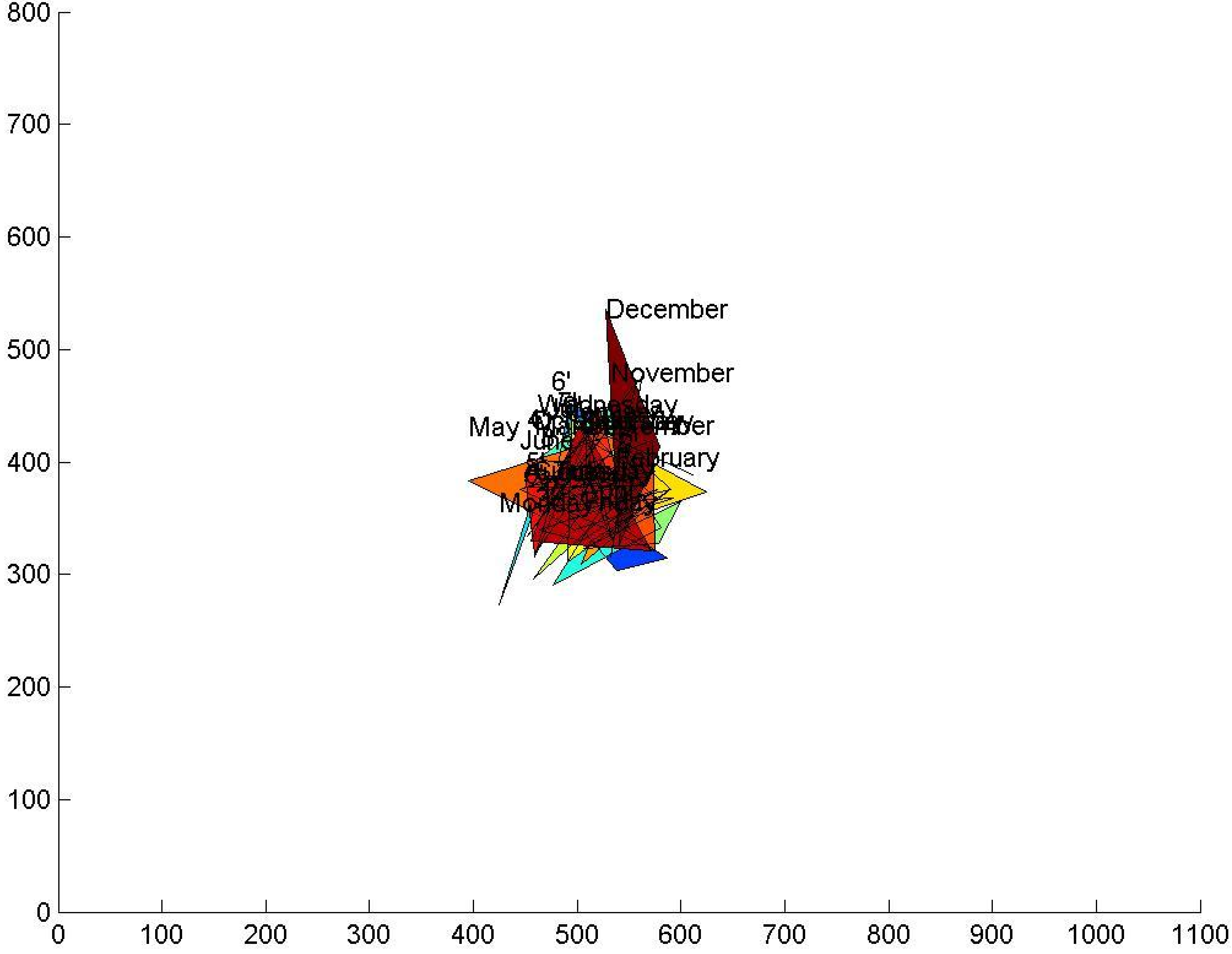

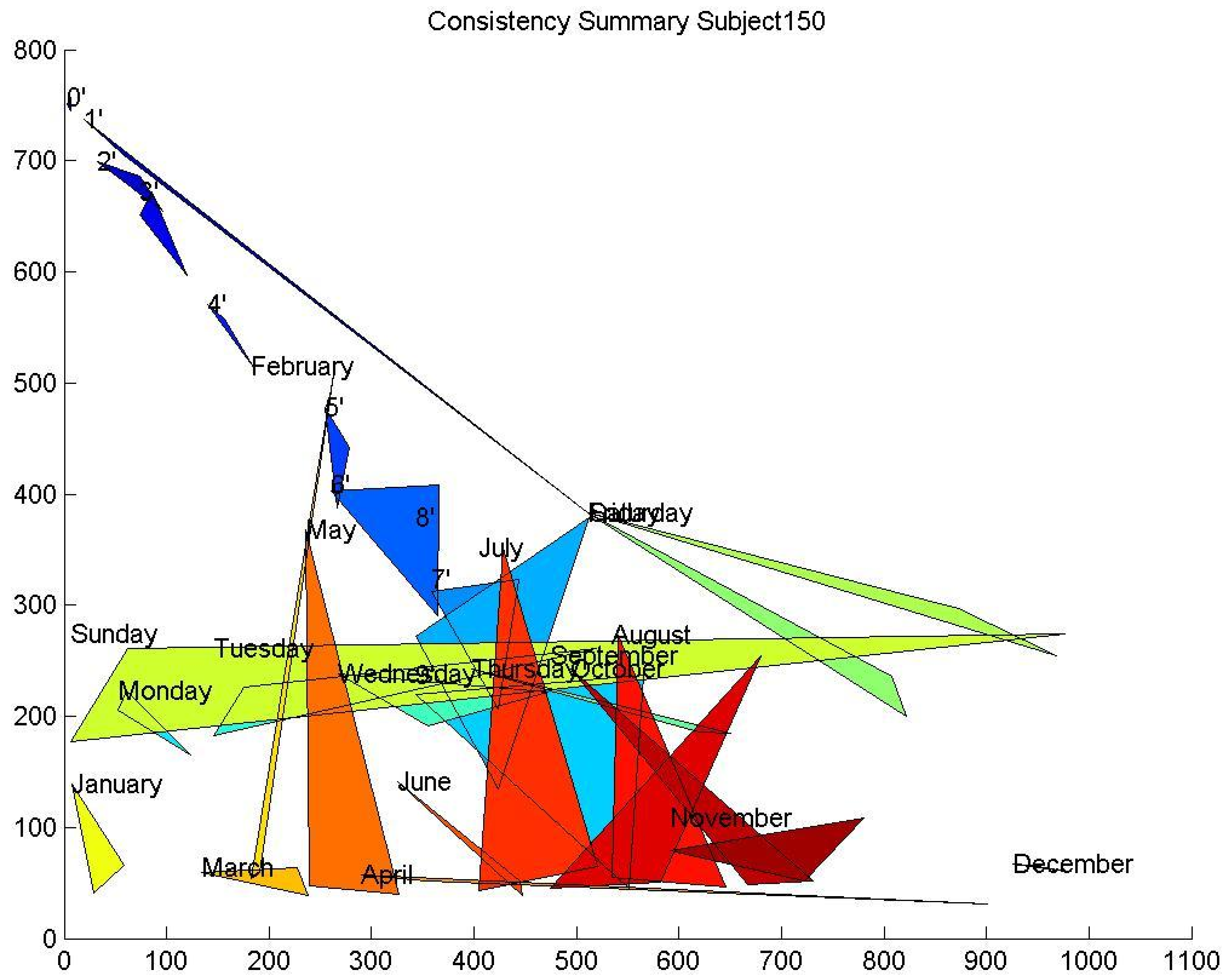
Consistency Summary Subject150
800
700
600
500
400
300
200
100
0
0
100
200
300
400
500
600
700
800
900
1000
1100
0'
1'
2'
3'
4'
5'
6'
7'
8'
February
May
July
Saturday
Sunday
Monday
Tuesday
Wednesday
Thursday
August
September
October
January
March
April
June
November
December

Consistency Summary Subject156

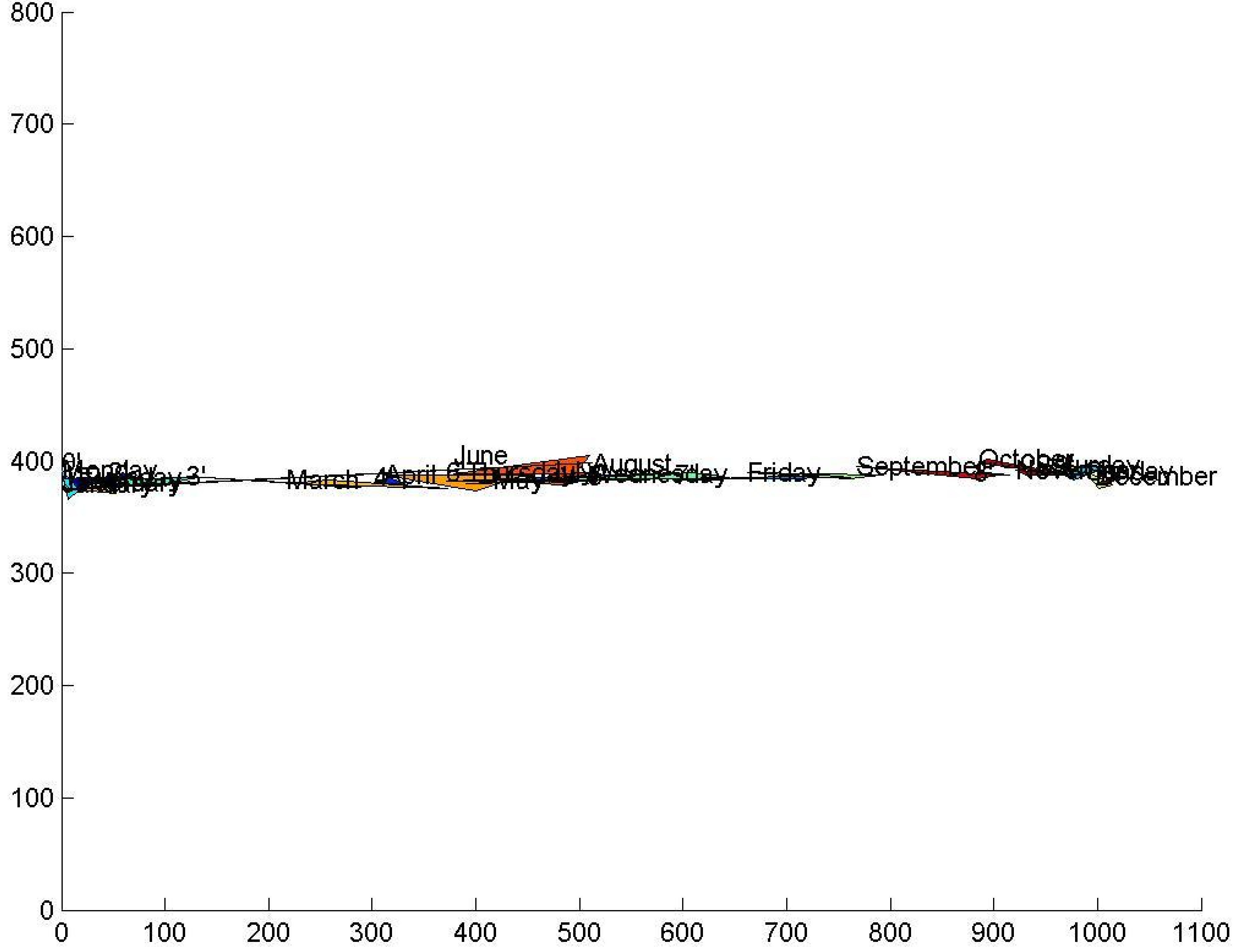

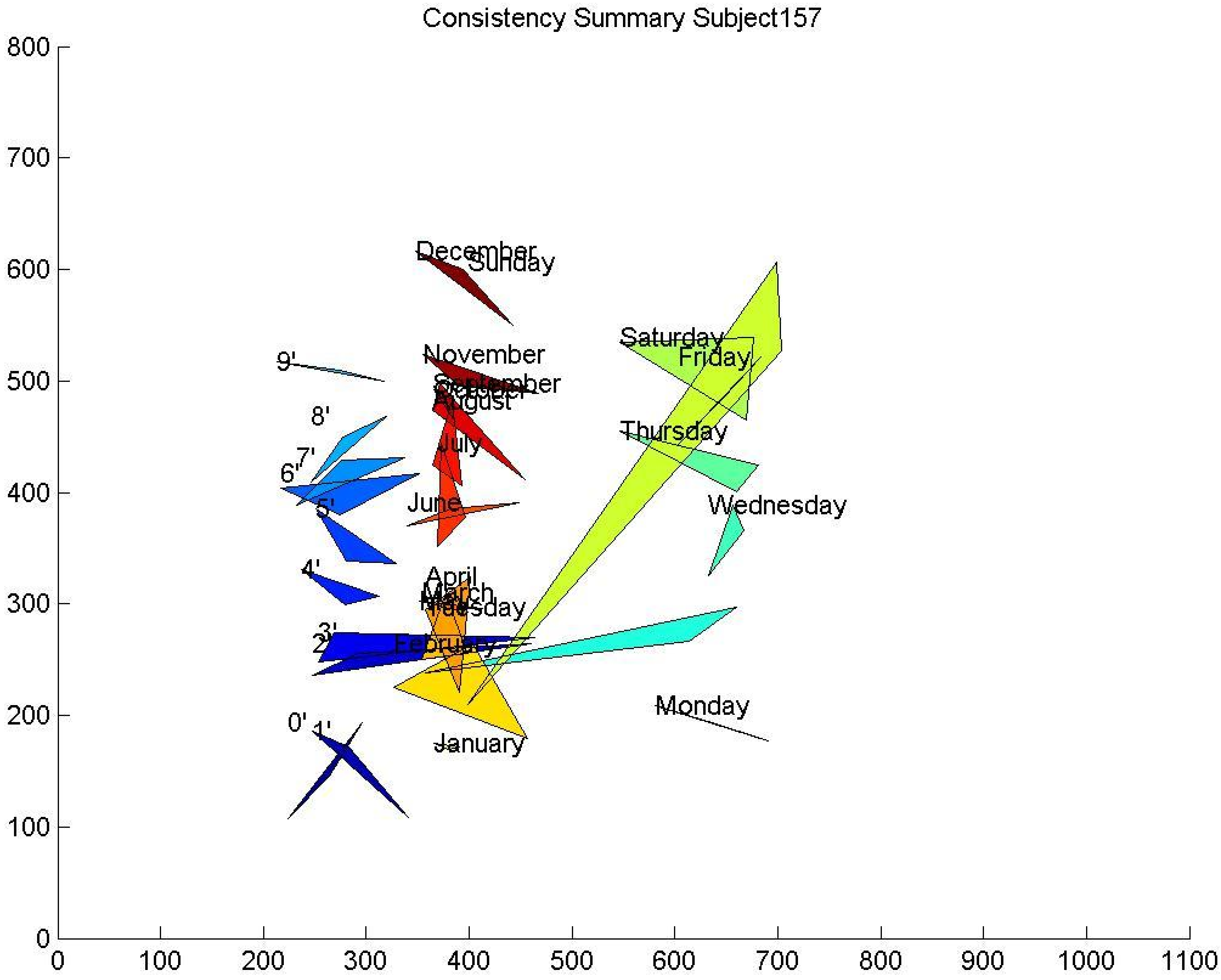
Consistency Summary Subject157
December
Sunday
Saturday
Friday
November
September
August
July
Thursday
June
Wednesday
April
March
February
Monday
January
0'
1'
2'
3'
4'
5'
6'
7'
8'
9'
0
100
200
300
400
500
600
700
800
900
1000
1100

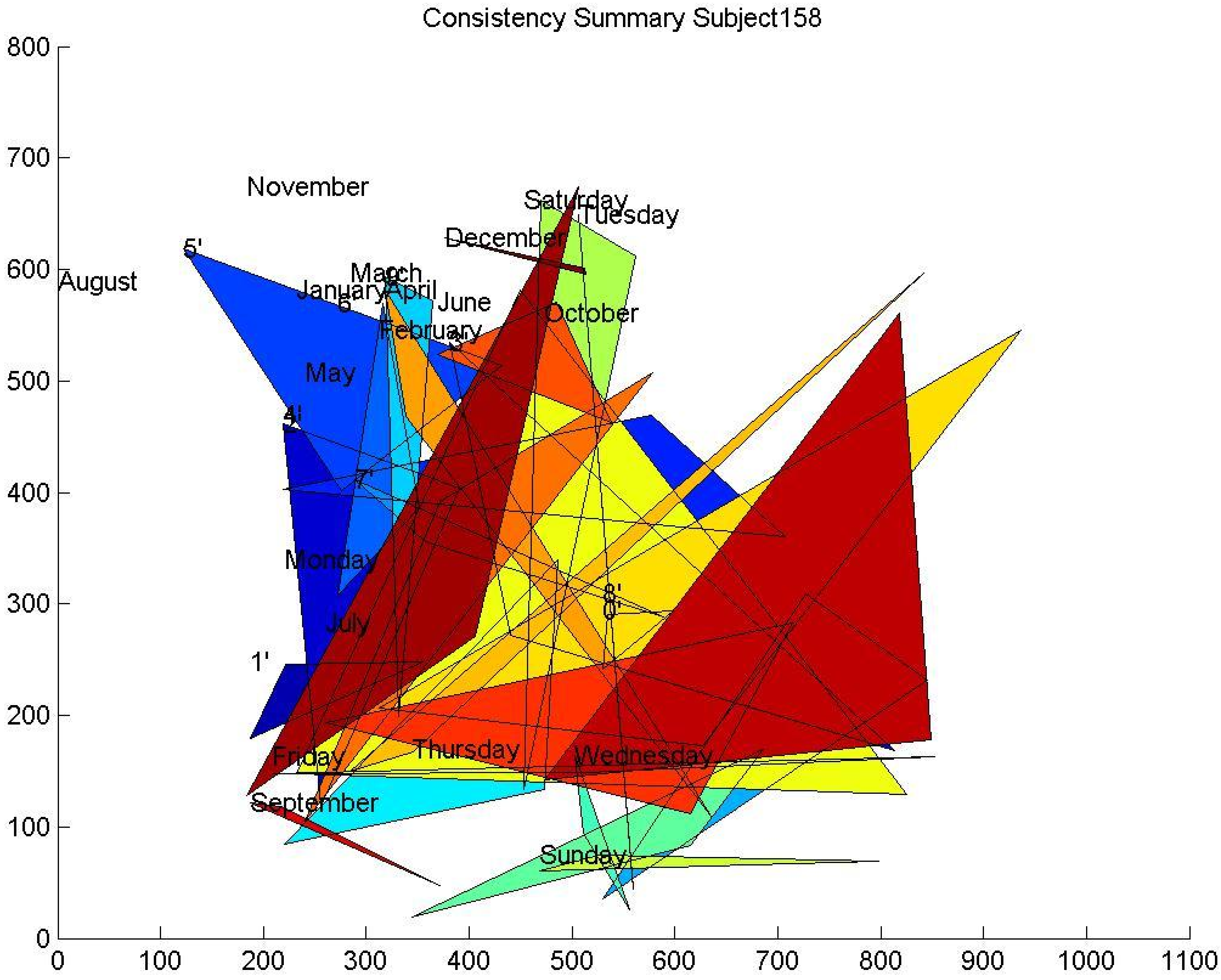
Consistency Summary Subject158
November
Saturday
Tuesday
December
5'
August
March
January
April
6'
June
October
February
3'
May
4'
7'
Monday
8'
0'
July
1'
Thursday
Friday
Wednesday
September
Sunday
0
100
200
300
400
500
600
700
800
900
1000
1100
0
100
200
300
400
500
600
700
800

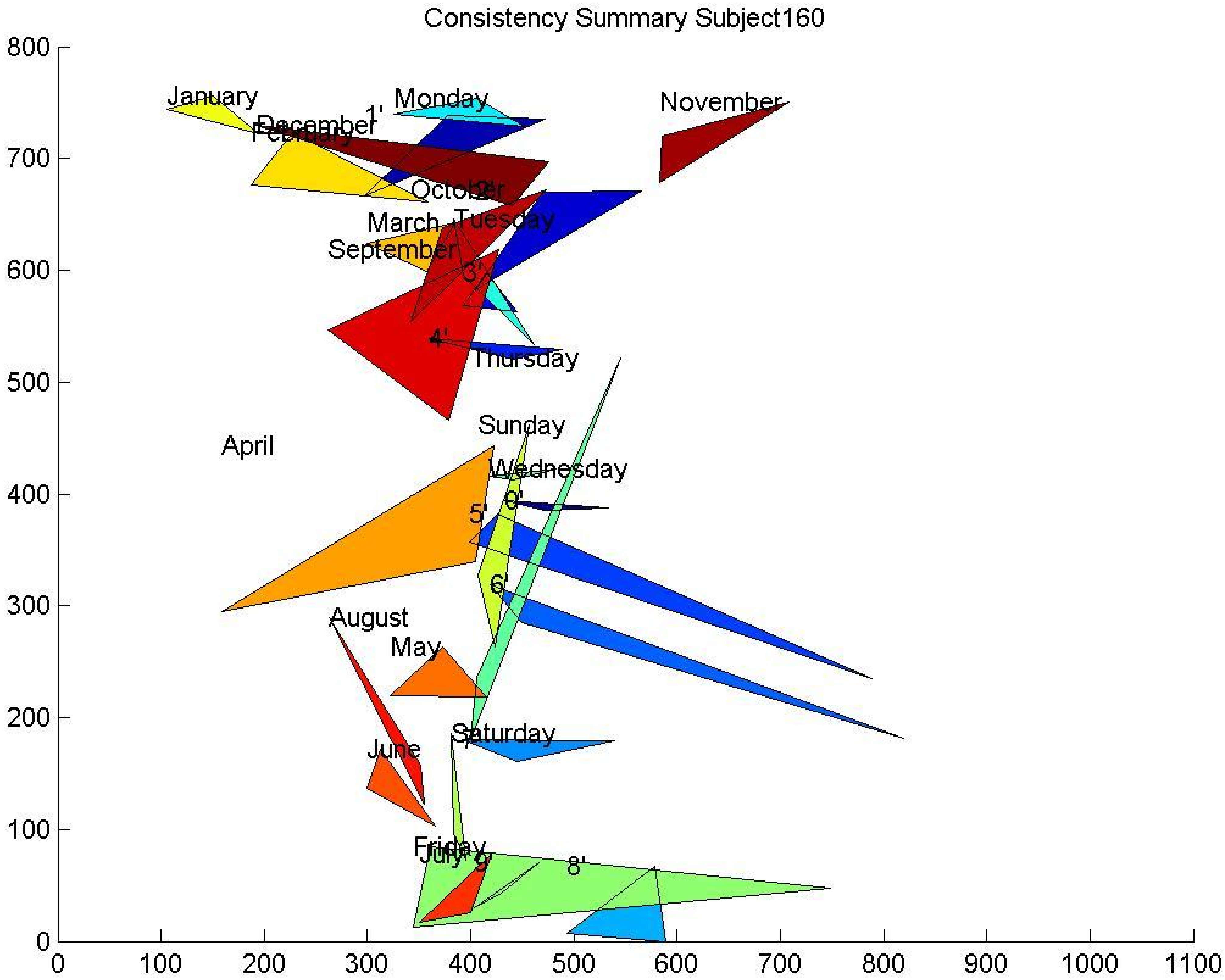

Consistency Summary Subject160
January
December
February
Monday
1'
November
October
2'
March
Tuesday
September
3'
4'
Thursday
Sunday
April
Wednesday
0'
5'
6'
August
May
Saturday
June
Friday
July
9'
8'
0
100
200
300
400
500
600
700
800
900
1000
1100

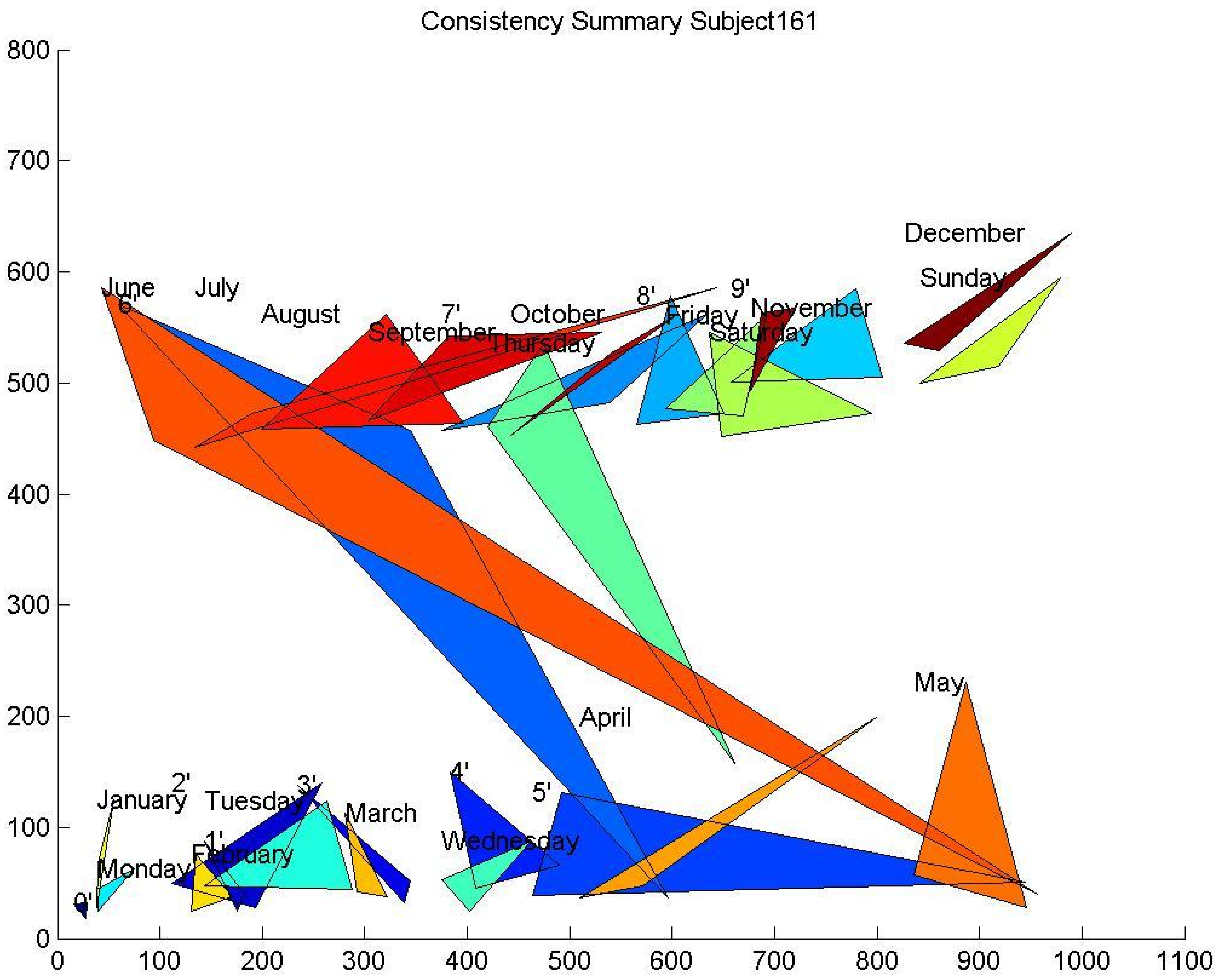

Consistency Summary Subject161
800
700
600
500
400
300
200
100
0
0
100
200
300
400
500
600
700
800
900
1000
1100
December
Sunday
June
6'
July
August
7'
September
October
Thursday
8'
Friday
9'
November
Saturday
May
April
2'
January
Tuesday
3'
March
4'
5'
1'
February
Wednesday
Monday
0'

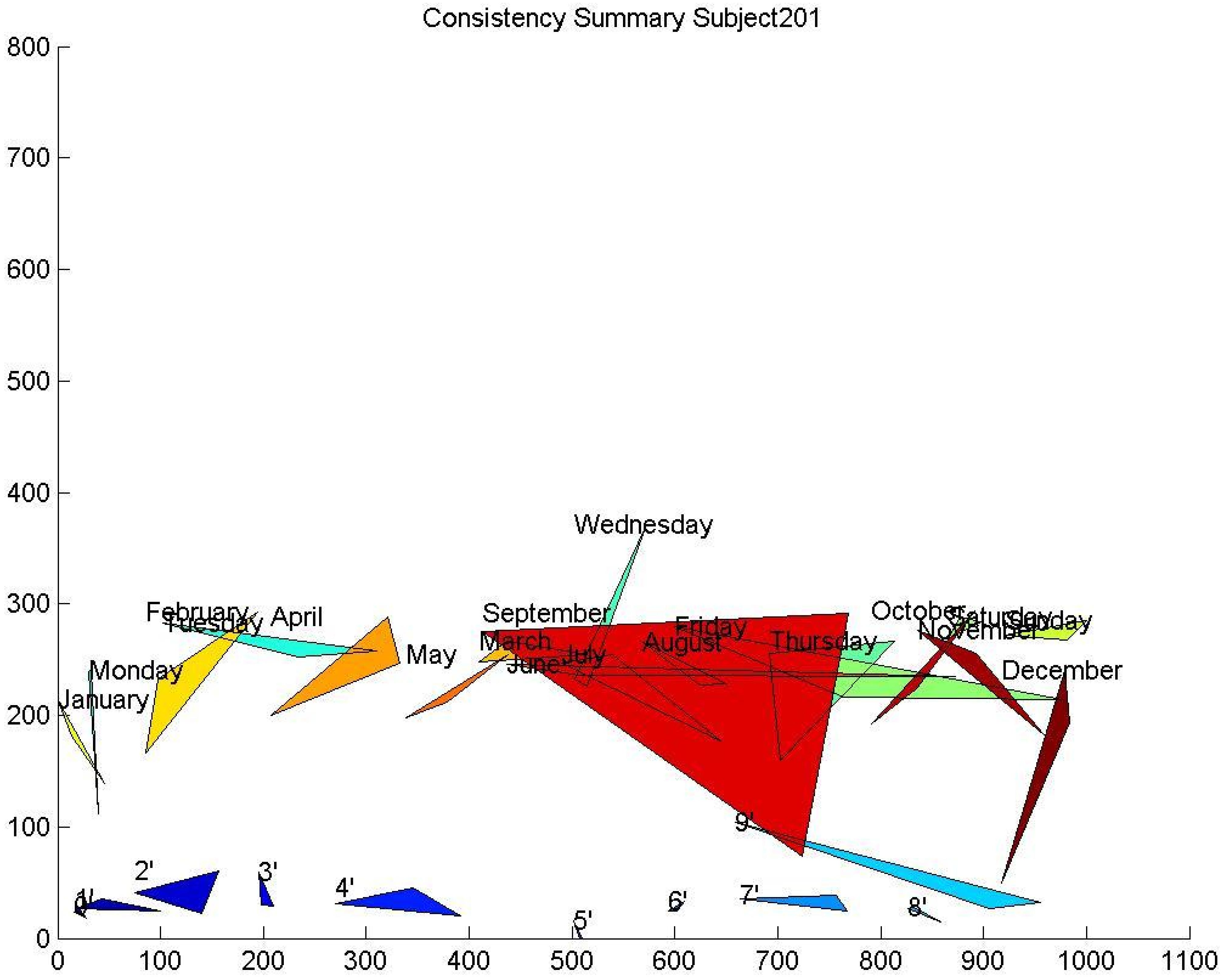

Consistency Summary Subject201
800
700
600
500
400
300
200
100
0
0
100
200
300
400
500
600
700
800
900
1000
1100
Wednesday
February
Tuesday
April
September
October
Saturday
Sunday
November
Friday
March
August
Thursday
May
July
June
Monday
December
January
9'
2'
3'
4'
1'
0'
5'
6'
7'
8'

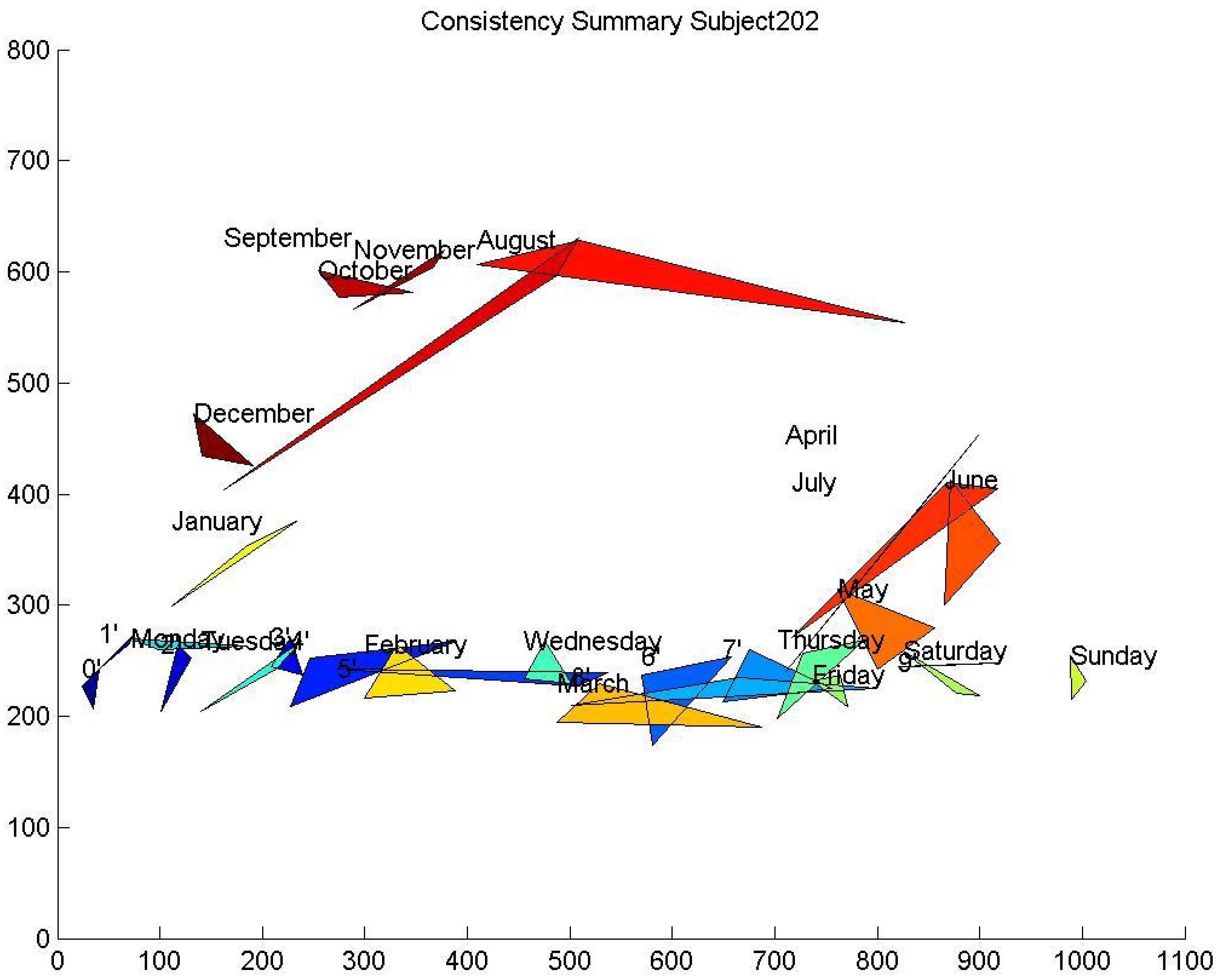
Consistency Summary Subject202
September
November
August
October
December
April
July
June
January
May
1'
0'
Monday
2
Tuesday
3'
4'
5'
February
Wednesday
6'
8'
March
7'
Thursday
Friday
9'
Saturday
Sunday
0
100
200
300
400
500
600
700
800
900
1000
1100

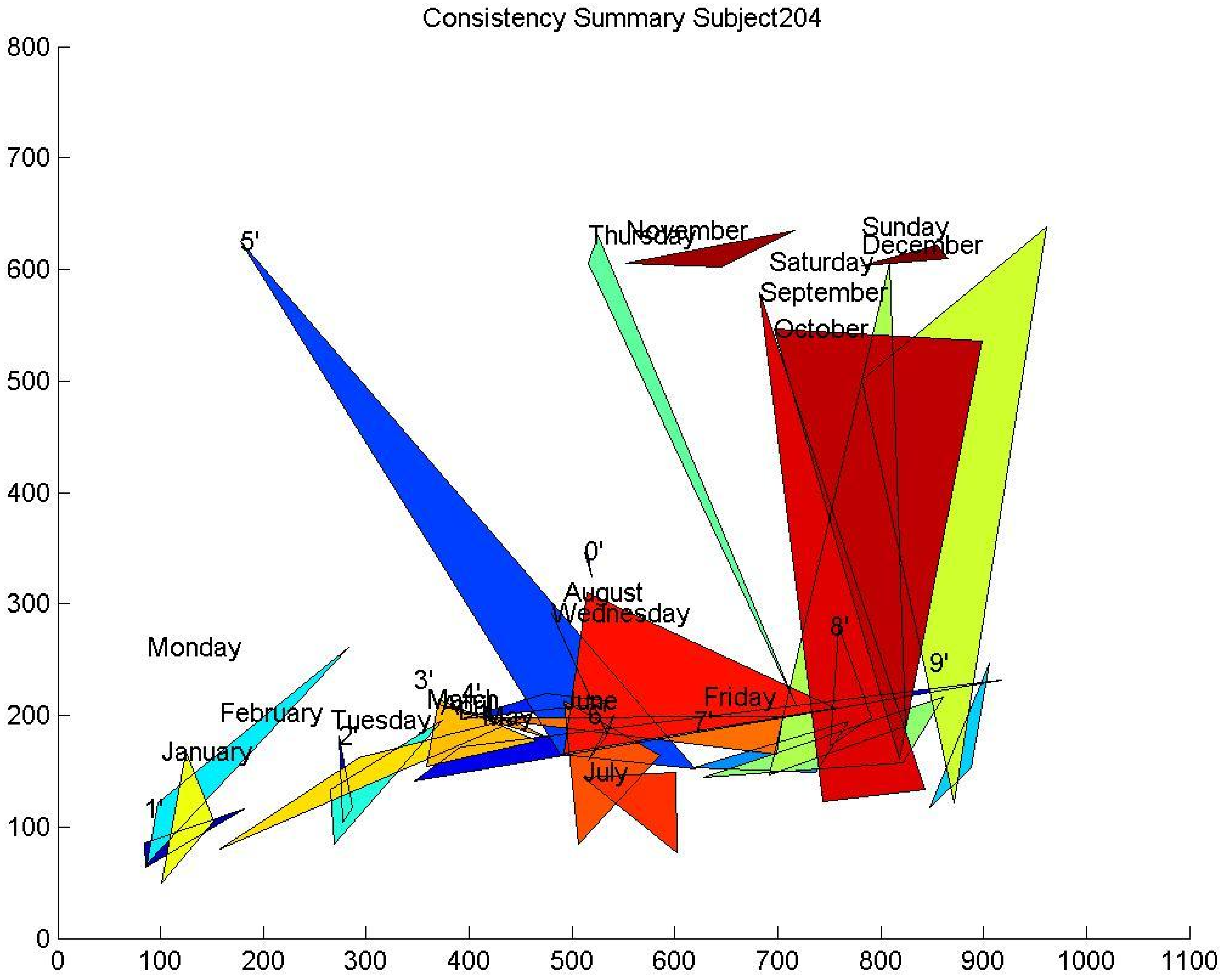

Consistency Summary Subject204
800
700
600
500
400
300
200
100
0
0
100
200
300
400
500
600
700
800
900
1000
1100
5'
Thursday
November
Sunday
December
Saturday
September
October
0'
August
Wednesday
Monday
8'
9'
3'
4'
Friday
February
Tuesday
March
May
June
6'
7'
2'
January
July
1'

Consistency Summary Subject205

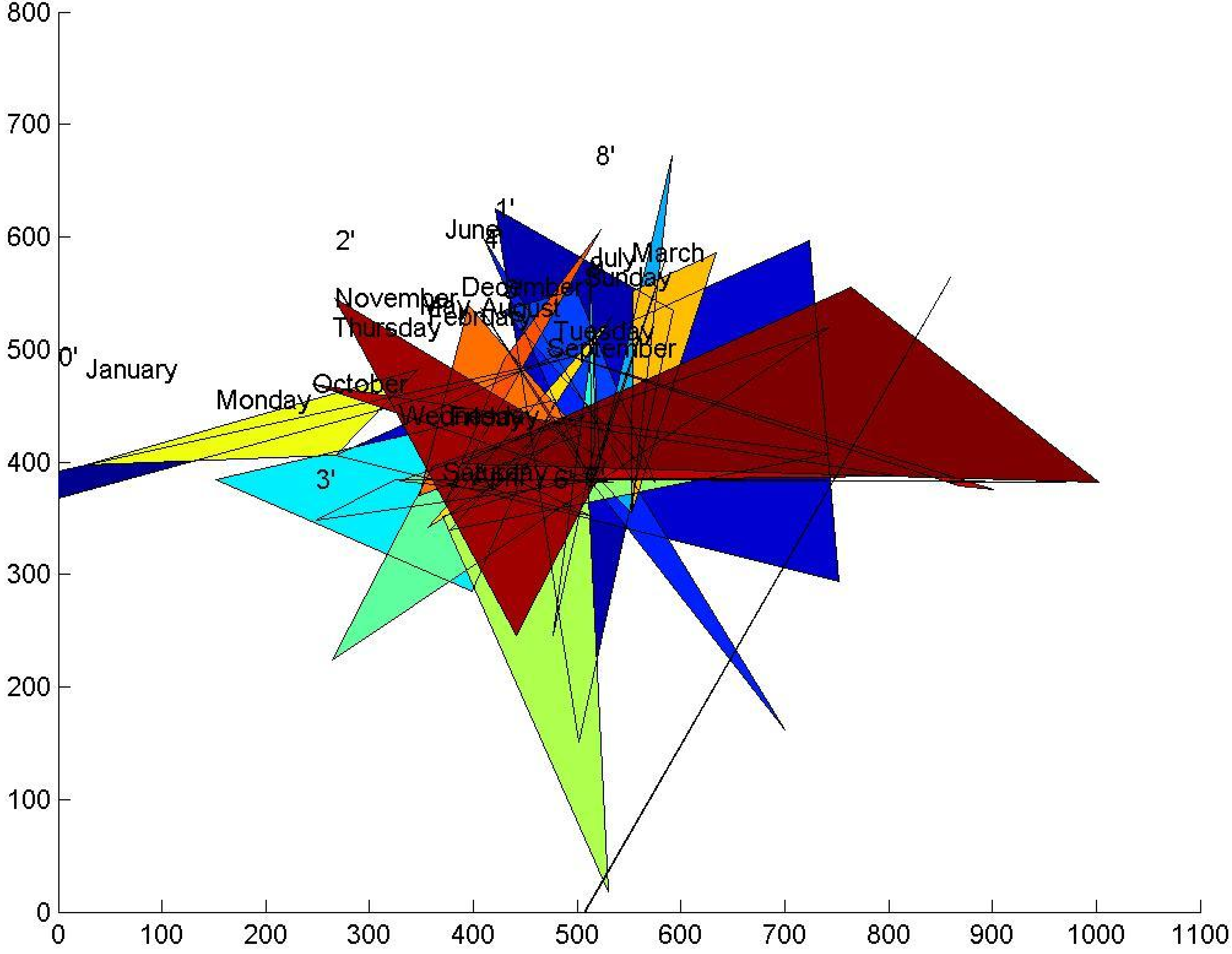

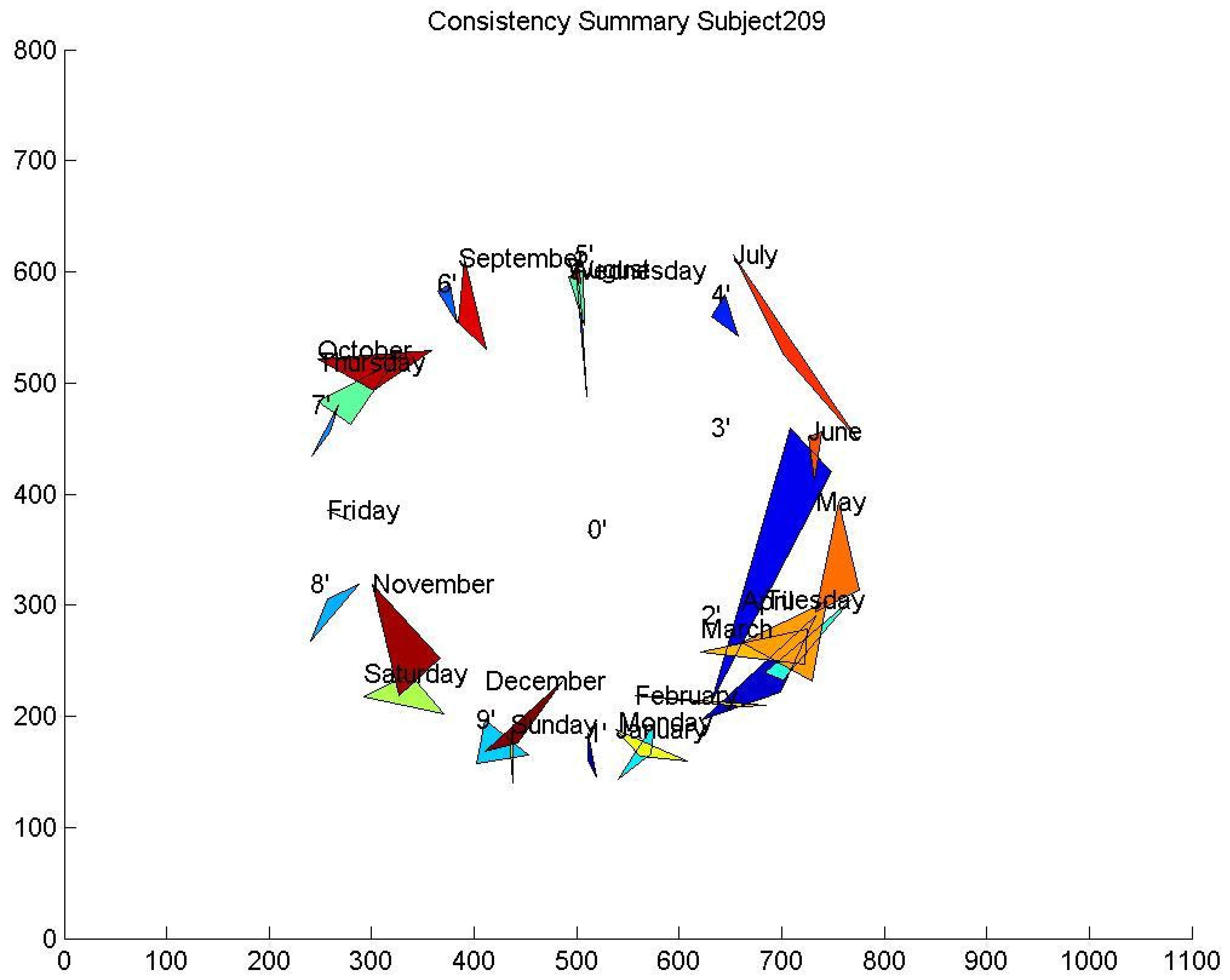

Consistency Summary Subject209
September
July
June
May
October
Thursday
Friday
November
Saturday
December
Sunday
February
Monday
January
March
0'
2'
3'
4'
5'
6'
7'
8'
9'
0
100
200
300
400
500
600
700
800
900
1000
1100

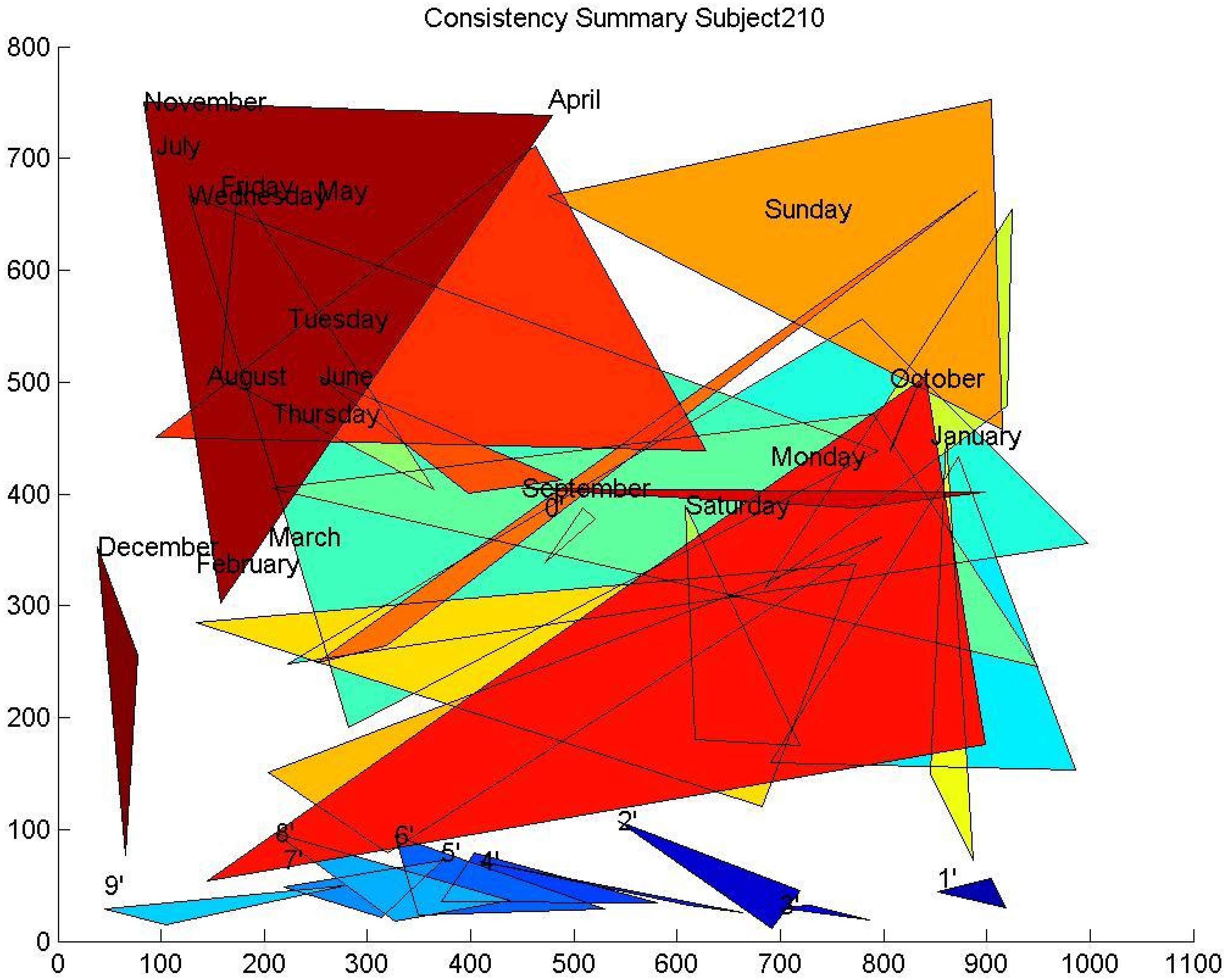

Consistency Summary Subject210
November
April
July
Friday
Wednesday
May
Sunday
Tuesday
August
June
October
Thursday
January
Monday
September
0'
Saturday
December
March
February
2'
8'
6'
5'
4'
7'
1'
9'
3'
0
100
200
300
400
500
600
700
800
900
1000
1100

Consistency Summary Subject214

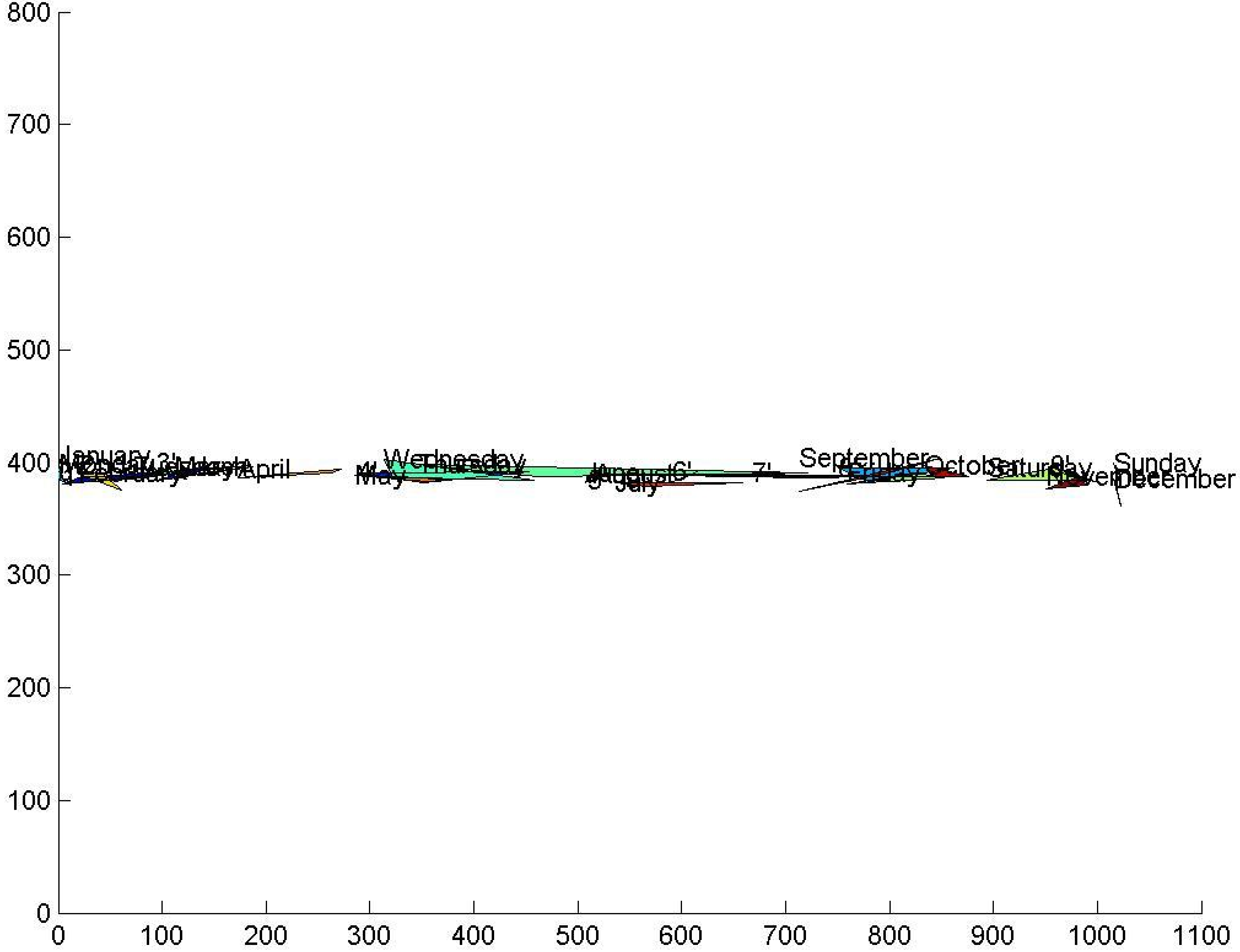

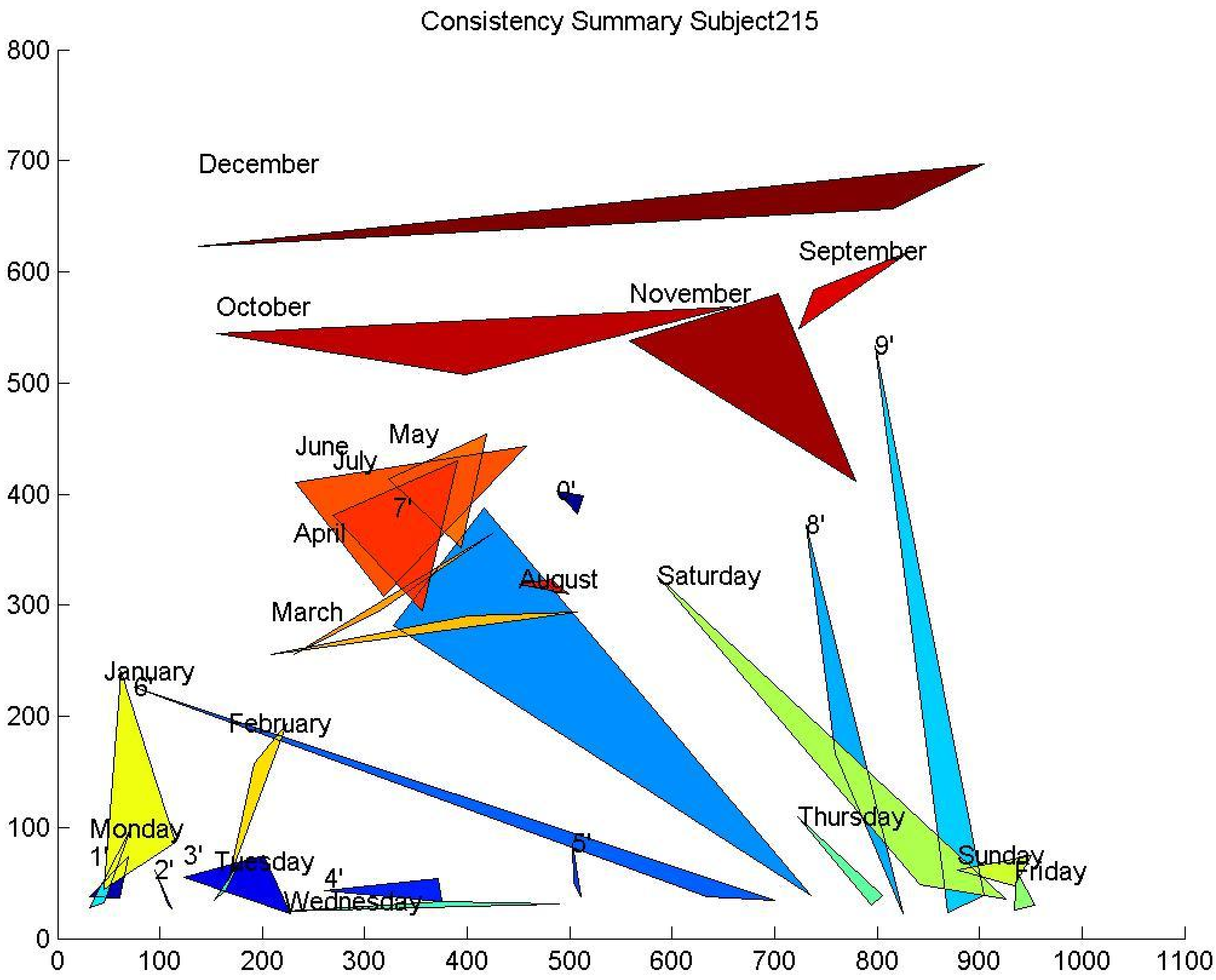
Consistency Summary Subject215
December
September
November
October
9'
May
June
July
7'
0'
April
8'
August
Saturday
March
January
6'
February
Thursday
Monday
5'
1'
3'
Tuesday
Sunday
2'
Friday
4'
Wednesday
0
100
200
300
400
500
600
700
800
900
1000
1100

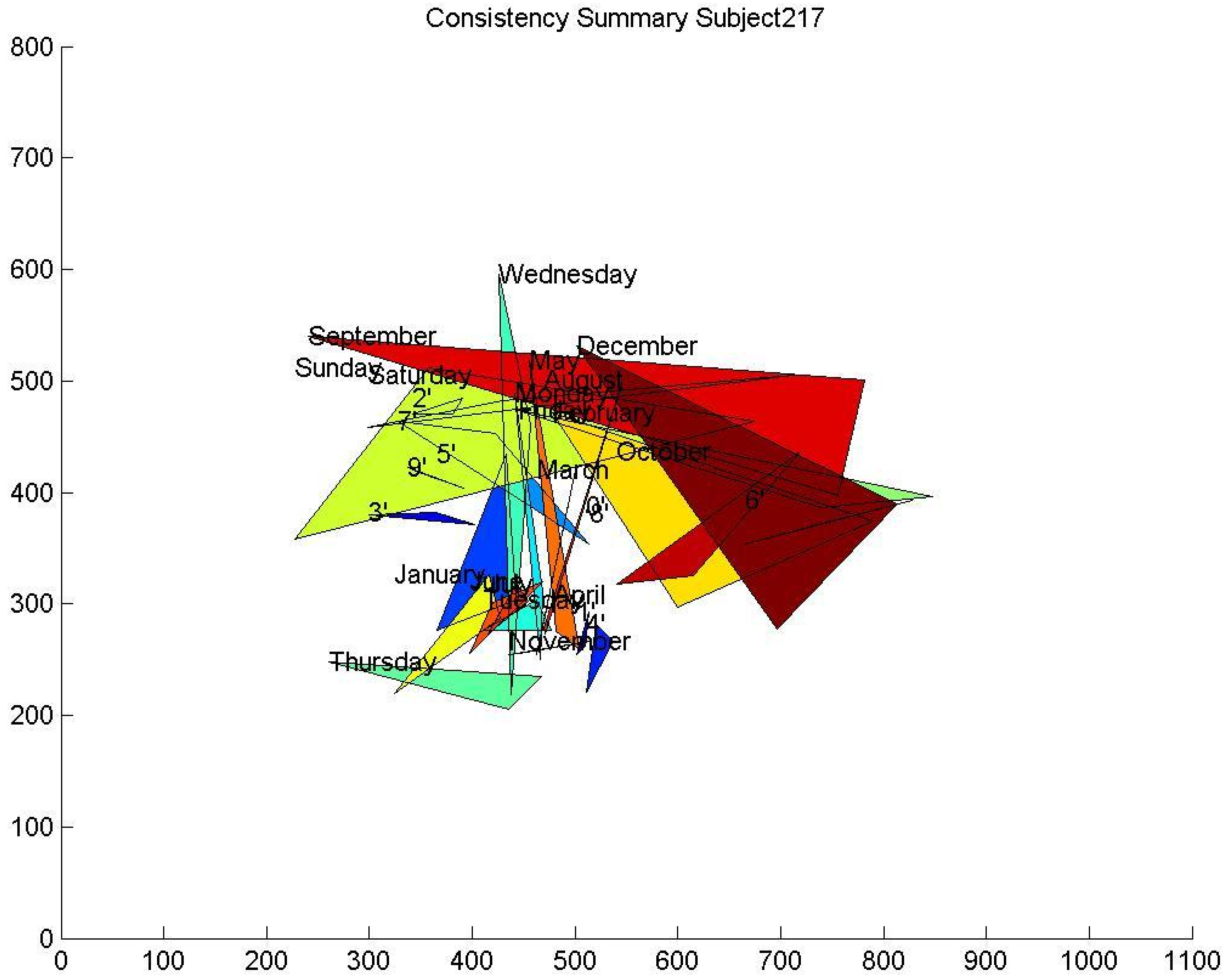
Consistency Summary Subject217
Wednesday
September
December
May
Sunday
Saturday
August
2'
Monday
February
7'
October
5'
9'
March
6'
3'
January
April
4'
November
Thursday
0
100
200
300
400
500
600
700
800
900
1000
1100

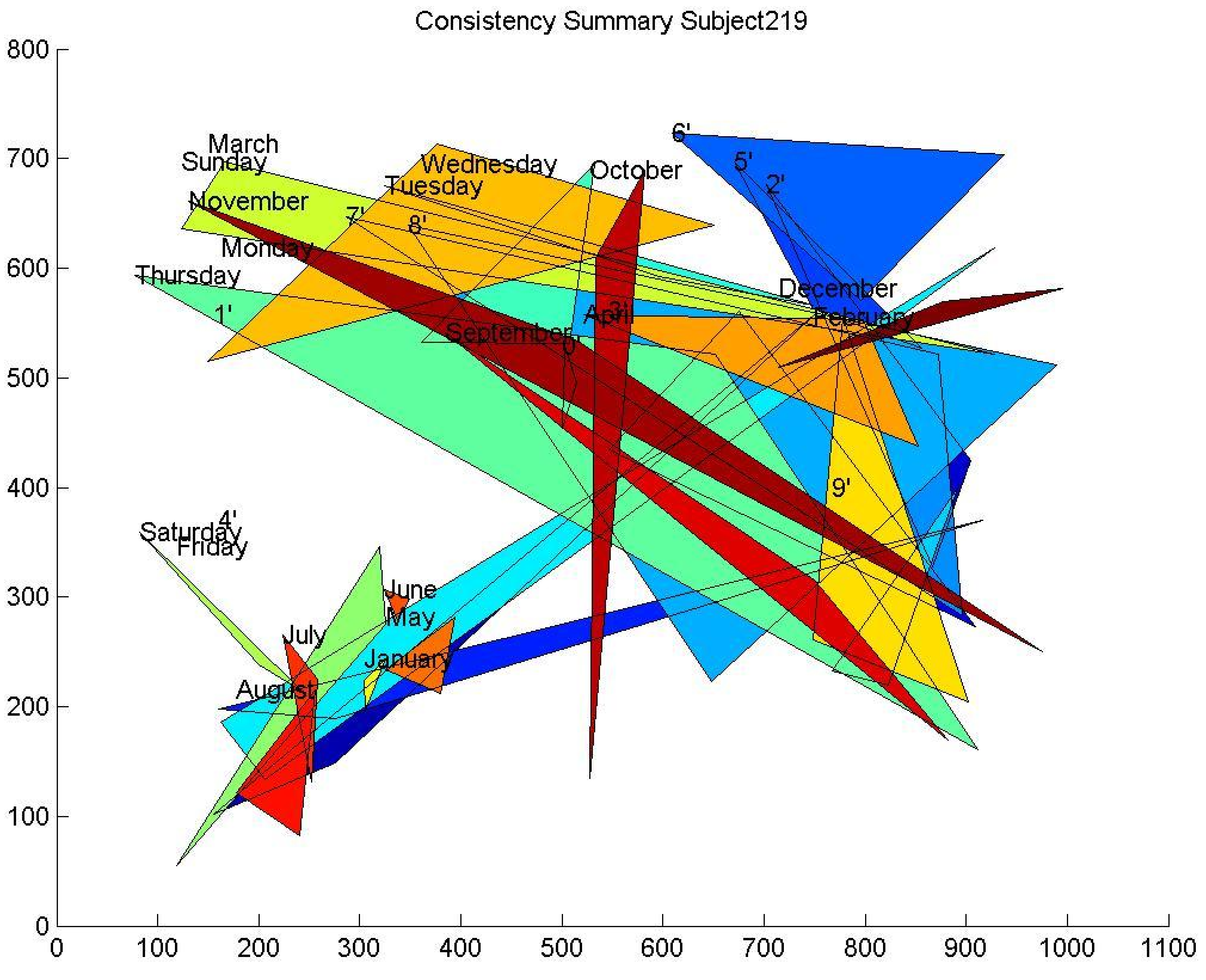

Consistency Summary Subject219
March
Sunday
Wednesday
Tuesday
October
6'
5'
2'
November
7'
8'
Monday
Thursday
1'
December
April
3'
February
September
0'
9'
4'
Saturday
Friday
June
May
July
January
August
0
100
200
300
400
500
600
700
800
900
1000
1100

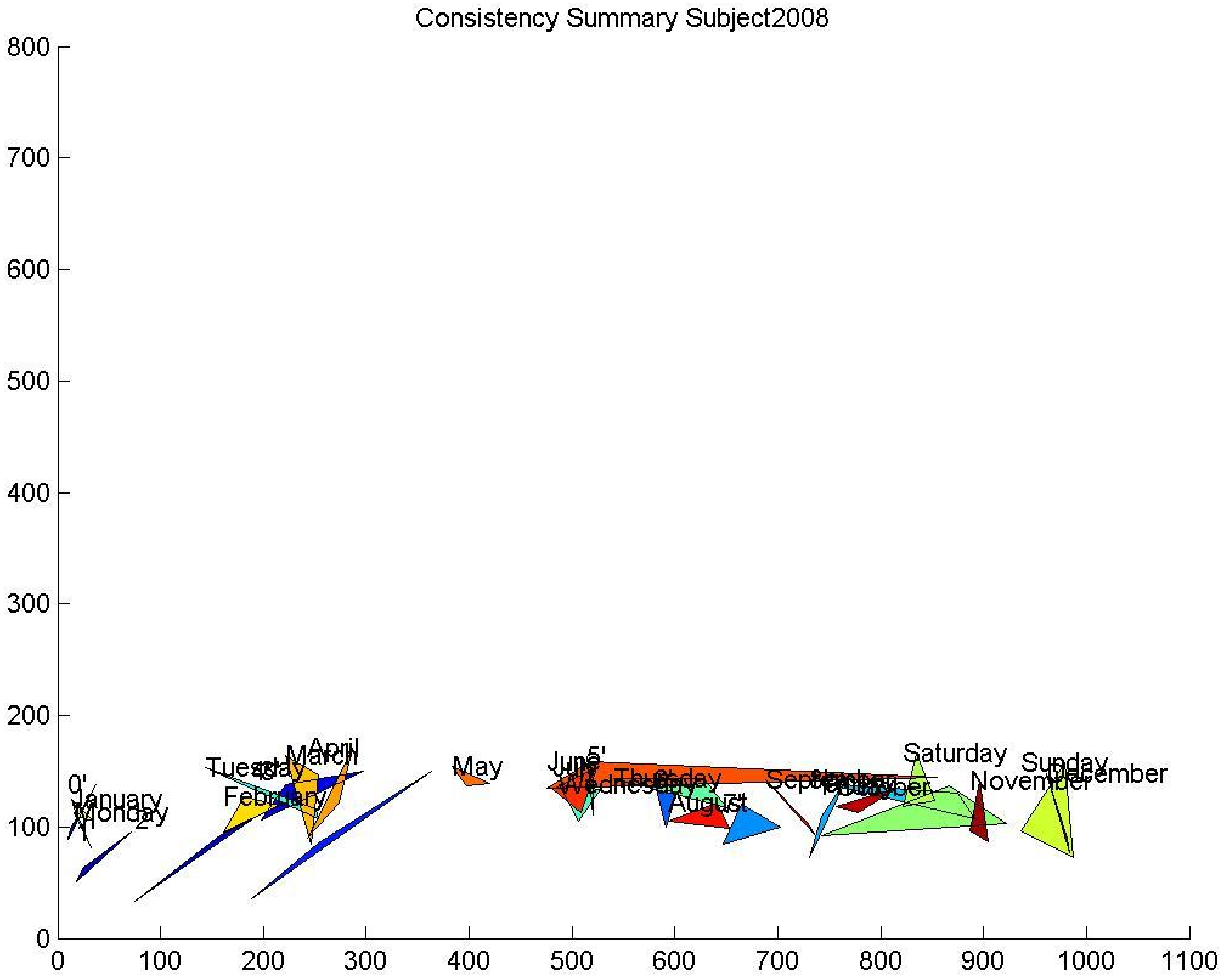

Consistency Summary Subject2008
800
700
600
500
400
300
200
100
0
0
100
200
300
400
500
600
700
800
900
1000
1100
January
Monday
February
Tuesday
March
April
May
June
July
Wednesday
Thursday
August
September
Saturday
November
Sunday
December

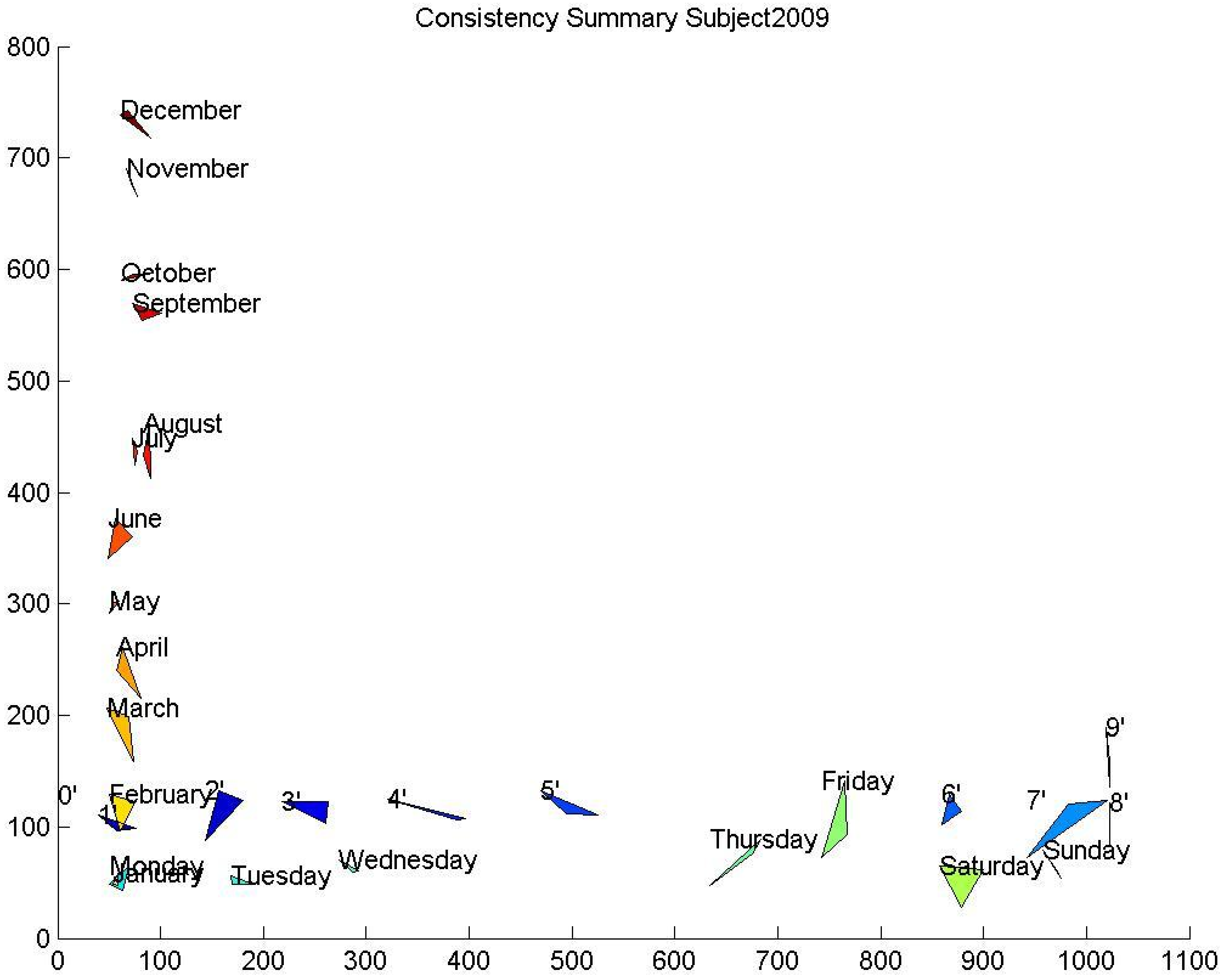

Consistency Summary Subject2009
December
November
October
September
August
July
June
May
April
March
February
January
Monday
Tuesday
Wednesday
Thursday
Friday
Saturday
Sunday
0'
1'
2'
3'
4'
5'
6'
7'
8'
9'
0
100
200
300
400
500
600
700
800
900
1000
1100

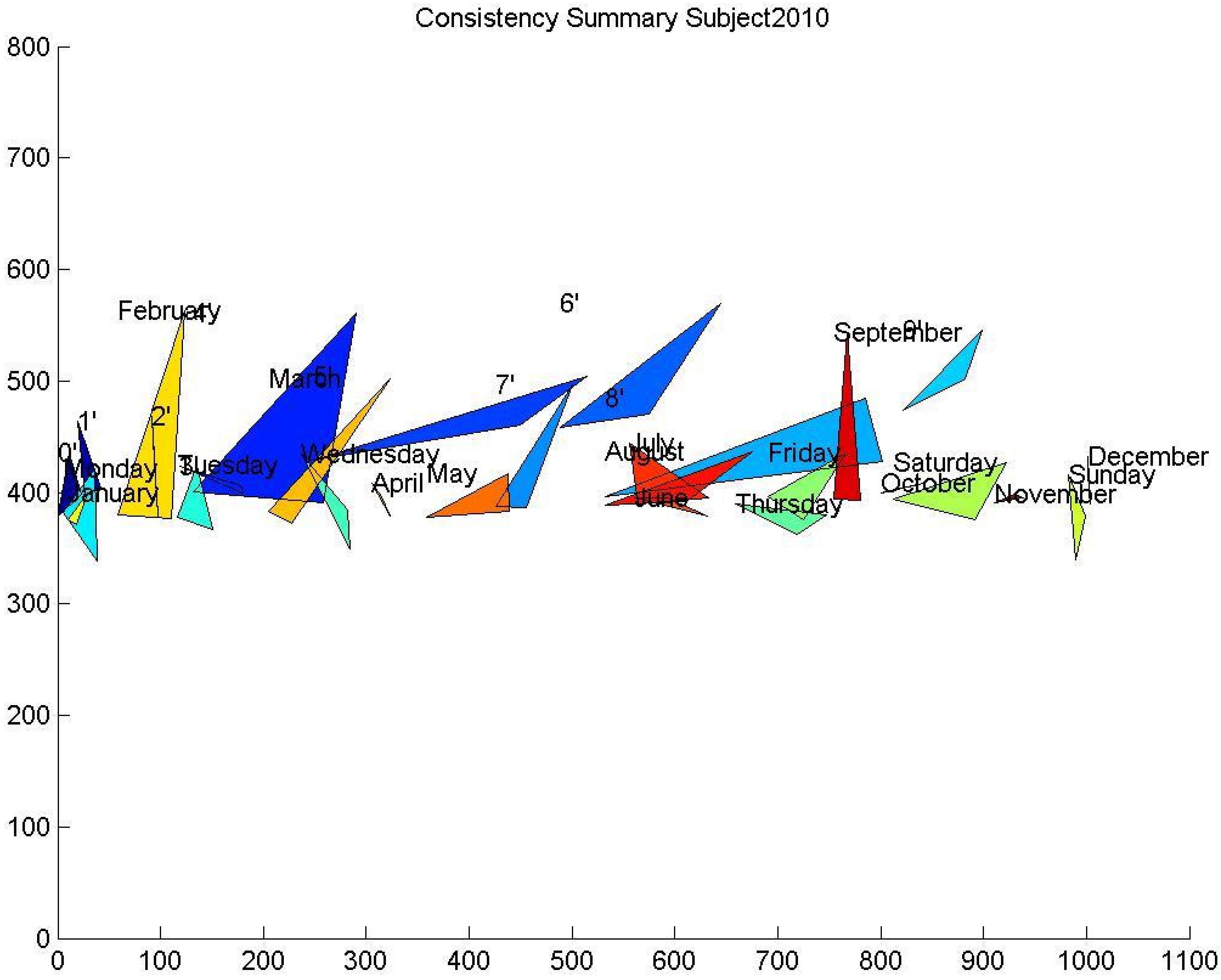

Consistency Summary Subject2010
800
700
600
500
400
300
200
100
0
0
100
200
300
400
500
600
700
800
900
1000
1100
February
March
Wednesday
Tuesday
Monday
January
April
May
6'
7'
8'
July
August
June
Thursday
Friday
September
Saturday
October
November
December
Sunday
0'
1'
2'

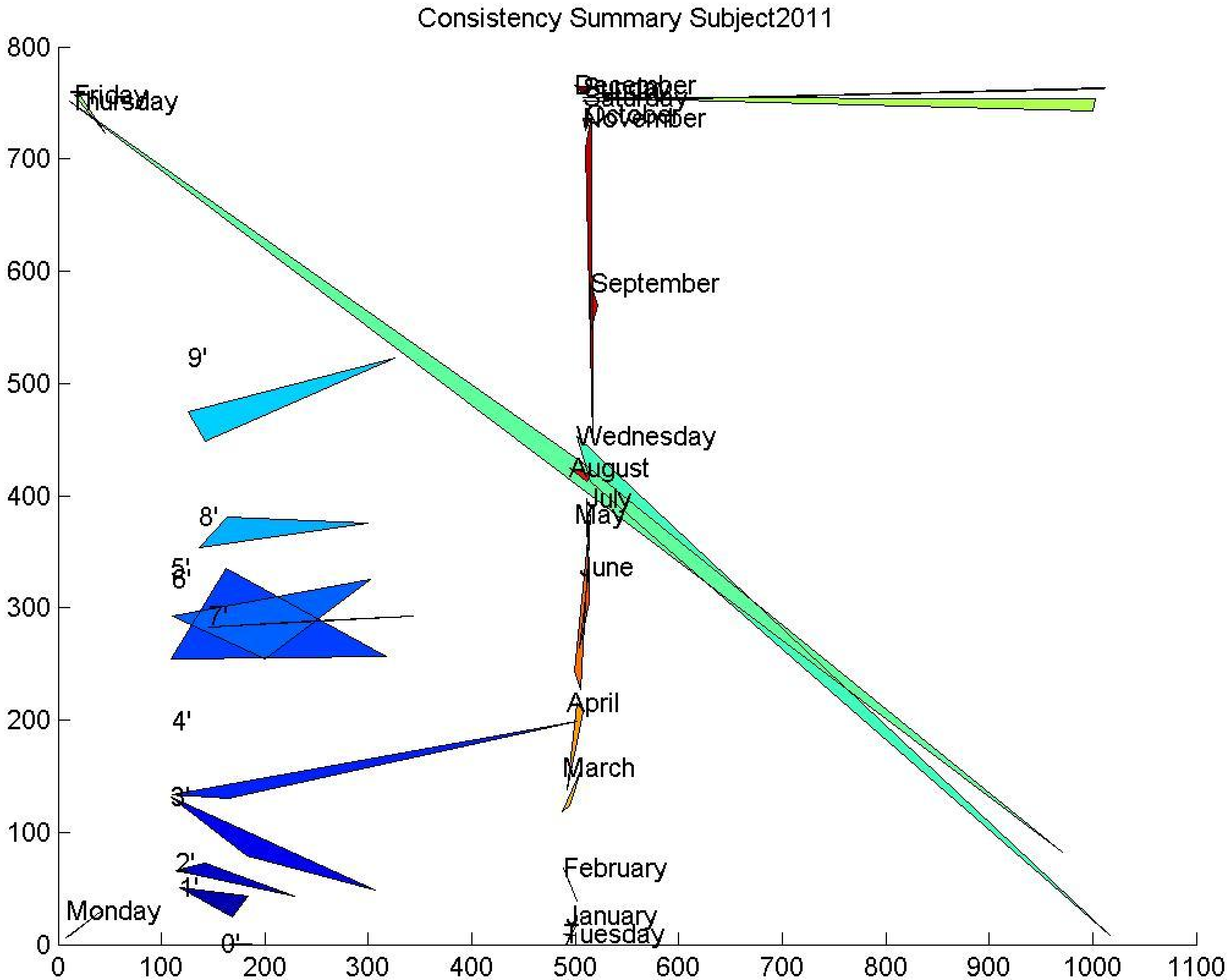
Consistency Summary Subject2011
Friday
Thursday
December
Saturday
October
November
September
9'
Wednesday
August
July
May
8'
5'
6'
June
7'
April
4'
March
3'
2'
February
1'
Monday
January
Tuesday
0'
0
100
200
300
400
500
600
700
800
900
1000
1100

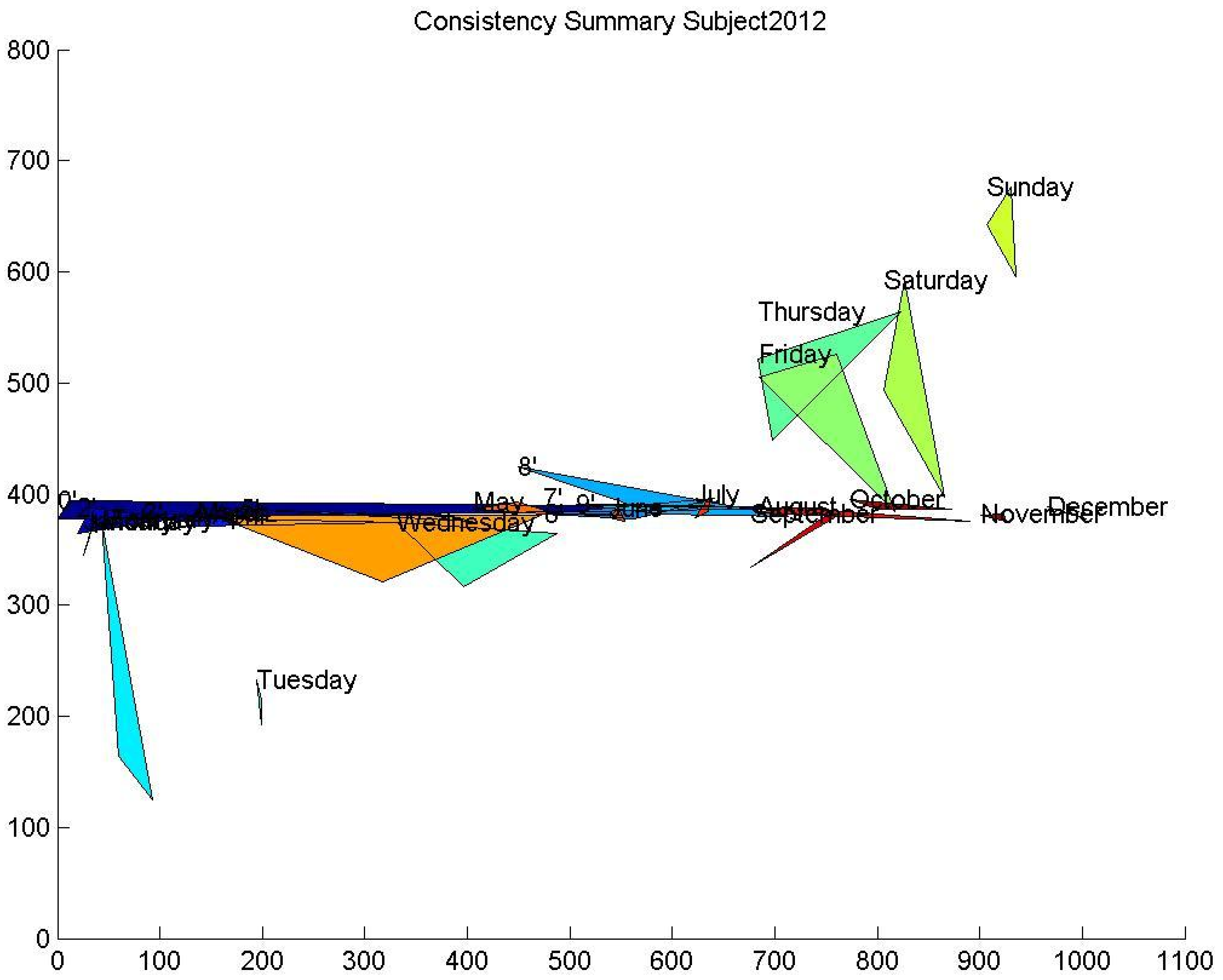

Consistency Summary Subject2012
800
700
600
500
400
300
200
100
0
0
100
200
300
400
500
600
700
800
900
1000
1100
Sunday
Saturday
Thursday
Friday
8'
May
7'
June
July
August
October
September
November
December
Wednesday
Tuesday

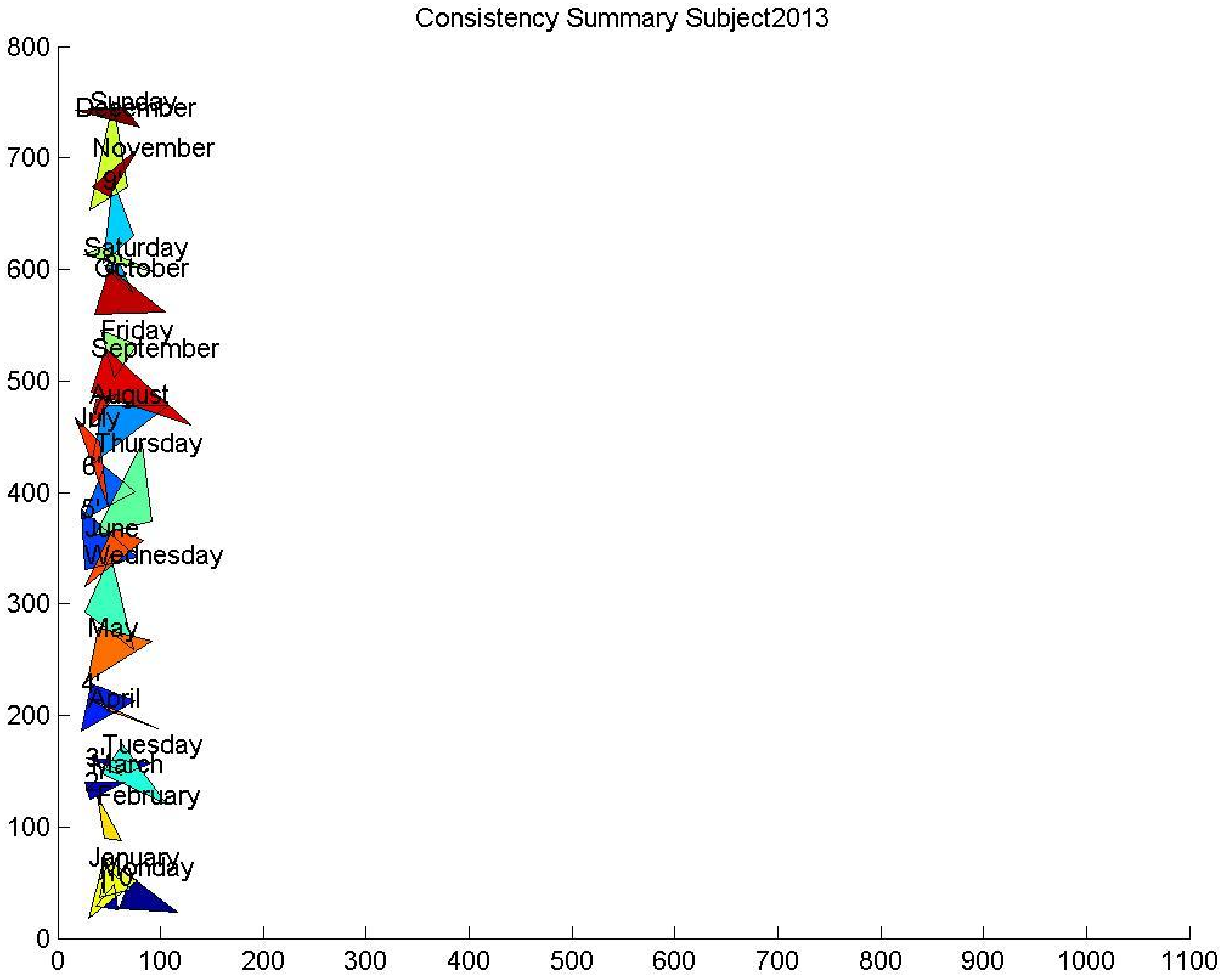

Consistency Summary Subject2013
800
700
600
500
400
300
200
100
0
0
100
200
300
400
500
600
700
800
900
1000
1100
Sunday
December
November
Saturday
October
Friday
September
August
July
Thursday
June
Wednesday
May
April
Tuesday
March
February
January
Monday

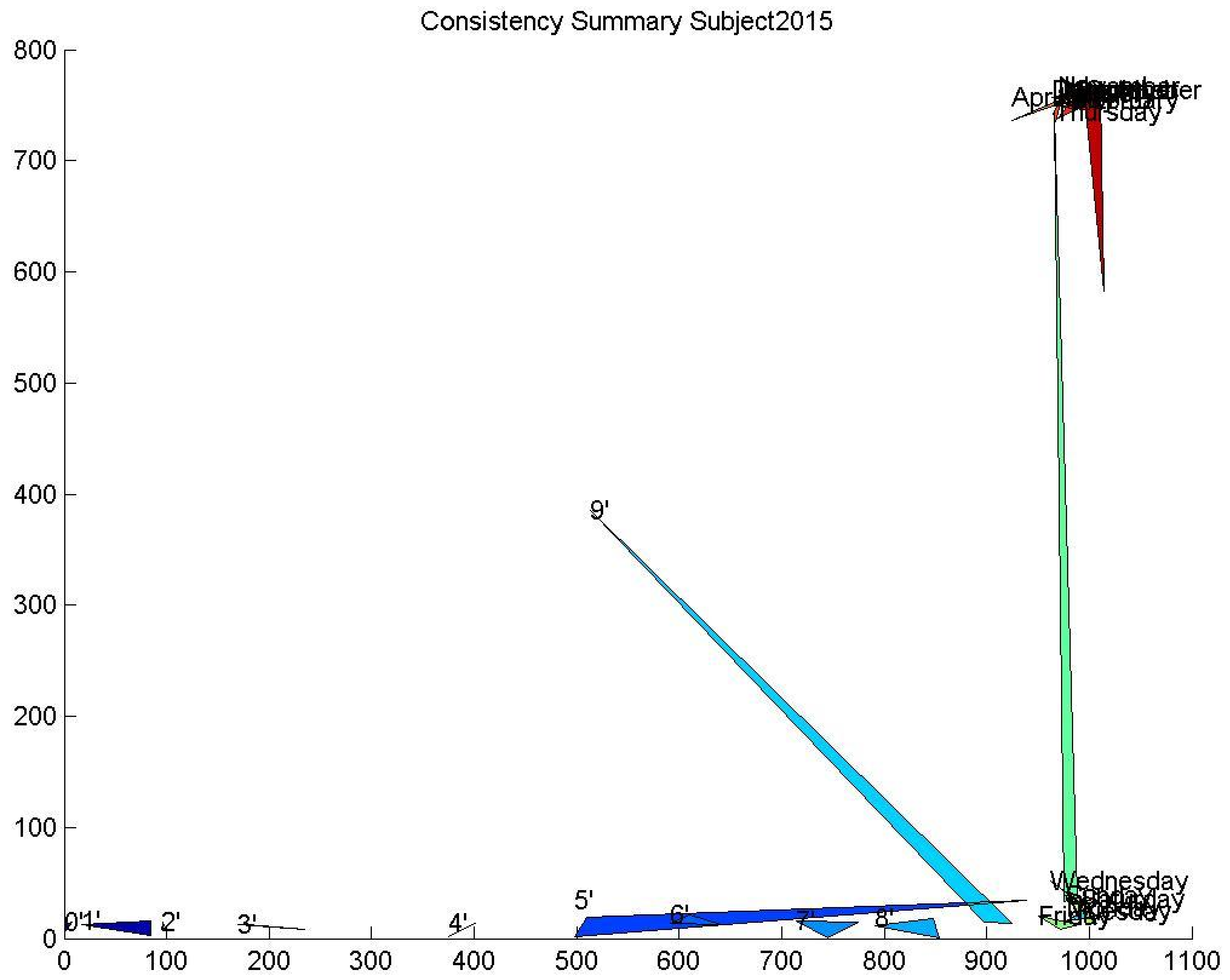
Consistency Summary Subject2015
800
700
600
500
400
300
200
100
0
0
100
200
300
400
500
600
700
800
900
1000
1100
9'
0'1'
2'
3'
4'
5'
6'
7'
8'
Thursday
Wednesday
Friday

Consistency Summary Subject2016

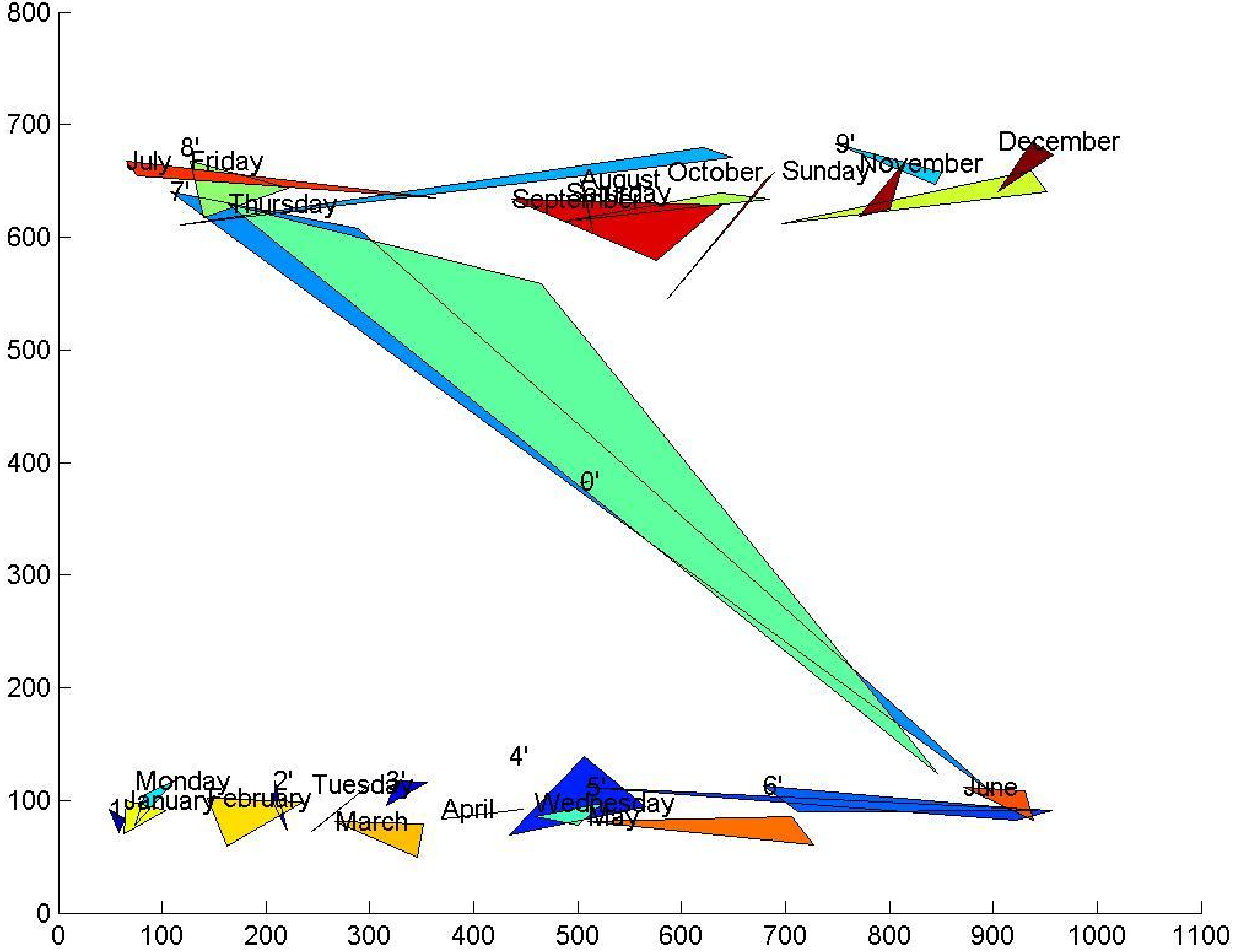

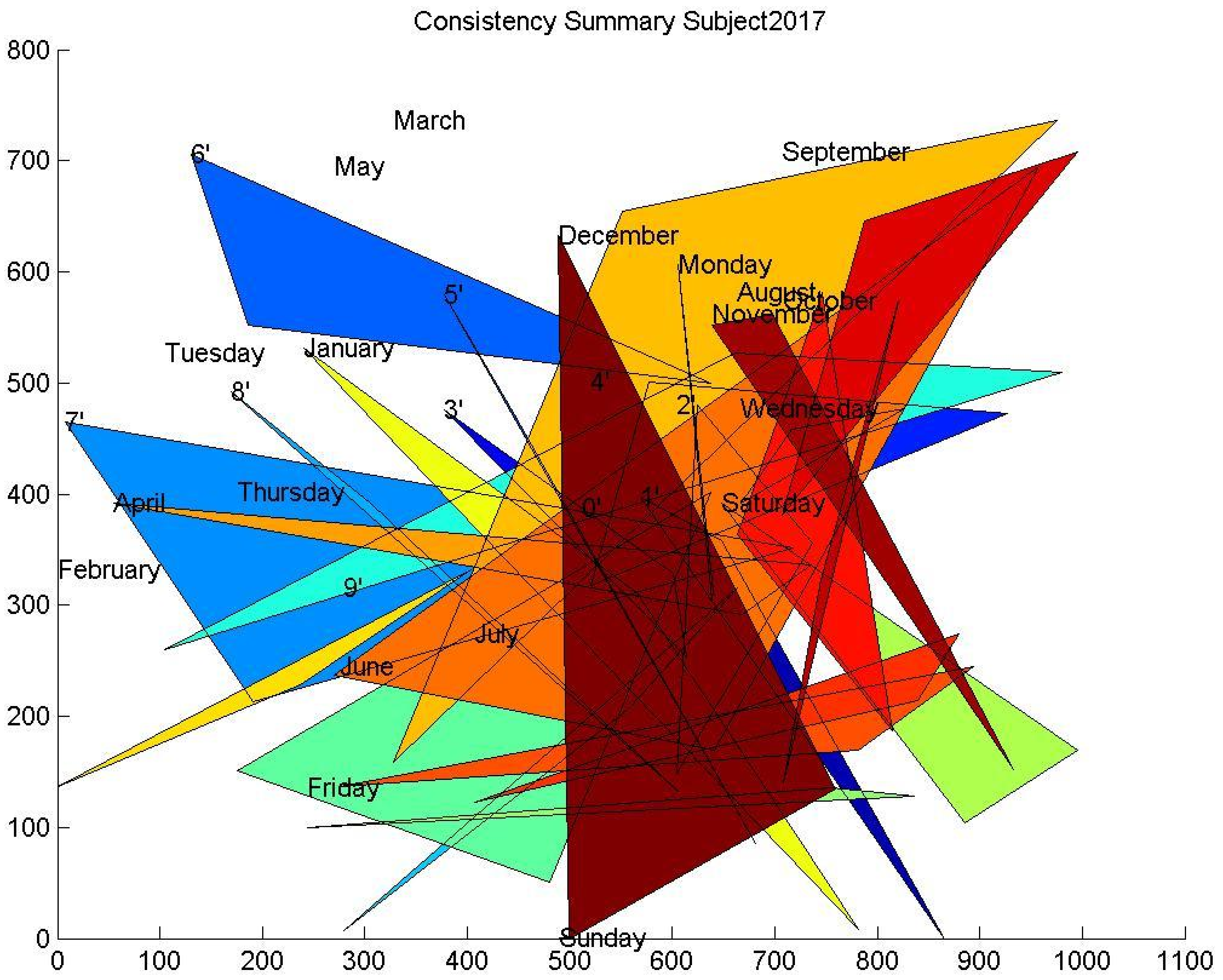

Consistency Summary Subject2017
800
700
600
500
400
300
200
100
0
0
100
200
300
400
500
600
700
800
900
1000
1100
March
May
September
6'
December
Monday
5'
August
October
November
Tuesday
January
8'
4'
2'
3'
Wednesday
7'
Thursday
April
0'
1'
Saturday
February
9'
July
June
Friday
Sunday

Consistency Summary Subject2018

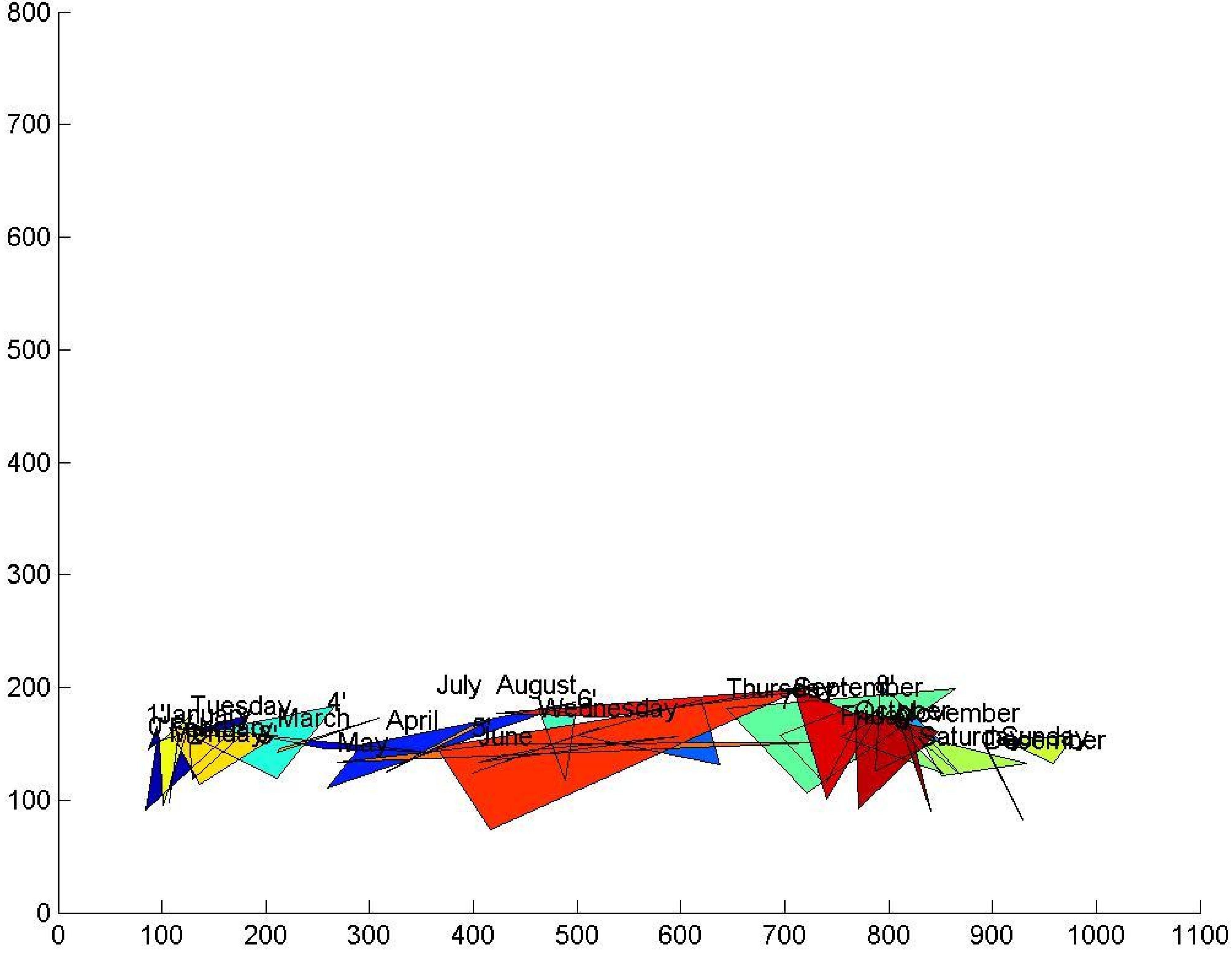

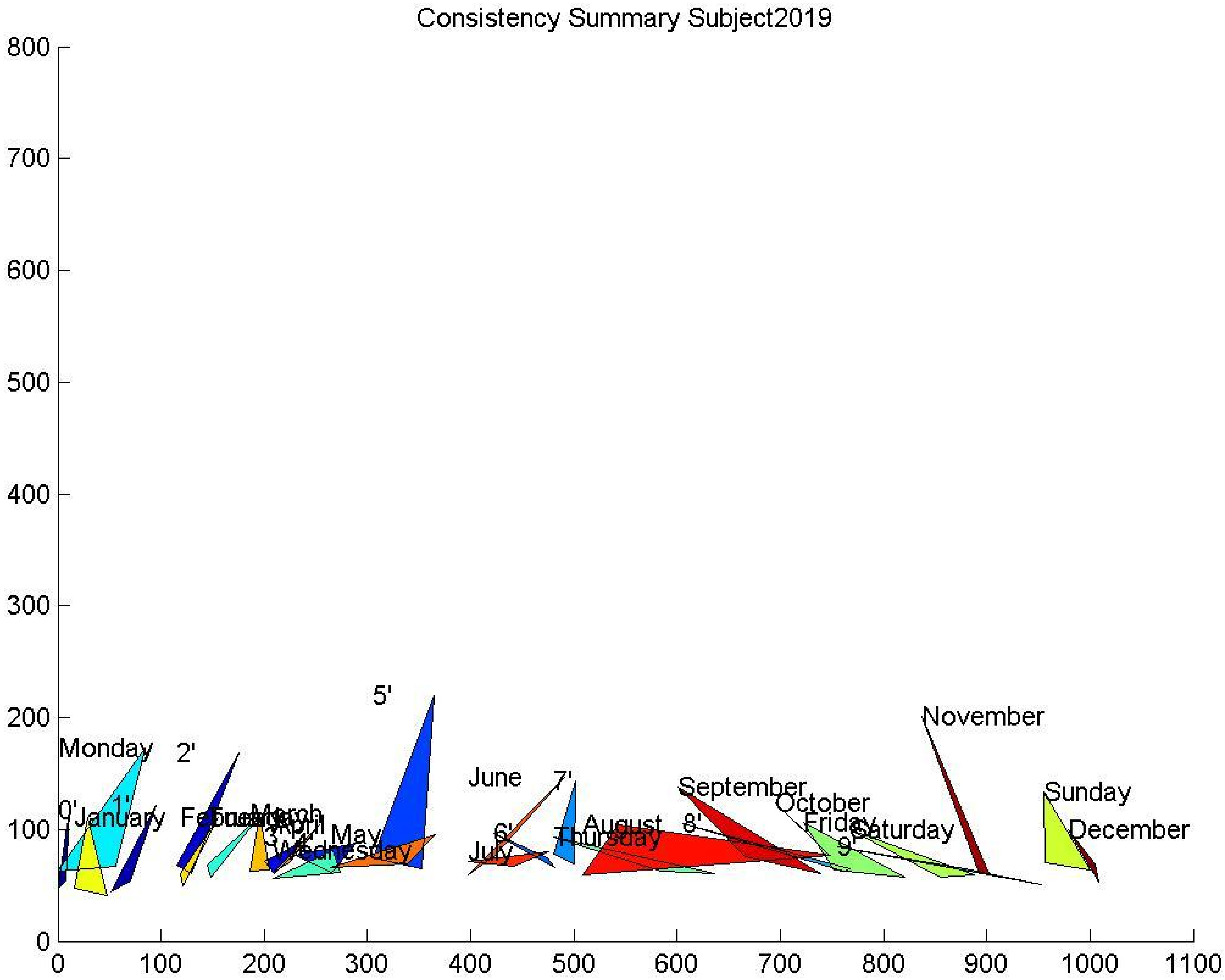
Consistency Summary Subject2019
800
700
600
500
400
300
200
100
0
0
100
200
300
400
500
600
700
800
900
1000
1100
Monday
January
0'
1'
2'
5'
March
April
May
Wednesday
June
July
6'
7'
Thursday
August
8'
September
October
Friday
Saturday
9'
November
Sunday
December

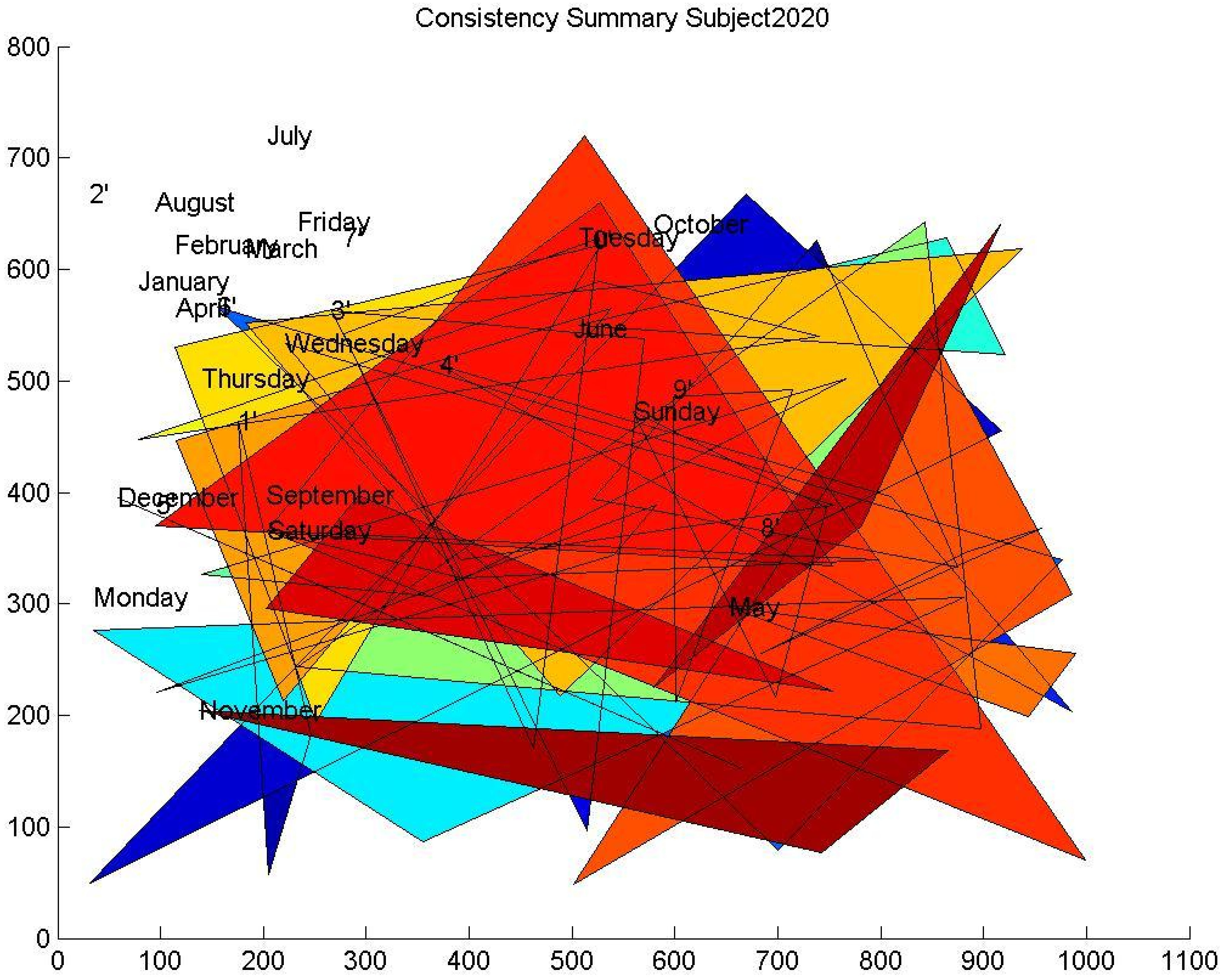
Consistency Summary Subject2020
July
2'
August
Friday
October
Tuesday
February
March
January
April
3'
June
Wednesday
4'
Thursday
9'
Sunday
1'
September
Saturday
8'
Monday
May
November
0
100
200
300
400
500
600
700
800
900
1000
1100

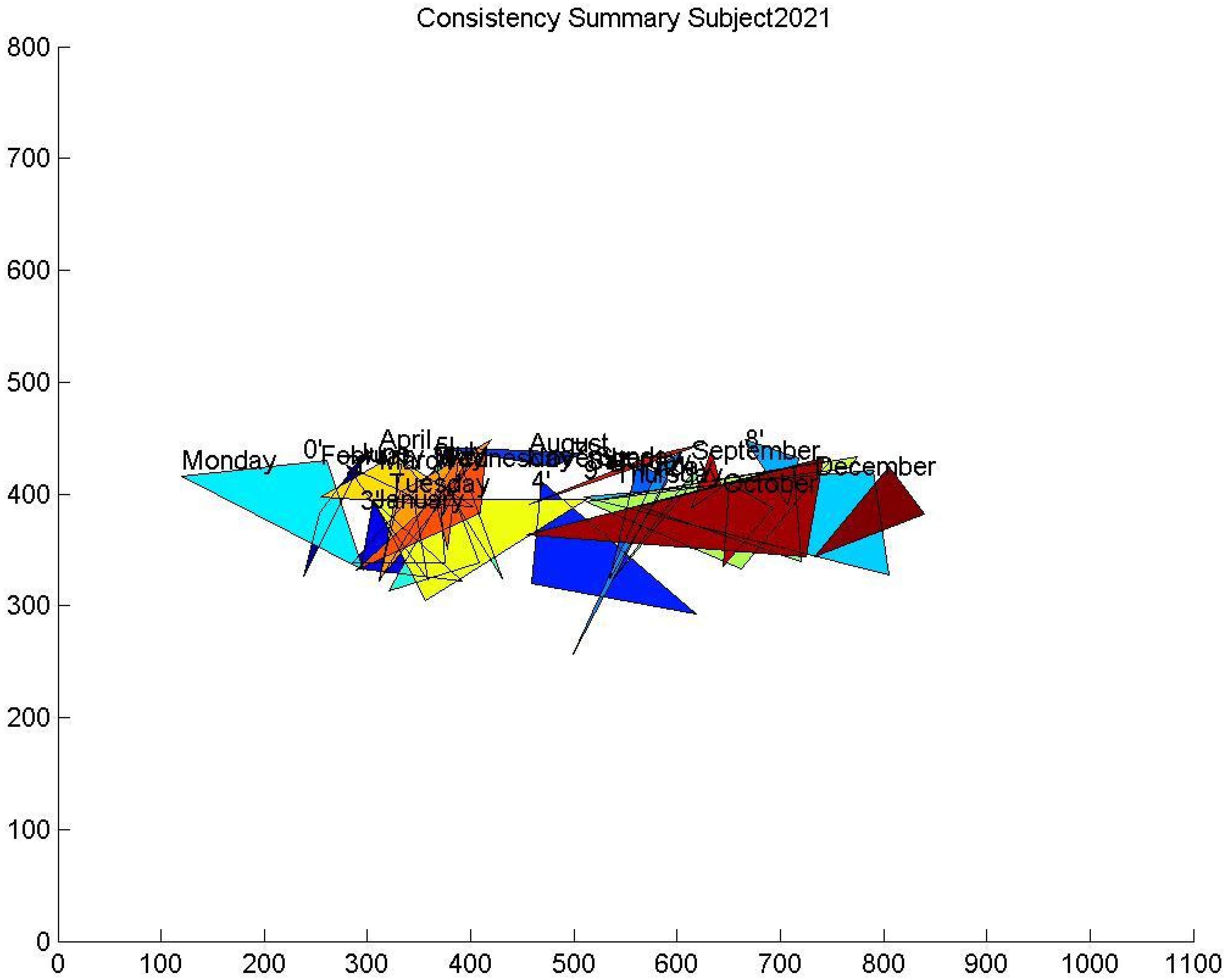
Consistency Summary Subject2021
800
700
600
500
400
300
200
100
0
0
100
200
300
400
500
600
700
800
900
1000
1100
Monday
April
August
September
December
October
Tuesday
Thursday
8'
4'
0'

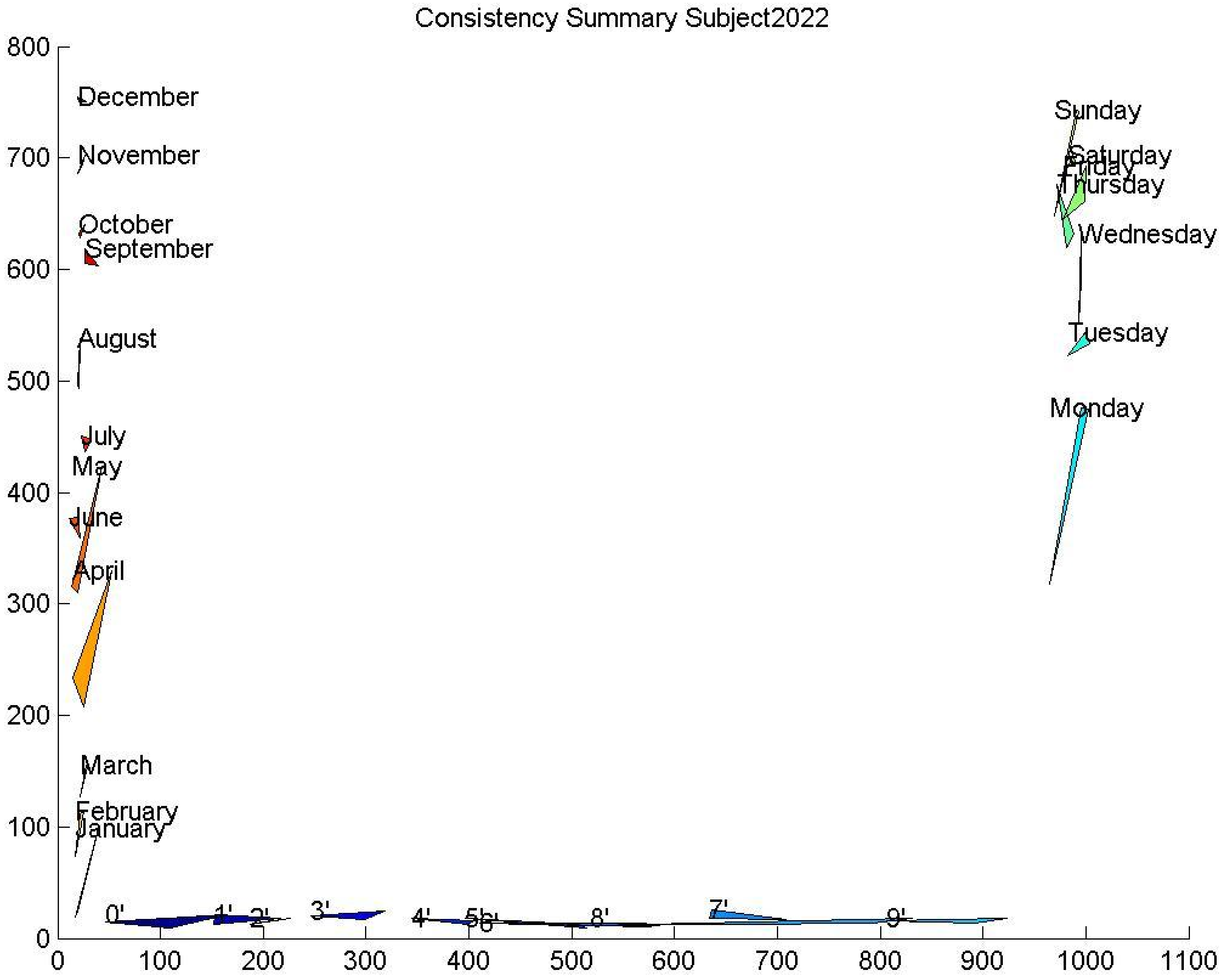

Consistency Summary Subject2022
December
November
October
September
August
July
May
June
April
March
February
January
Sunday
Saturday
Friday
Thursday
Wednesday
Tuesday
Monday
0'
1'
2'
3'
4'
5'
6'
7'
8'
9'
0
100
200
300
400
500
600
700
800
900
1000
1100

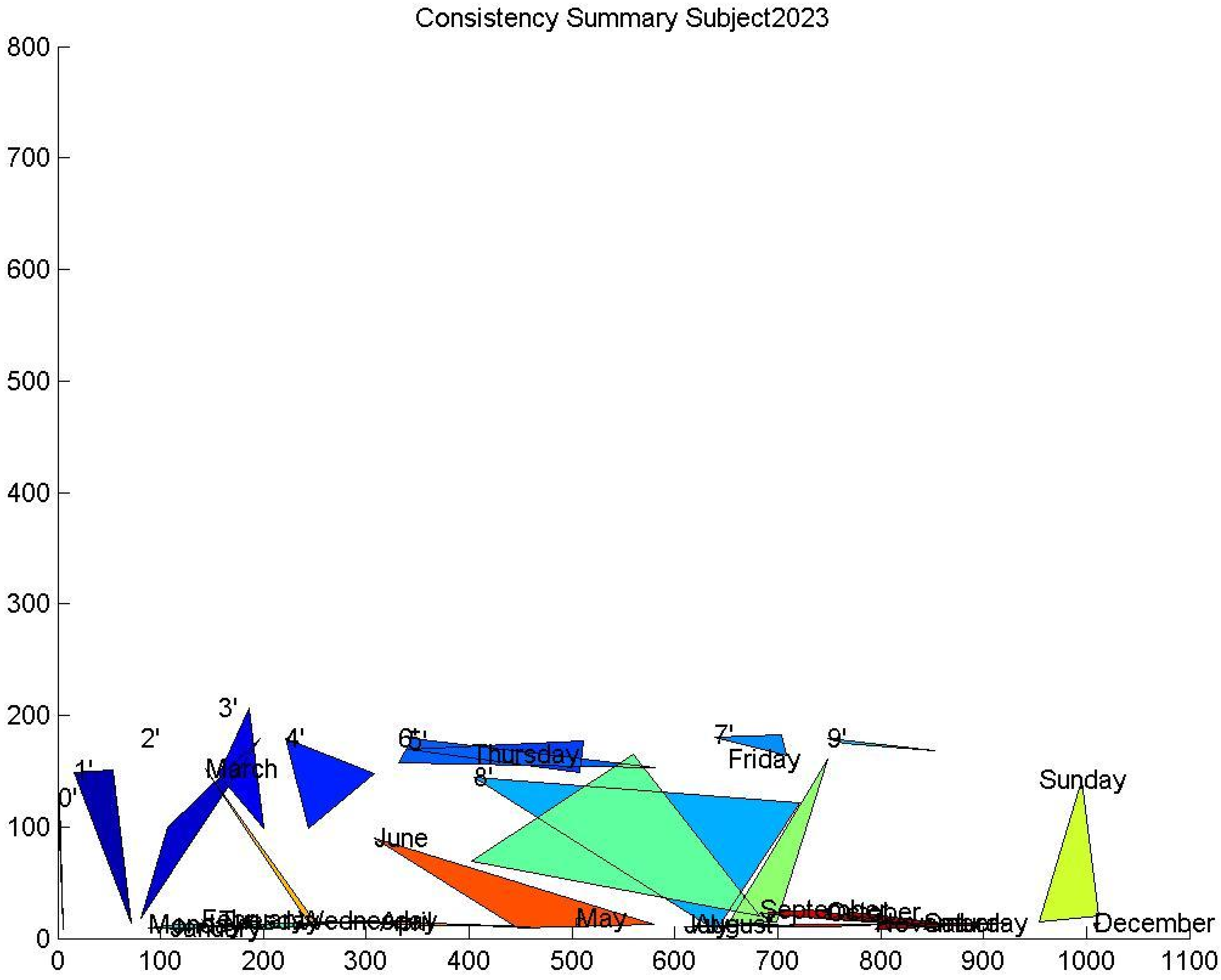
Consistency Summary Subject2023
800
700
600
500
400
300
200
100
0
0
100
200
300
400
500
600
700
800
900
1000
1100
0'
1'
2'
3'
4'
7'
8'
9'
March
June
Thursday
Friday
Sunday
May
August
December
